# A Fluorescent Ditopic Rotaxane Ion‐Pair Host

**DOI:** 10.1002/anie.201713105

**Published:** 2018-03-05

**Authors:** Mathieu Denis, Lei Qin, Peter Turner, Katrina A. Jolliffe, Stephen M. Goldup

**Affiliations:** ^1^ Chemistry University of Southampton Highfield Southampton SO17 1BJ UK; ^2^ School of Chemistry The University of Sydney Sydney NSW 2006 Australia

**Keywords:** anions, fluorescence, mechanical bonds, rotaxanes, sensors

## Abstract

We report a rotaxane based on a simple urea motif that binds Cl^−^ selectively as a separated ion pair with H^+^ and reports the anion binding event through a fluorescence switch‐on response. The host selectively binds Cl^−^ over more basic anions, which deprotonate the framework, and less basic anions, which bind more weakly. The mechanical bond also imparts size selectivity to the ditopic host.

Threading molecules through one another to form an interlocked architecture creates a well‐defined three‐dimensional space in which functional groups can be displayed. These functional groups often mediate attractive intercomponent interactions. Manipulation of these interactions in the design of molecular machines has led to significant advances in molecular shuttles, motors, ratchets, and pumps.^1^ Less well‐studied is the use of interlocked molecules as scaffolds for the development of hosts and sensors. Indeed, the majority of reported interlocked molecules that do display a useful output in response to a small‐molecule binding event are relatively structurally complex molecular shuttles,^2^ with the attendant limitations on their synthetic accessibility. Furthermore, the response to confounding analytes is typically not reported.

The stand‐out exception to this is the use of interlocked molecules to bind and detect anions. Beer and co‐workers^3^ have exploited anion binding extensively in the assembly of rotaxanes and catenanes by employing the anion to template formation of a mechanical bond. The interactions that assembled the host “live on” in the product, allowing these catenanes and rotaxanes to bind anions with a selectivity determined in part by the size and shape complementarity of the host and guest. By tethering an electroactive or photoactive unit to the host, Beer and co‐workers have developed a small number of interlocked anion sensors.^4^

The anion‐responsive sensors reported by Beer and co‐workers typically rely on the same interactions for anion binding that are used in the formation of the mechanical bond.^5^ Although effective, this can be limiting since only arrangements of anion‐binding functionality that are productive in the formation of the interlocked molecule can be applied as hosts. Herein, we report an alternative approach to anion‐responsive fluorescent rotaxanes, in which the mechanical bond is used to alter the properties of simple anion‐binding unit that plays no role in the rotaxane synthesis. As a consequence of the mechanical bond, significant differences were observed in the anion binding behavior of the rotaxane, resulting in a host that is selective for binding Cl^−^ over more (F^−^) or less (Br^−^) basic anions. The crowded environment of the mechanical bond presents other weak non‐covalent interactions, in addition to a urea‐based anion binding unit, and appears to impart restricted access to the binding pocket based on anion size.

Rotaxane **1** (Figure 1 a) was synthesized in 92 % yield using our small‐macrocycle modification^6^ of Leigh's active‐template^7^ Cu‐mediated alkyne–azide cycloaddition (AT‐CuAAC) reaction^8^, ^9^ (see the Supporting Information). The design of rotaxane **1** is based on previous reports of the naphthalimide urea core for the binding and transport of anions.^10^ ^1^H NMR analysis of rotaxane **1** provided evidence that the bipyridine unit H‐bonds to the urea moiety; the NH proton H_1_ resonates at a higher chemical shift in rotaxane **1** (Figure 1 b, spectrum ii) than axle **2** (spectrum i).^11^ This is consistent with the solid‐state structure of **1** found by single‐crystal X‐ray diffraction (SCXRD; Figure 2 a), in which the macrocycle encircles the urea moiety with N‐H⋅⋅⋅N distances of 2.32, 2.58, 2.44, and 2.88 Å. The UV/Vis spectra of **1** and **2** display absorbances at 402 nm and 386 nm in CHCl_3_/CH_3_CN (1:1), respectively, which are attributed to the naphthalimide fluorophore, thus suggesting that H‐bonding from the urea contributes to a red‐shift of the absorbance. In contrast, **1** and **2** exhibit emissions at 470 and 474 nm, respectively, thus suggesting that the mechanical bond does not significantly affect the fluorescence of the urea naphthalimide unit.

**Figure 1:**
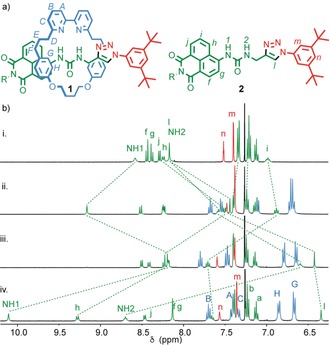
a) Structure of rotaxane **1** and non‐interlocked axle **2** (R=CH_2_C(H)Ph_2_). b) Partial ^1^H NMR of i) **2**, ii) **1**, iii) **1⋅**HBF_4_, iv) **1⋅**HBF_4_+TBACl (2 equiv). 400 MHz, 1:1 v/v CDCl_3_/CD_3_CN, 298 K; peak assignment as in (a).

Titration of axle **2** with the tetrabutylammonium (TBA) salt of AcO^−^ led to increasing downfield shifts of the signals for the NH protons H_1_ and H_2_, a red shift of the absorbance at 386 nm to 400 nm, and quenching of the emission associated with the naphthalimide. Non‐linear‐regression analysis of the ^1^H NMR, UV/Vis and fluorescence titration data (see the Supporting Information) fit well to 1:1 binding models, thereby allowing binding constants to be determined (Table 1). Similar effects were observed for a range of anions with the observed order of anion affinity found to be I^−^<Br^−^<HSO_4_^−^<TsO^−^<MsO^−^<Cl^−^<F^−^<AcO^−^; a trend in keeping with their H‐bond‐acceptor strengths.^12^

**Table 1:** Binding constants for non‐interlocked axle **2** and rotaxane **1⋅**HBF_4_.

	Binding constants (*K*_a_) [m ^−1^]	
Anion	**2**	**1**.HBF_4_
F^−^	4930^[a]^	–^[b]^
Cl^−^	1780^[a]^	>10^4^ (28 000^[c]^)
Br^−^	390^[a]^	4660^[a]^
I^−^	70^[a]^	580^[a]^
AcO^−^	7770^[a]^	–^[b]^
HSO_4_^−^	610^[a]^	2270^[a]^
TsO^−^	690^[a]^	1514^[a]^
MsO^−^	950^[a]^	2600^[a]^

Titration experiments were carried out in CDCl_3_/CD_3_CN (1:1). *K*_a_ determined by non‐linear regression analysis (RMS error <15 %, see the Supporting Information). Anions were added as TBA salts. [a] Determined by ^1^H NMR (*c*=2.5 mm). [b] Host deprotonation observed. [c] Determined by UV/Vis spectroscopy (*c*=0.13 mm).

We anticipated that addition of H‐bond‐accepting anions to rotaxane **1** would lead to displacement of the bipyridine–urea interaction and that this competition between inter‐ and intra‐molecular H‐bonding might impart selectivity to **1** that is different from axle **2**. However, titration of **1** with a panel of anions led to no observable change by ^1^H NMR, UV/Vis, or fluorescence spectroscopy, thus suggesting that the NH⋅⋅⋅anion interaction is unable to compete with the inter‐component H‐bonds.

The inhibition of anion binding in **1** corresponds to Lewis basic inhibition of the receptor. We have previously observed inhibition of an interlocked Au^I^ catalyst due to a similar Lewis basic interaction of the bipyridine moiety with the metal ion.^13^ In that case, catalytic activity was restored through binding of cations into the cavity of the rotaxane, and we speculated that a similar interaction between a cation and the bipyridine ring might be used to turn on anion binding by **1**. As a proof of concept, we investigated whether protonation of the bipyridine moiety could lead to binding of exogenous anions by the urea moiety. Furthermore, binding of anions by [**1**H]^+^ would correspond to ditopic binding of HX, which is relatively unusual;^14^ although anion binding motifs are known in which the host requires protonation, the donated proton is typically part of the anion coordination sphere.^15^ In contrast, we anticipated that the proton would be sequestered in the rotaxane cavity, leading to separated ion‐pair binding.^16^, ^17^

When **1** was treated with an aqueous solution of HBF_4_, a new species with a significantly different ^1^H NMR spectrum (Figure 1 b, spectrum iii) was obtained, which was assigned as **1⋅**HBF_4_. Key changes include an upfield shift of the signals attributable to NH protons H_1_ and H_2,_ which suggests that they are no longer involved in H‐bonding to the bipyridine unit, and an upfield shift of the signal attributable to H_l_, which is consistent with the presence of a CH⋅⋅⋅π contact in the protonated rotaxane. The UV/Vis spectrum of rotaxane **1** also changes upon protonation; a new absorbance appeared at 310 nm that was assigned to the protonated bipyridine moiety,^18^ and the absorbance band attributable to the naphthalimide blue‐shifted to 381 nm, which is consistent with the urea moiety no longer being involved in H‐bonding interactions.

SCXRD analysis confirmed the formation of the HBF_4_ salt and revealed interactions consistent with the solution‐phase data; protonation causes large‐scale structural rearrangement to a (co)conformation in which one bipyridine N is protonated and engaged in a hydrogen bond with N3 of the triazole and, as a result, H_l_ is held in close proximity to the face of one of the macrocycle aromatic rings. The naphthalamide residue of **1**⋅HBF_4_ was found to be disordered about two orientations, one of which exhibits a short face–face contact between one of the bipyridine rings and the naphthalimide unit (Figure 2 b). Thus, at least in the solid state, protonation also seems to induce π‐stacking of the bipyridine moiety and the naphthalimide ring.^19^ Furthermore, in the solid state, the BF_4_^−^ anion interacts with the urea protons, H_h_ of the naphthalimide, and one of H_G_ (Figure 2 b).

**Figure 2:**
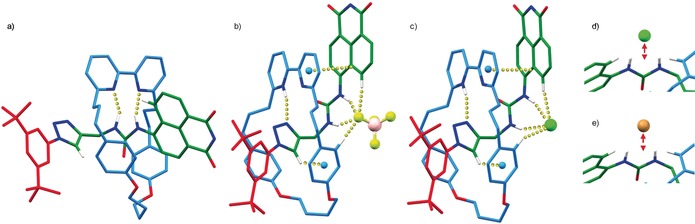
SCXRD structures with selected angles [°] and distances [Å] of a) **1** (NH_1_⋅⋅⋅N=2.45, NH_*2*_⋅⋅⋅N=2.32, C‐N_1_‐C‐C_*ipso*_=16.1), b) **1⋅**HBF_4_ (NH_1_⋅⋅⋅F=1.99, NH_2_⋅⋅⋅F=2.16, CH_*G*_⋅⋅⋅F=2.56, *CH_h_*⋅⋅⋅F=2.16, H(py)⋅⋅⋅N=2.05, H_l_⋅⋅⋅centroid=2.91, centroid⋅⋅⋅naphthalimide=3.39, C‐N_1_‐C‐C_*ipso*_=11.83), c) **1⋅**HCl (NH_*1*_⋅⋅⋅Cl=2.24, NH_*2*_⋅⋅⋅Cl=2.42, CH_*G*_⋅⋅⋅Cl=2.92, *CH_h_*⋅⋅⋅Cl=2.59, H(py)⋅⋅⋅N=2.64, H_l_⋅⋅⋅centroid=2.80, centroid⋅⋅⋅naphthalimide=3.42, C‐N_1_‐C‐C_*ipso*_=32.43). Anion binding unit showing the displacement of the anion from the H_1_‐H_2_‐H_*G*_‐H_*h*_ plane for d) **1⋅**HCl (1.61 Å) and e) **1⋅**HBr (1.84 Å). 1,1′‐Diphenylethyl substituents omitted for clarity.

The solid‐state structure of **1⋅**HBF_4_ suggests that the urea NHs are no longer encumbered by the bipyridine donors and are thus available to bind exogenous anions.^20^ Titration of **1⋅**HBF_4_ with basic anions such as AcO^−^ or F^−^ led to deprotonation of the host to regenerate **1**, as determined by ^1^H NMR and UV/Vis analysis, and thus no anion binding.^21^ Conversely, when **1⋅**HBF_4_ was treated with TBACl, the signals attributable to NH protons H_1_ and H_2_ shift downfield upon addition of the anion (Figure 1b, spectrum iv), which is consistent with H‐bonding of Cl^−^ to the urea moiety. Simultaneously, *peri* proton H_h_ also shifts downfield, which is consistent with a CH⋅⋅⋅Cl^−^ hydrogen bond, and triazole proton H_l_ shifts upfield, thus suggesting that the CH⋅⋅⋅π contact becomes stronger. SCXRD analysis of crystals grown from a solution of **1⋅**HBF_4_ in CH_2_Cl_2_/MeCN (1:1 v/v) in the presence of TBACl (10 equiv) revealed that Cl^−^ is bound as expected by the urea moiety in the protonated host with an additional C−H⋅⋅⋅Cl^−^ contact with H_h_ (Figure 2 c) and a longer contact with H_G_. The CH⋅⋅⋅π contact between H_l_ and the flanking aromatic is also shorter than in **1** (Δ*d*=0.15 Å), which is consistent with the solution‐state data.

The titration of **1⋅**HBF_4_ with anions could also be followed by UV/Vis and fluorescence spectroscopy. Addition of Cl^−^ resulted in a red shift (Δ*λ*=15 nm) and increase in the absorbance at 380 nm, and a 2‐fold increase in the naphthalimide emission. Titrations with Br^−^, HSO_4_^−^, MsO^−^, or TsO^−^ revealed similar, although less pronounced changes. Although I^−^ showed similar changes by ^1^H NMR and UV/Vis spectroscopy, the emission was quenched, presumably due to Stern–Volmer collisional effects.^22^

Comparison of the binding constants determined for **1⋅**HBF_4_ with those of **2** reveal a number of clear differences. Firstly, the potential for deprotonation of **1⋅**HBF_4_, which renders it insensitive to anions, ensures that, whereas **2** binds more basic anions more strongly, this is not the case for **1⋅**HBF_4_, which fails to bind the more basic F^−^ and AcO^−^ guests. Secondly, binding of the less basic anions is much stronger to **1⋅**HBF_4_ than to the neutral host **2**. This is unsurprising since charge–charge interactions are expected to stabilize the interlocked complex significantly.^23^

The relative order of binding strength is also different for **2** and **1⋅**HBF_4_. Whereas binding runs in the order I^−^<Br^−^< HSO_4_^−^<TsO^−^<MsO^−^<Cl^−^ for **2**, the relative preference for the sulfonate versus halide anions is lower for **1⋅**HBF_4_, resulting in the order I^−^<HSO_4_^−^<TsO^−^<MsO^−^<Br^−^<Cl^−^. The relative preference of **1⋅**HBF_4_ for MsO^−^ over TsO^−^ is also higher. These results suggest that the crowded environment resulting from the presence of the threaded macrocycle adjacent to the urea motif in **1⋅**HBF_4_ provides some size and shape selection; the spherical Br^−^ anion (ionic radius=168 pm)^24^ is preferred over the tetrahedral HSO_4_^−^ and the larger MsO^−^ and TsO^−^ anions. Comparison of the solid‐state structure of **1⋅**HCl and **1⋅**HBr (Figure 2 d) suggests that as the anionic radius increases, the “fit” of the anion between the urea nitrogen protons, *peri* proton H_h_, and macrocycle proton H_G_ decreases, forcing the anion out of the plane of the four H–anion contacts.

Finally, it is noteworthy that **1⋅**HBF_4_ exhibits a fluorescent switch‐on response upon anion binding, whereas axle **2** exhibits a switch‐off response. The origin of this photophysical difference is not obvious; in both cases binding of the anion is expected to increase the electron density in the naphthalimide fluorophore and, on simple charge transfer grounds, would be expected to enhance the stability of the excited state, whereas the proximity of anions has previously been reported to result in PET quenching.^25^ The explanation probably lies in the naphthalimide–bipyridine π–π interaction observed in the solid‐state structure of **1⋅**HBF_4_, which is necessarily absent in the case of **2**. Anion binding may affect this interaction by rigidifying the framework in some way, thus reducing non‐radiative decay linked to bond rotation.

In conclusion, we have demonstrated that the AT‐CuAAC reaction can be used to synthesize interlocked hosts for anions in which functional groups used or generated during the method of synthesis are not involved in the binding of the guest, thereby opening up new targets for study. In doing so, we serendipitously discovered a rotaxane framework in which anion binding is activated allosterically by protonation, leading to a system that acts as a ditopic host for an HX ion pair. The binding event is reported by a clear fluorescence response, and anion selectivity is determined both by the strength of the H‐bonding interaction between the host and anion, and the anion p*K*_a_. Furthermore, the mechanical bond introduces size selectivity into this receptor.

## Conflict of interest

The authors declare no conflict of interest.

## Supporting information

As a service to our authors and readers, this journal provides supporting information supplied by the authors. Such materials are peer reviewed and may be re‐organized for online delivery, but are not copy‐edited or typeset. Technical support issues arising from supporting information (other than missing files) should be addressed to the authors.

Supplementary
