# Supplementary material for: A Fluorescent Ditopic Rotaxane Ion‐Pair Host
Source: Angew Chem Int Ed Engl. 2018 Mar 5;57(19):5315–9. doi: 10.1002/anie.201713105 (PMC5947583; doi:10.1002/anie.201713105)
Supplement: Supplementary file 1 — Supplementary [file ANIE-57-5315-s001.pdf]

Angewandte Chemie
Eine Zeitschrift der Gesellschaft Deutscher Chemiker

Supporting Information

A Fluorescent Ditopic Rotaxane Ion-Pair Host

Mathieu Denis+, Lei Qin+, Peter Turner, Katrina A. Jolliffe, and Stephen M. Goldup**

anie_201713105_sm_miscellaneous_information.pdf

Contents

General Experimental 3
Experimental Procedures 4
2-(2,2-Diphenylethyl)-6-nitro-1H-benzo[de]isoquinoline-1,3(2H)-dione (S3) 4
2-(2,2-Diphenylethyl)-6-amino-1H-benzo[de]isoquinoline-1,3(2H)-dione (S4) 5
Urea alkyne (S5) 7
Rotaxane 1 10
Rotaxane 1.HBF_4 13
Axle 2 17
NMR and fluorescence titration data 20
^1H NMR titrations of axle 2 21
UV-vis titrations of axle 2 29
Fluorescent titrations of axle 2 31
^1H NMR titrations of rotaxane 1 33
^1H NMR titrations of rotaxane 1.HBF_4 34
UV-Vis titrations of rotaxane 1·HBF_4 41
Fluorescence titrations of rotaxane 1·HBF_4 45
Single Crystal X-ray Analysis Data 49
References 59

General Experimental

Unless otherwise stated, all reagents were purchased from commercial sources and used without further purification. All reactions were carried out under an atmosphere of N_2 using anhydrous solvents unless otherwise stated. Anhydrous solvents were obtained by passing the solvent through an activated alumina column on an MBRAUN MB SPS-800 solvent purification system. Petrol refers to the fraction of petroleum ether boiling in the range 40-60 °C. IPA refers to iso-propyl alcohol. EDTA-NH_3 solution refers to an aqueous solution of NH_3 (17% w/w) saturated with sodium-ethylenediaminetetraacetate. Flash column chromatography was performed using a Biotage Isolera-4 automated chromatography system, employing Biotage SNAP or ZIP cartridges. Analytical TLC was performed on precoated silica gel plates (0.25 mm thick, 60F254, Merck, Germany) and observed under UV light. NMR spectra were recorded on a Bruker AV400, AV3-400, AV500 or Bruker AV600 instrument, at a constant temperature of 298 K. Chemical shifts are reported in parts per million from low to high field and referenced to residual solvent. Standard abbreviations indicating multiplicity were used as follows: m = multiplet, quint = quintet, q = quartet, t = triplet, d = doublet, s = singlet, app. = apparent, br = broad. All melting points were determined using a Griffin apparatus and are uncorrected. Low resolution mass spectrometry was carried out either by the mass spectrometry services at the Queen Mary University of London using an Agilent SL Ion Trap MSD instrument or using a Waters TQD mass spectrometer equipped with a triple quadrupole analyser with UHPLC injection [BEH C18 column; MeCN-hexane gradient {0.2% formic acid}]. High resolution mass spectrometry was carried out either by the EPSRC National Mass Spectrometry in Swansea or by the mass spectrometry services at the University of Southampton with samples were analysed using a MaXis (Bruker Daltonics, Bremen, Germany) mass spectrometer equipped with a Time of Flight (TOF) analyser. Samples were introduced to the mass spectrometer via a Dionex Ultimate 3000 autosampler and uHPLC pump. Gradient 20% acetonitrile (0.2% formic acid) to 100% acetonitrile (0.2% formic acid) in five minutes at 0.6 mL min. Column, Acquity UPLC BEH C18 (Waters) 1.7 micron 50 × 2.1mm.

The following compounds were synthesised according to literature procedures:
3,5-di-*tert*-butylphenyl azide (**S1**),[1] and macrocycle **S2**.[22]

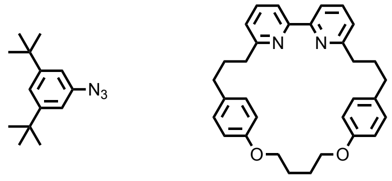

S1 **S2**

Experimental Procedures

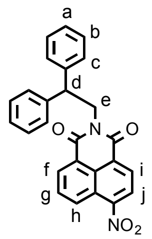

2-(2,2-Diphenylethyl)-6-nitro-1H-benzo[de]isoquinoline-1,3(2H)-dione (S3)

4-Nitro-1,8-naphthalic anhydride (0.5 g, 2.1 mmol, 1 equiv.) was dissolved in EtOH (15 mL). Diphenyl ethylamine (0.49 g, 2.5 mmol, 1.2 equiv.) was added to the solution and refluxed at 80 °C for 18 h. After cooling the reaction mixture to r.t., the solvent was removed *in vacuo* and purified by flash column chromatography (1:1 Petrol/ CH_2Cl_2) to give nitro-compound **S3** as a yellow foam (0.75 g, 86%). ^1H NMR ($CDCl_3$, 400 MHz, 298 K) δ 8.79 (dd, *J* = 8.7, 1.0, 1H, H_h), 8.64 (dd, *J* = 7.3, 1.0, 1H, H_f), 8.58 (d, *J* = 8.0, 1H, H_j), 8.34 (d, *J* = 8.0, 1H, H_i), 7.93 (dd, *J* = 8.7, 7.4, 1H, H_g), 7.37-7.32 (m, 4H, H_c), 7.27-7.21 (m, 4H, H_b), 7.18-7.13 (m, 2H, H_a), 4.89-4.85 (m, 2H, H_e), 4.82-4.71 (m, 1H, H_d). ^{13}C NMR ($CDCl_3$, 101 MHz, 298 K) δ 163.3, 162.4, 149.5, 141.3, 132.4, 129.9, 129.7, 129.3, 128.9, 128.4, 128.4, 126.8, 126.7, 123.8, 123.6, 122.8, 48.5, 44.8, 34.1, 22.3, 14.1. IR: (ν_{max}/cm^{-1}) 3405, 2905, 1692, 1620, 1577, 1520, 1375, 1313, 1226, 1181, 989. M.p. (°C) 40-43. HRMS (ESI+) m/z = 423.1336 $[M+H]^+$ (calc. for $C_{26}H_{19}N_2O_4$ 423.1339). UV: λ_{max}(MeCN)/nm (ε/ $mol^{-1}cm^{-1}dm^3$) 347 (10978).

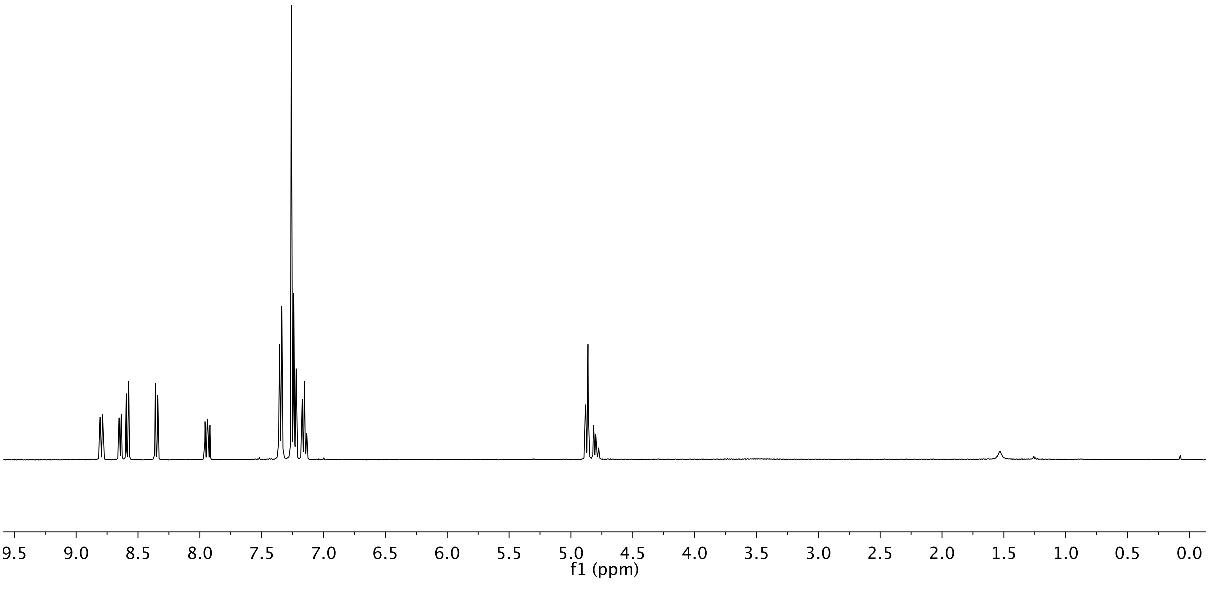

Figure S1 ^1H NMR ($CDCl_3$, 400 MHz, 298 K) of **S3**.

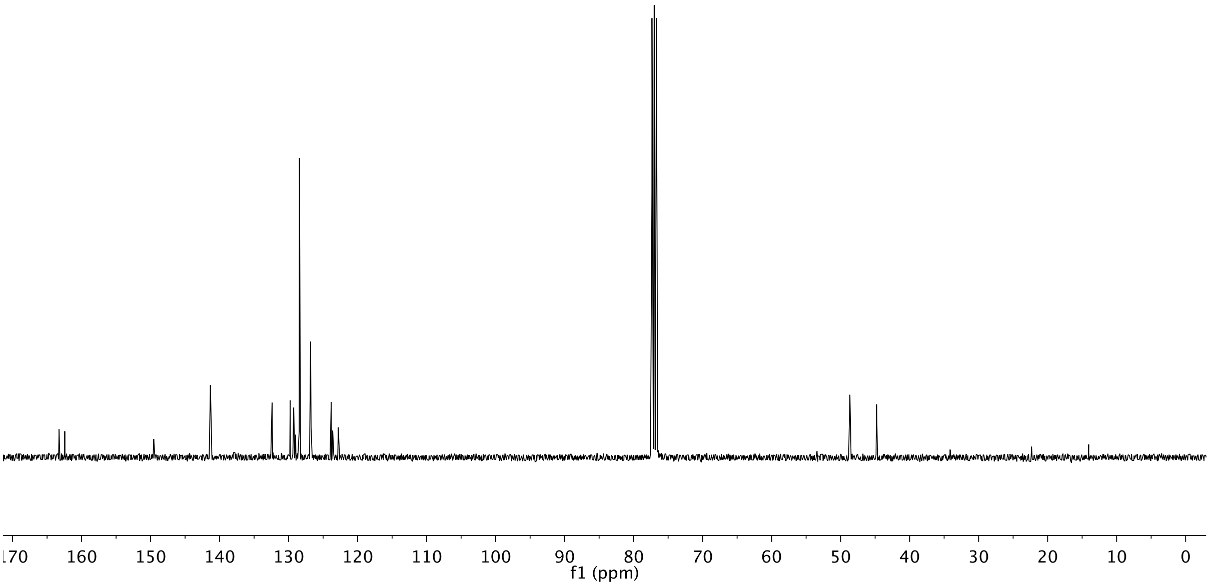

Figure S2 ^{13}C NMR ($CDCl_3$, 101 MHz, 298 K) of **S3**.

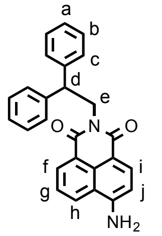

2-(2,2-Diphenylethyl)-6-amino-1H-benzo[de]isoquinoline-1,3(2H)-dione (S4)

Nitro-compound **S3** (0.36 g, 0.95 mmol, 1 equiv.) was hydrogenated in MeOH/EtOAc (1:1, 20 mL) at r.t. under a hydrogen atmosphere, using a Pd/C catalyst (5%wt, 0.20 g, 0.095 mmol, 0.1 equiv.). The reaction was monitored by TLC until all starting material had been consumed (~4 h). The reaction mixture was filtered through Celite, washed with MeOH/EtOAc (1:1), and the solvent evaporated *in vacuo* to yield aniline **S4** as a yellow solid (0.36 g, 97%). ^{1}H NMR ($(CD_3)_2SO$, 400 MHz, 298 K) δ 8.57 (d, J = 8.0, 1H, H_h), 8.34 (d, J = 6.8, 1H, H_f), 8.12 (d, J = 8.4, 1H, H_i), 7.60 (dd, J = 7.7, 8.0, 1H, H_g), 7.42 (br s, 1H, -NH_2-), 7.35-7.30 (m, 4H, H_c), 7.26-7.20 (m, 4H, H_b), 7.16-7.11 (m, 2H, H_a), 7.80 (d, J = 8.4, 1H, H_j), 4.73-4.69 (m, 3H, H_d and H_e).^{13}C NMR ($(CD_3)_2SO$, 101 MHz, 298 K) δ 163.8, 162.9, 152.6, 142.2, 133.9, 130.9, 129.6, 129.3, 128.2, 128.0, 126.4, 123.9, 121.6, 119.2, 108.1, 107.3, 48.4, 43.3. IR: (ν_{max}/cm^{-1}) 3505, 3349, 3225, 2358, 1648, 1592, 1375, 1246, 1017. 982. M.p. (°C) 95-96. HRMS (ESI+) m/z = 393.1599 [M+H^{+}] (calc. for $C_{26}H_{21}N_2O_2$ 393.1598). [M+H^{+}]. UV: λ_{max}(MeCN)/nm (ε / mol^{-1}cm^{-1}dm^{3}) 428 (3226).

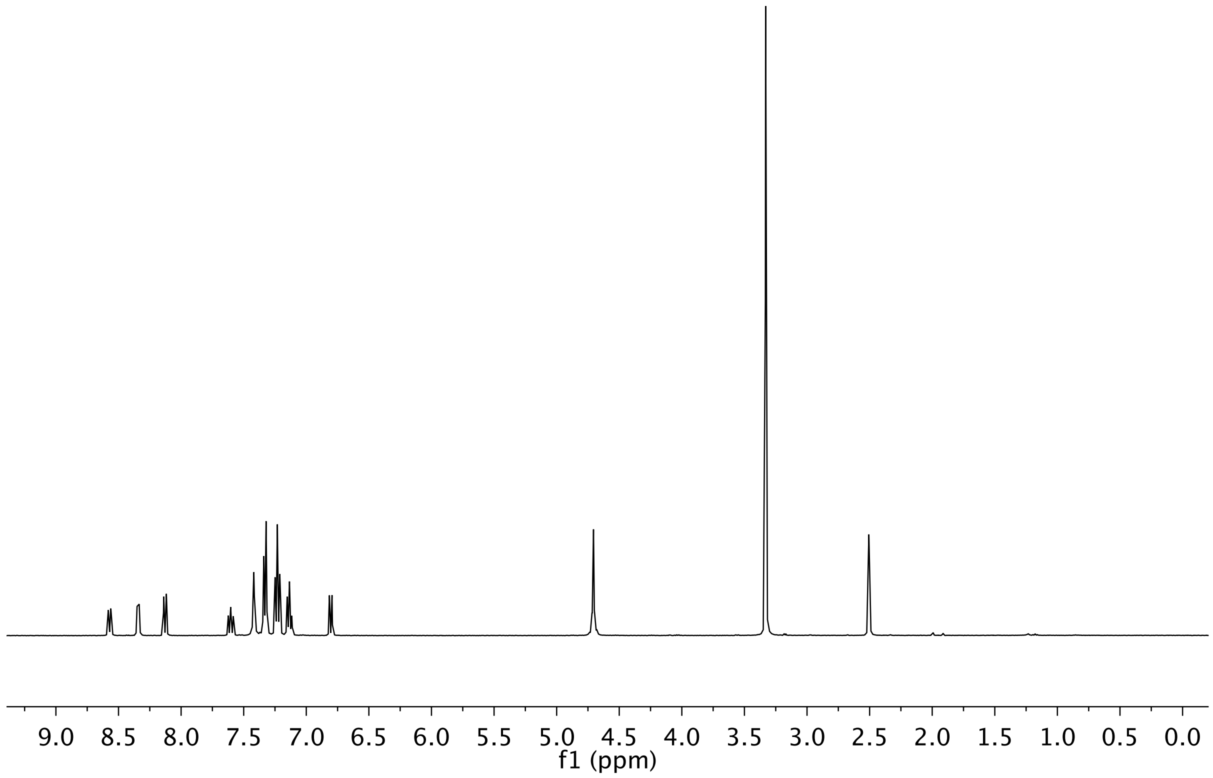

Figure S3 ^{1}H NMR ($(CD_3)_2SO$, 400 MHz, 298 K) of **S4**.

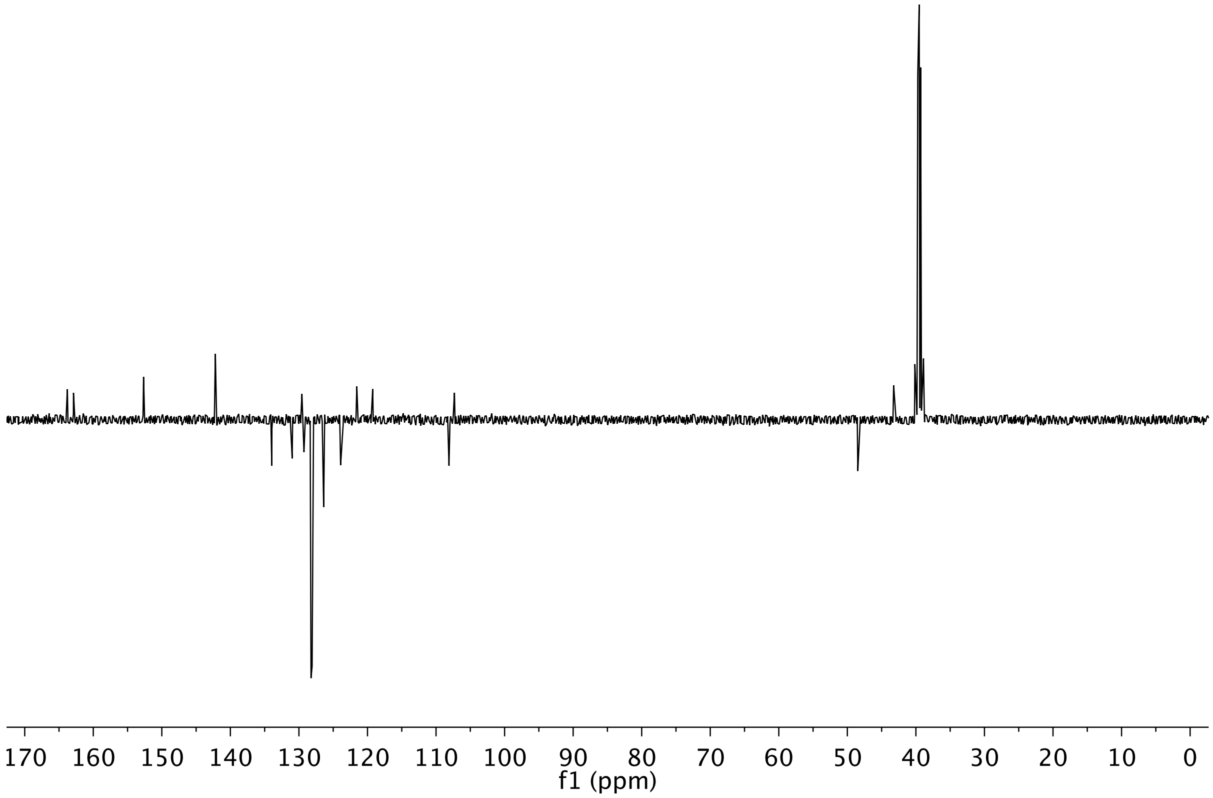

Figure S4 JMOD NMR ($(CD_3)_2SO$, 101 MHz, 298 K) of **S4**.

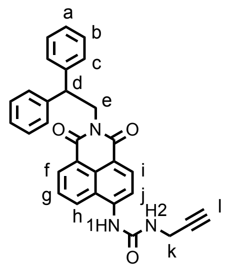

Urea alkyne (S5)

To a stirred suspension of amine **S4** (98 mg, 0.25 mmol, 1 equiv.) and DMAP (61 mg, 0.5 mmol, 2 equiv.) in CH_2Cl_2 (10 mL) at -10ºC was added triphosgene (24 mg, 0.08 mmol, 0.33 equiv.) as a solution in toluene (1 mL). The resulting solution was kept -10 ºC for 1 h. Propargylamine (32 µL, 0.5 mmol, 2 equiv.) was then added and the mixture allowed to stir at room temperature for 3 h. The solvent was concentrated *in vacuo* and the crude residue purified *via* flash column chromatography on silica gel using a linear gradient of EtOAc (5 – 20%) in petrol, affording pure product **S5** as a pale yellow solid (77 mg, 65%).^{1}H NMR ((CD_3)$_2$SO, 400 MHz, 298 K) δ 9.31 (s, 1H, -*NH1*-), 8.56 (dd, *J* = 8.6, 1.0, 1H, H_h), 8.46 (d, *J* = 8.5, 1H, H_g), 8.43 (dd, *J* = 7.3, 1.0, 1H, H_j), 8.35 (d, *J* = 8.4, 1H, H_f), 7.83 (dd, *J* = 8.6, 7.3, 1H, H_i), 7.33 (d, *J* = 7.0, 4H, H_c), 7.23 (t, *J* = 7.6, 5H, H_b and -*NH2*-), 7.13 (t, *J* = 7.3, 2H, H_a), 4.80 – 4.61 (m, 3H, H_d and H_e), 4.01 (dd, *J* = 5.6, 2.5, 2H, H_k), 3.20 (t, *J* = 2.5, 1H, H_l) ^{13}C NMR ((CD_3)$_2$SO, 101 MHz, 298 K) δ 163.5, 162.9, 154.1, 142.0, 132.5, 130.8, 128.3, 128.1, 126.5, 126.1, 122.1, 121.9, 114.4, 114.4, 81.4, 73.4, 48.4, 43.5, 28.9. HRMS (ESI+) m/z = 474.1813 [M+H]$^+$ (calc. for $C_{30}H_{24}N_3O_3$ 474.1812).

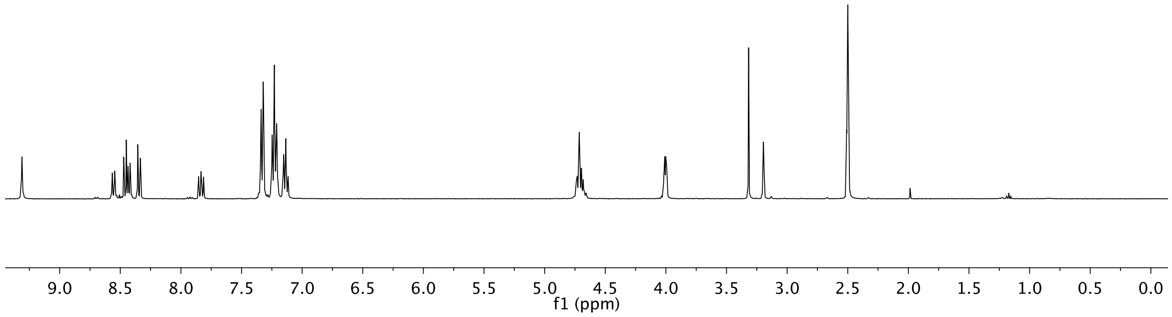

Figure S5 ^{1}H NMR ((CD_3)$_2$SO, 400 MHz, 298 K) of **S5**.

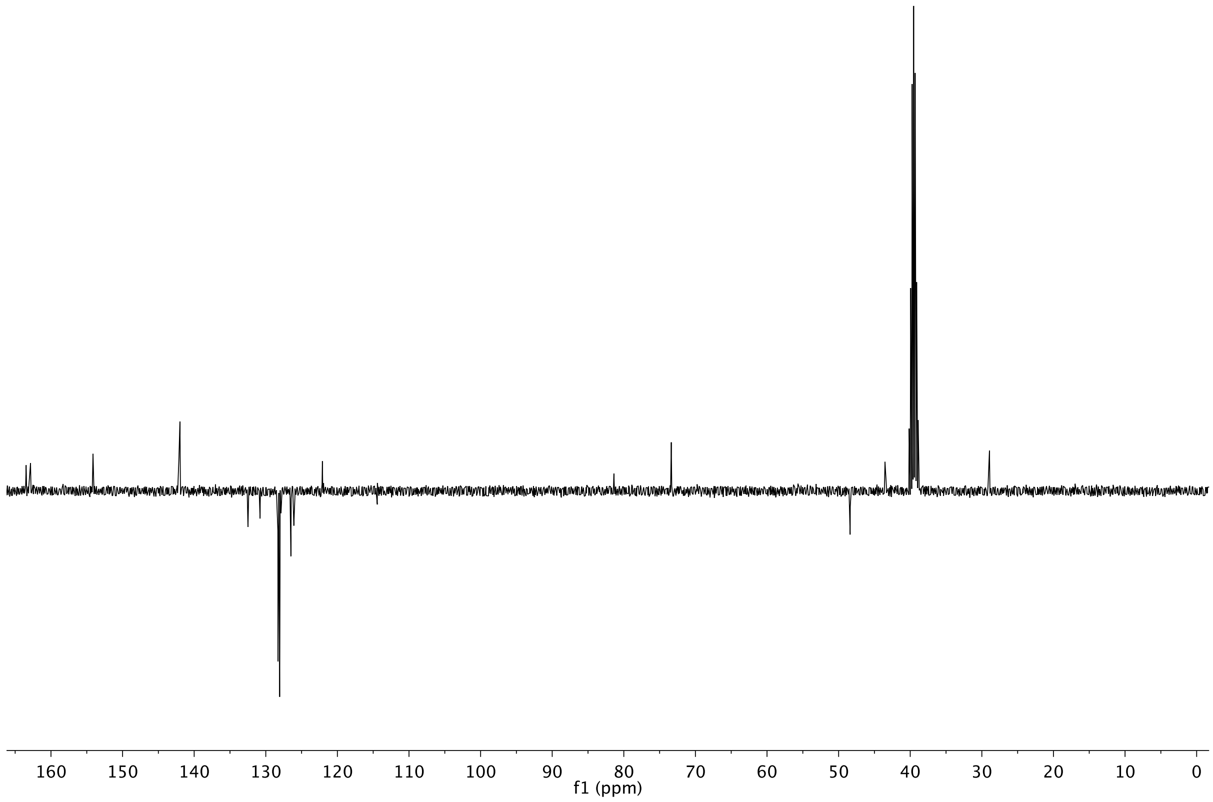

Figure S6 JMOD NMR ($(CD_3)_2SO$, 101 MHz, 298 K) of **S5**.

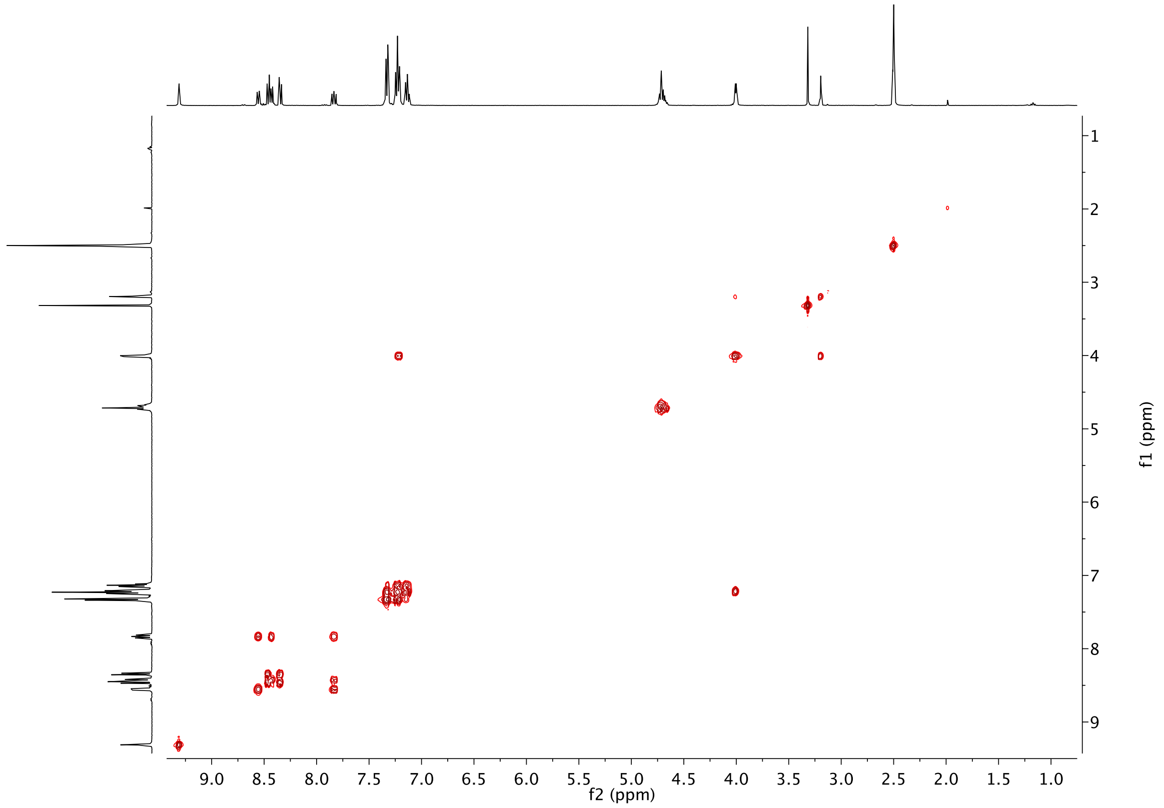

Figure S7 COSY NMR ($(CD_3)_2SO$, 400 MHz, 298 K) of **S5**.

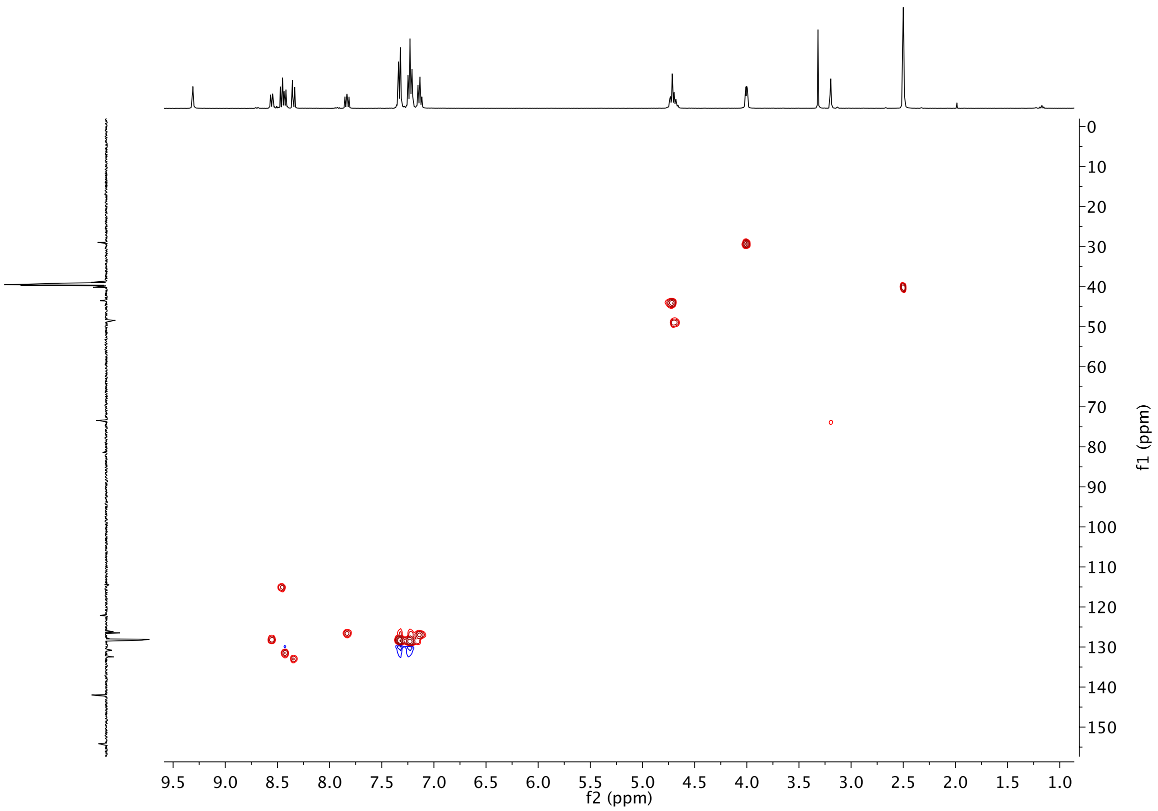

Figure S8 HSQC NMR ($(CD_3)_2SO$, 400 MHz, 298 K) of **S5**.

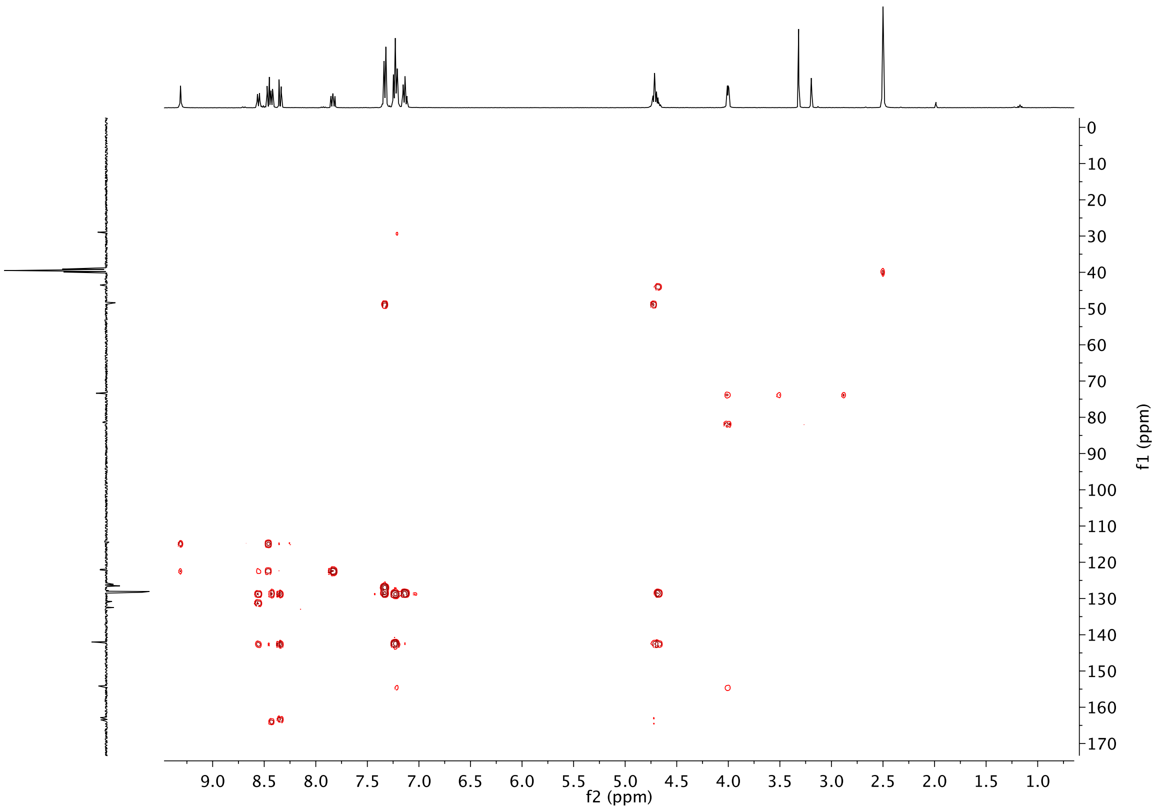

Figure S9 HMBC NMR ($(CD_3)_2SO$, 400 MHz, $(CD_3)_2SO$, 298 K) of **S5**.

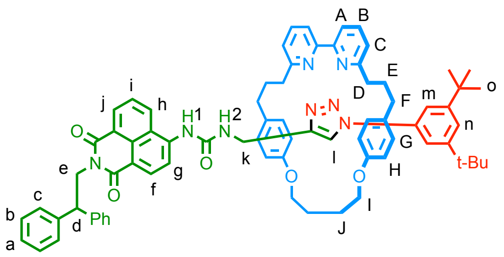

Rotaxane 1

A dry CEM MW vial was charged with macrocycle **S2** (12 mg, 0.025 mmol, 1 equiv.), azide S**1** (5.8 mg, 0.025 mmol, 1 equiv.), alkyne **S5** (12 mg, 0.025 mmol, 1 equiv.), and $[Cu(MeCN)_4]PF_6$ (8.9 mg, 0.024 mmol, 0.96 equiv.). CH_2Cl_2 (1 mL) was added, followed by DIPEA (4.4 µL, 0.025 mmol, 1 equiv.) and the reaction mixture stirred at r.t. for 4 h. NH_3-EDTA (5 mL) was added and the crude extracted with CH_2Cl_2 (3 x 5 mL). The combined organic layers were dried ($MgSO_4$) and concentrated *in vacuo*. The crude was purified *via* flash column chromatography on silica gel with an isocratic elution of petrol/CH_2Cl_2/MeCN/NH_3 46:46:7:1, affording rotaxane **1** as a yellow foam (27 mg, 92%).^{1}H NMR ($CDCl_3$, 400 MHz, 298 K) δ 9.16 (s, 1H, *-NH1-*), 8.51 (d, *J* = 8.5, 1H, H_f), 8.26 – 8.21 (m, 2H, H_j and H_g), 7.67 (t, *J* = 7.8, 2H, H_B), 7.57 (d, *J* = 5.5, 1H, *-NH2-*), 7.54 (d, *J* = 8.0, 2H, H_A), 7.50 (d, *J* = 8.5, 1H, H_h), 7.47 (t, *J* = 1.7, 1H, H_n), 7.44 (s, 1H, H_l), 7.41 – 7.36 (m, 6H, H_c and H_m), 7.23 (t, *J* = 7.9, 4H, H_b), 7.16 – 7.08 (m, 4H, H_C and H_a), 6.88 (dd, *J* = 8.5, 7.3, 1H, H_i), 6.71 (d, *J* = 8.7, 4H, H_H), 6.67 (d, *J* = 8.7, 4H, H_G), 4.92 – 4.80 (m, 3H, H_e and H_d), 4.34 – 4.21 (m, 4H, H_I), 3.81 (d, *J* = 5.5, 2H, H_k), 2.63 – 2.29 (m, 8H, H_D and H_F), 2.23 – 2.10 (m, 4H, H_J), 1.83 – 1.61 (m, 4H, H_E), 1.36 (s, 18H, H_o). ^{13}C NMR ($CDCl_3$, 101 MHz, 298 K) δ 164.8, 164.2, 163.9, 157.7, 156.0, 153.3, 152.8, 146.5, 143.0, 142.2, 138.0, 137.0, 133.7, 132.3, 130.4, 129.3, 129.1, 128.7, 128.4, 127.1, 126.6, 124.4, 122.7, 122.7, 122.6, 122.0, 120.1, 115.3, 115.2, 114.4, 114.1, 66.7, 49.0, 44.4, 36.1, 35.3, 35.0, 34.8, 31.5, 31.4, 25.2. LR-MS (ESI) m/z = 1184.1 $[M+H]^+$.

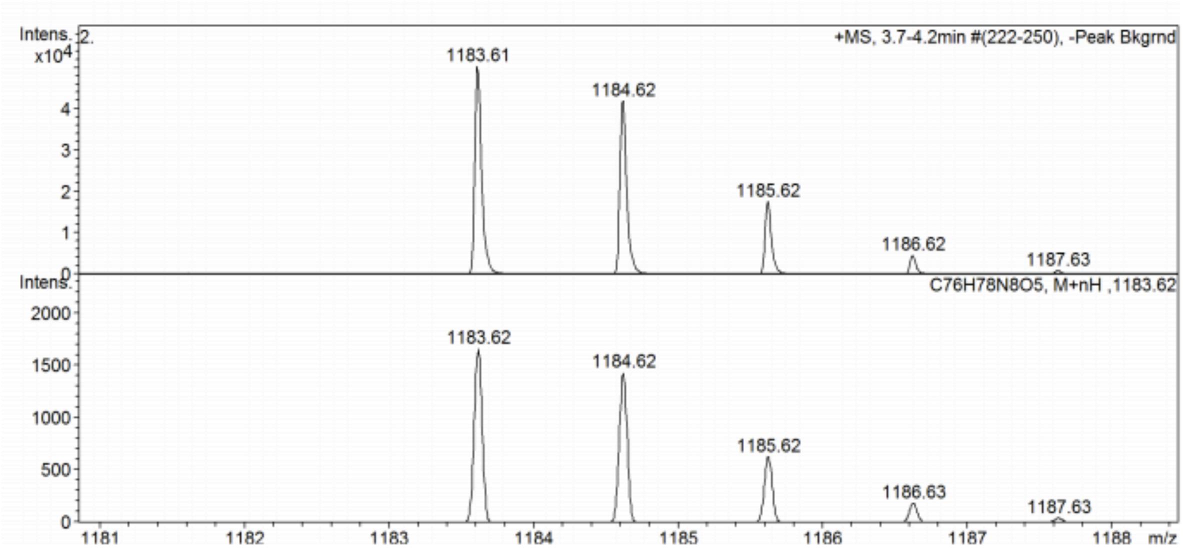

Figure S10 Isotope pattern of **1**.

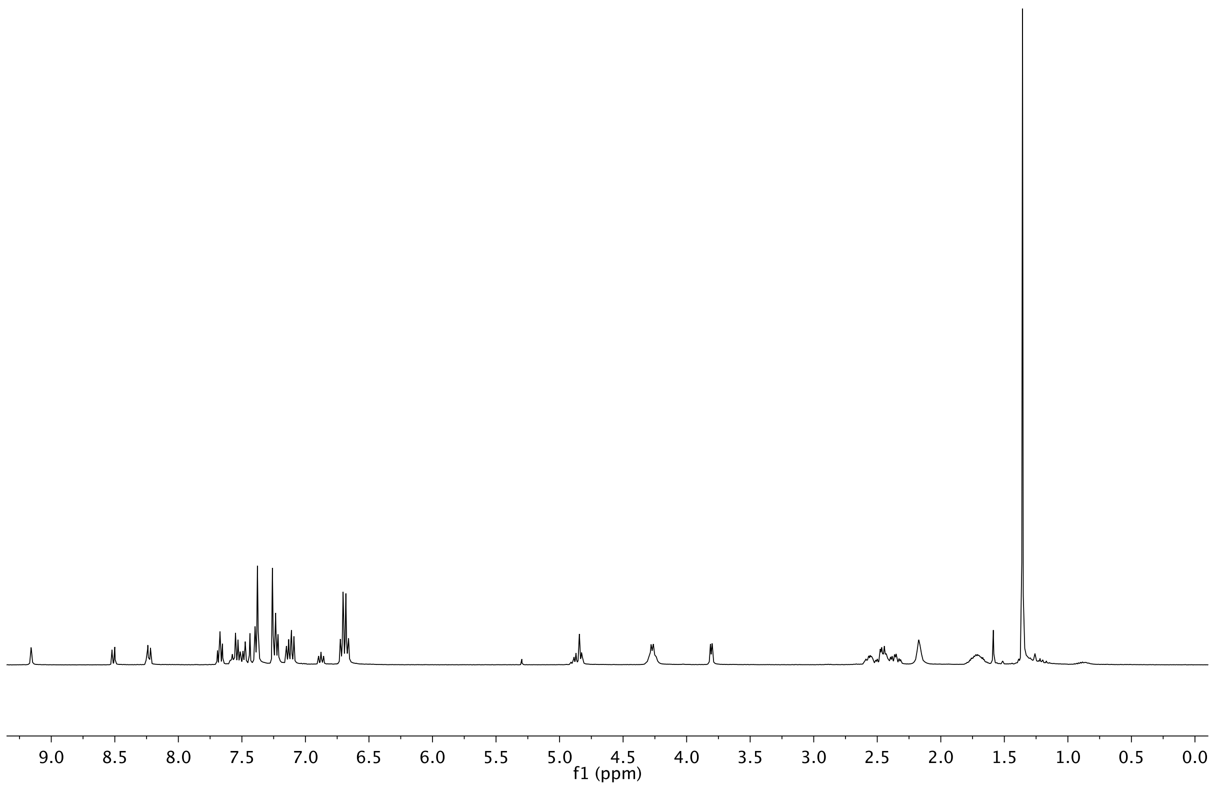

Figure S11 ^{1}H NMR ($CDCl_3$, 400 MHz, 298 K) of **1**.

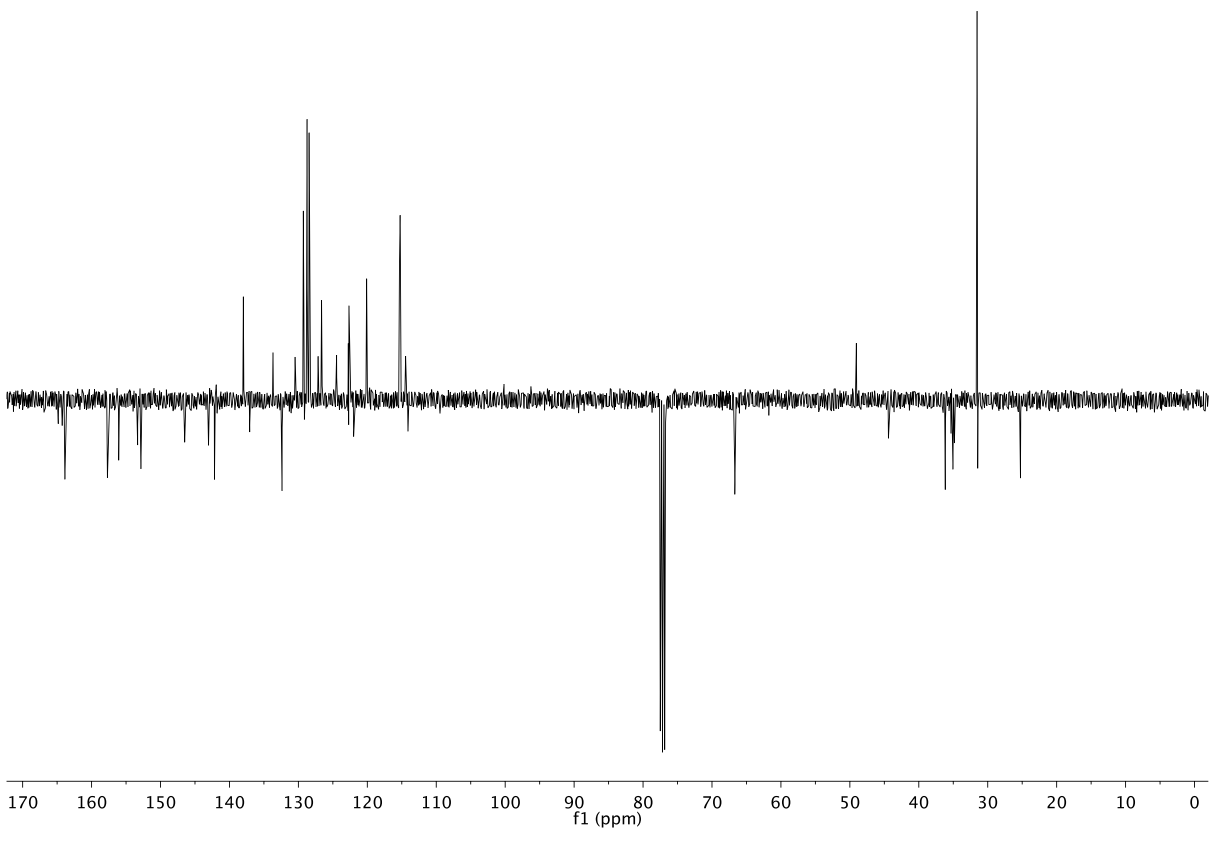

Figure S12 JMOD NMR ($CDCl_3$, 101 MHz, 298 K) of **1**.

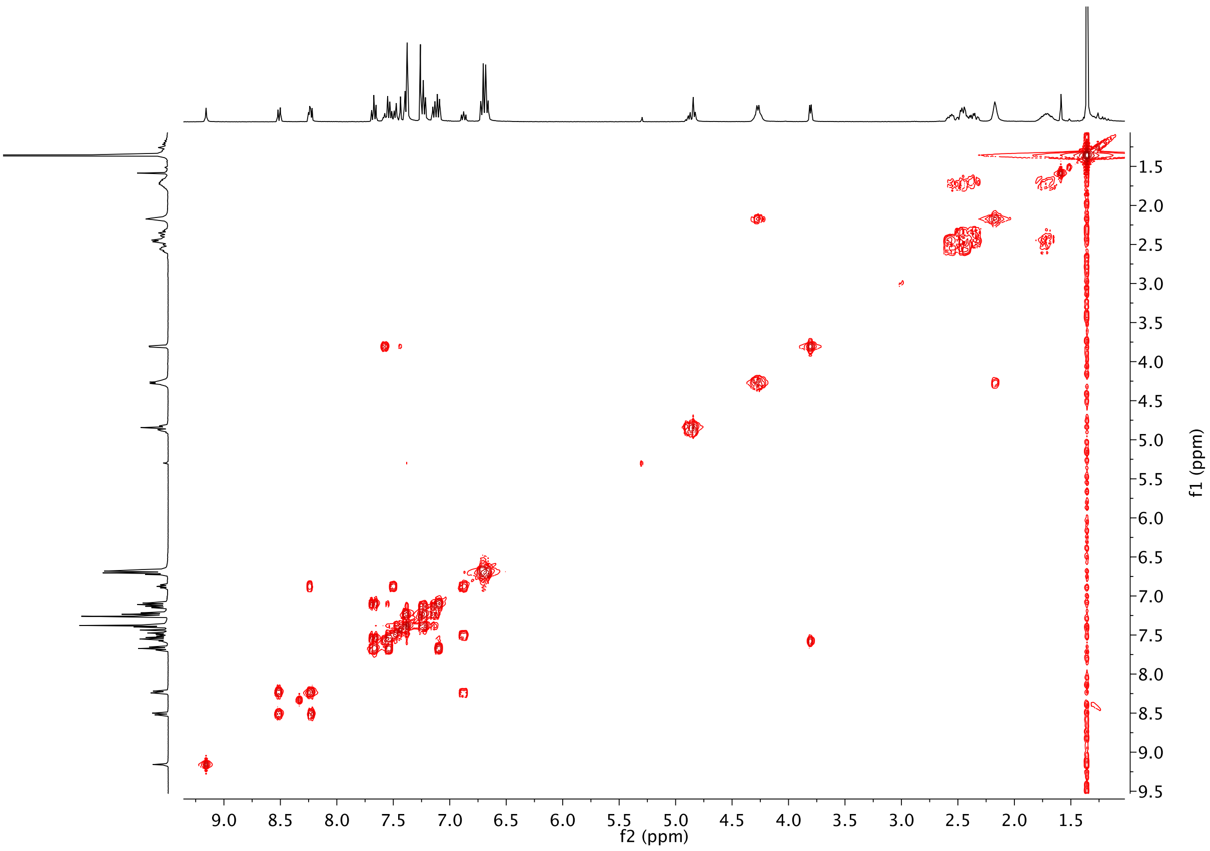

Figure S13 COSY NMR ($CDCl_3$, 400 MHz, 298 K) of **1**.

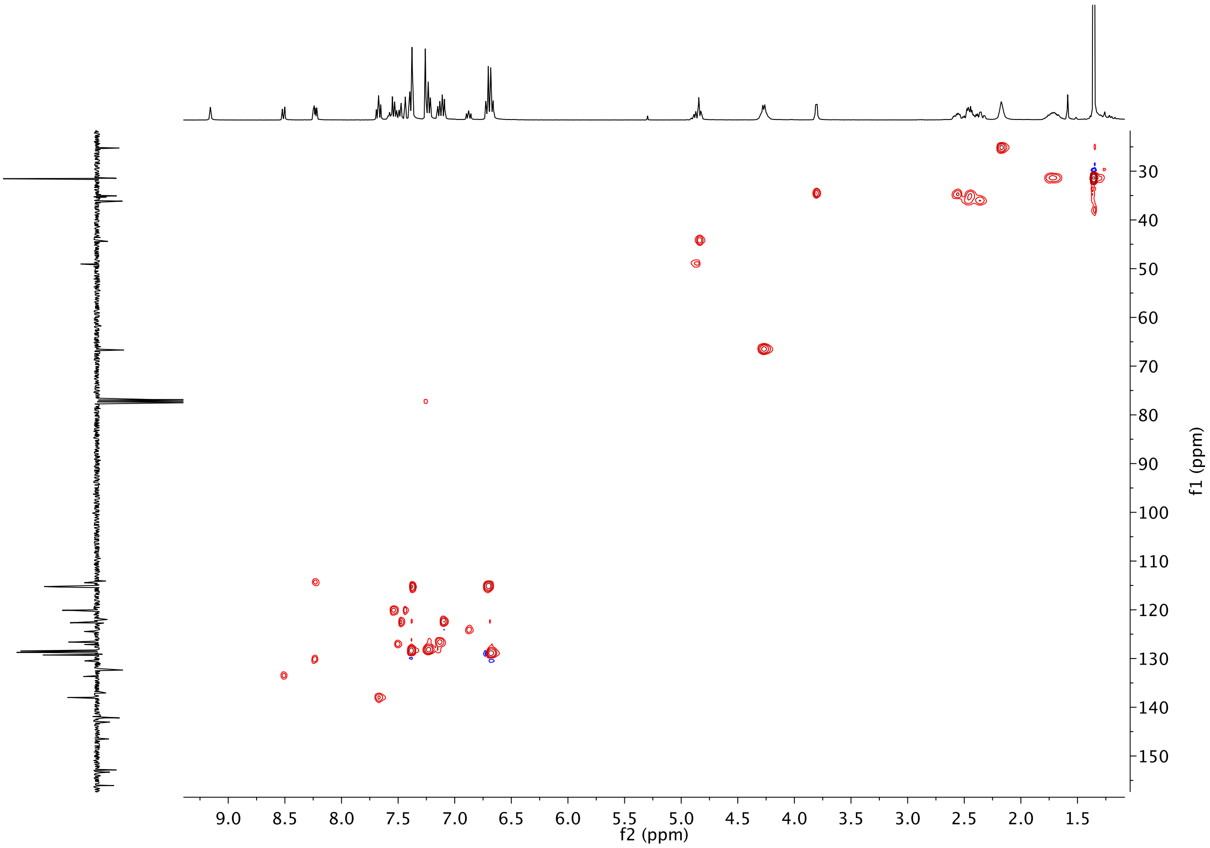

Figure S14 HSQC NMR ($CDCl_3$, 400 MHz, 298 K) of **1**.

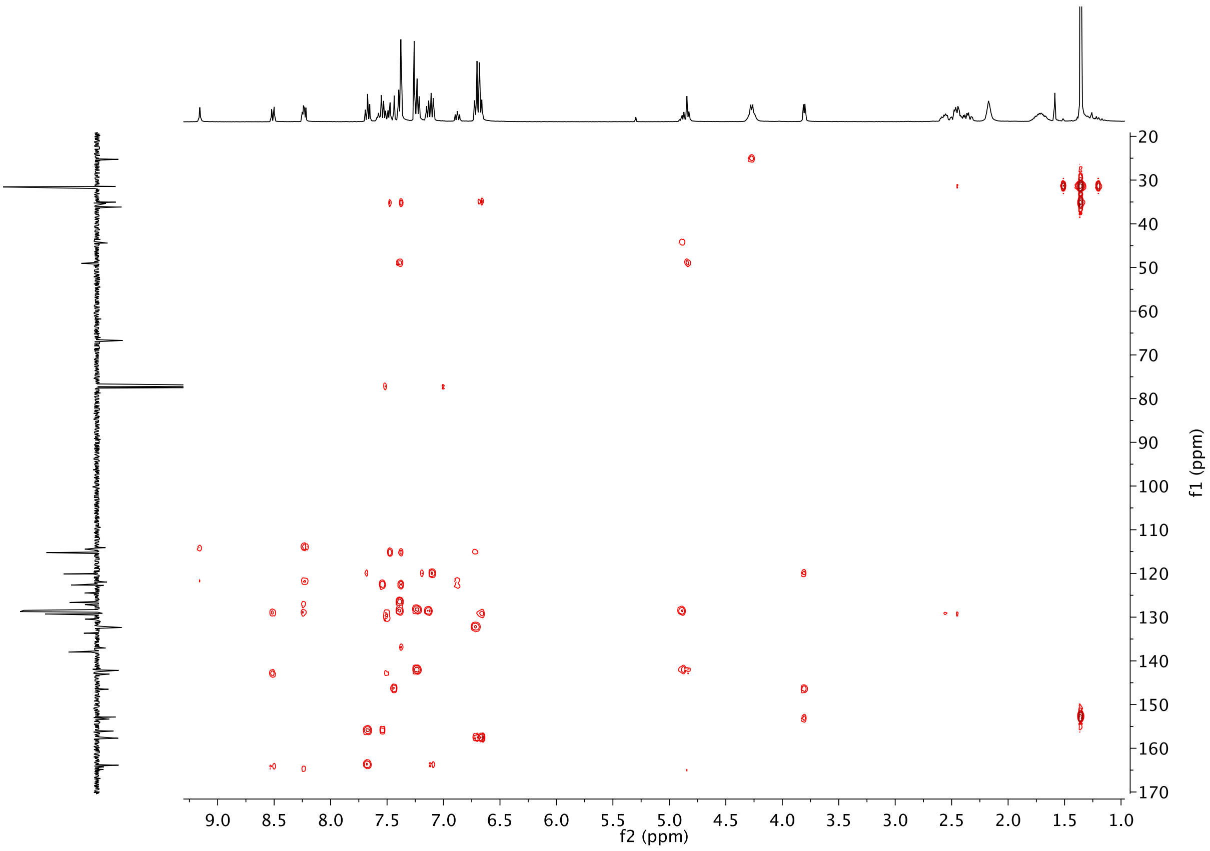

Figure S15 HMBC NMR ($CDCl_3$, 400 MHz, 298 K) of **1**.

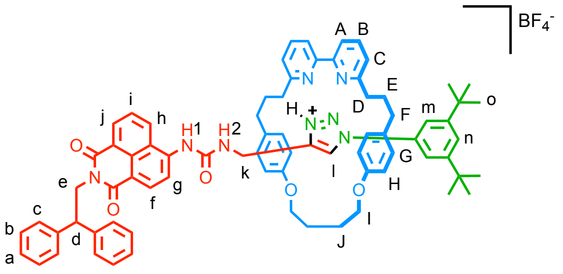

Rotaxane 1.HBF$_4$

A solution of rotaxane **1** (20 mg, 0.017 mmol, 1 equiv.) in CH_2Cl_2 (2 mL) was washed with an aqueous solution of HBF_4 (0.5 M, 2 mL). The pale yellow organic layer was recovered, dried ($MgSO_4$) and the solvent removed under vacuum, affording **1**.HBF$_4$ as a pale yellow solid (21 mg, 96%).^{1}H NMR ($CDCl_3$, 400 MHz, 298 K) δ 8.50 (d, *J* = 7.2, 1H, H$_j$), 8.41 (d, *J* = 8.6, 1H, H$_h$), 8.23 (d, *J* = 8.5, 1H, H$_f$), 8.18 (d, *J* = 8.5, 1H, H$_g$), 8.18 (s, 1H, -*NH1*-), 7.79 (t, *J* = 7.9, 2H, H$_B$), 7.70 (dd, *J* = 8.6, 7.3, 1H, H$_i$), 7.59 (t, *J* = 1.7, 1H, H$_n$), 7.48 (d, *J* = 8.7, 2H, H$_A$), 7.46 (d, *J* = 8.5, 2H, H$_C$), 7.42 – 7.33 (m, 6H, H$_c$ and H$_m$), 7.28 – 7.18 (m, 4H, H$_b$), 7.17 – 7.08 (m, 2H, H$_a$), 6.79 (d, *J* = 8.3, 4H, H$_G$), 6.64 (d, *J* = 8.3, 4H, H$_H$), 6.59 (s, 1H, -*NH2*-), 6.42 (s, 1H, H$_l$), 4.92 – 4.79 (m, 3H, H$_d$ and H$_e$), 4.34 – 4.10 (m, 4H, H$_I$), 4.06 (d, *J* = 6.1, 2H, H$_k$), 2.76 – 2.65 (m, 2H, H$_F$), 2.65 – 2.55 (m, 4H, H$_D$), 2.53 – 2.41 (m, 2H, H$_F$), 2.26 (m, 2H, H$_J$), 2.10 – 2.00 (m, 2H, H$_J$), 2.00 – 1.90 (m, 2H, H$_E$), 1.90 – 1.76 (m, 2H, H$_E$), 1.47 (s, 18H, H$_o$). ^{13}C NMR ($CDCl_3$, 101 MHz, 298 K) δ 164.7, 164.2, 162.6, 157.4, 154.9, 152.9, 148.3, 144.0, 142.3, 142.1, 141.3, 136.7, 132.6, 132.4, 131.4, 129.8, 129.0, 128.7, 128.4, 127.9, 126.7, 126.6, 126.0, 123.3, 122.5, 120.7,

120.6, 116.0, 115.3, 114.6, 114.1, 66.5, 48.9, 44.4, 34.9, 34.9, 34.3, 34.1, 31.7, 30.2, 25.0. ^{19}F NMR ($CDCl_3$, 376 MHz, 298 K) δ -149.9, -150.0.

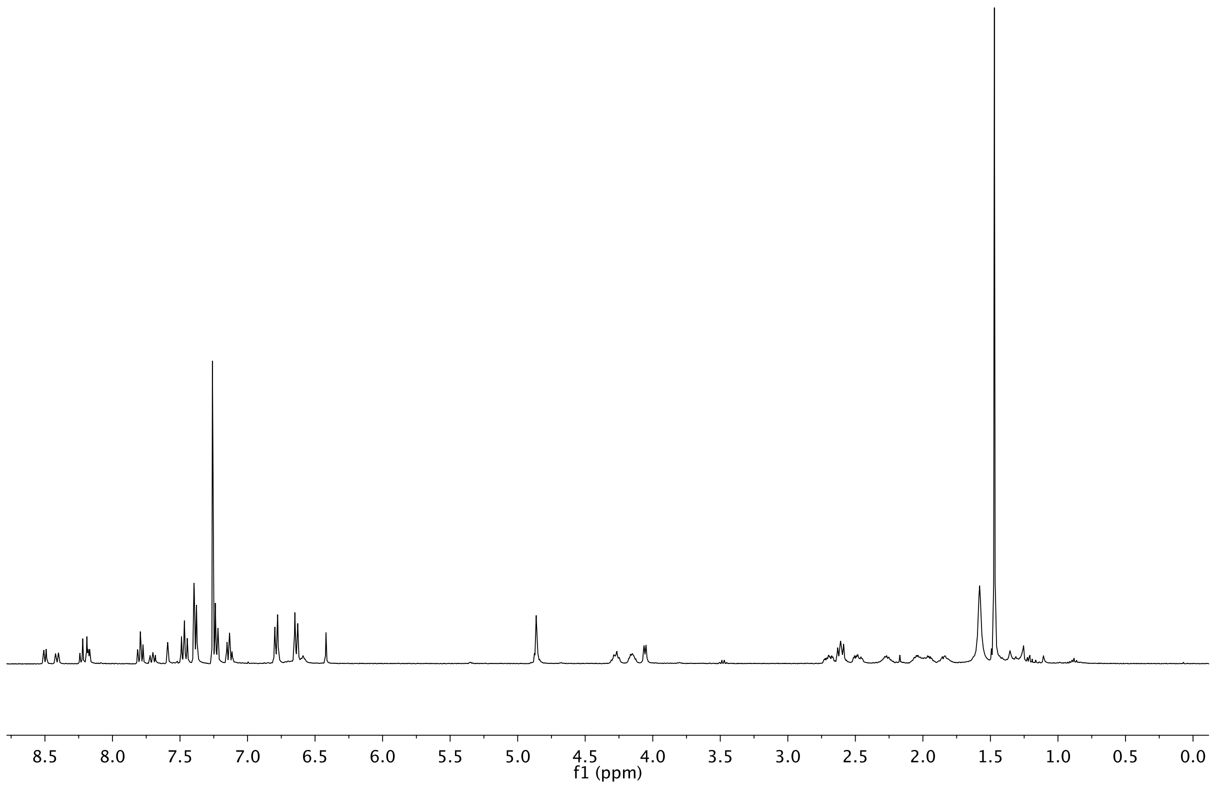

Figure S16 ^1H NMR ($CDCl_3$, 400 MHz, 298 K) of **1**.HBF_4.

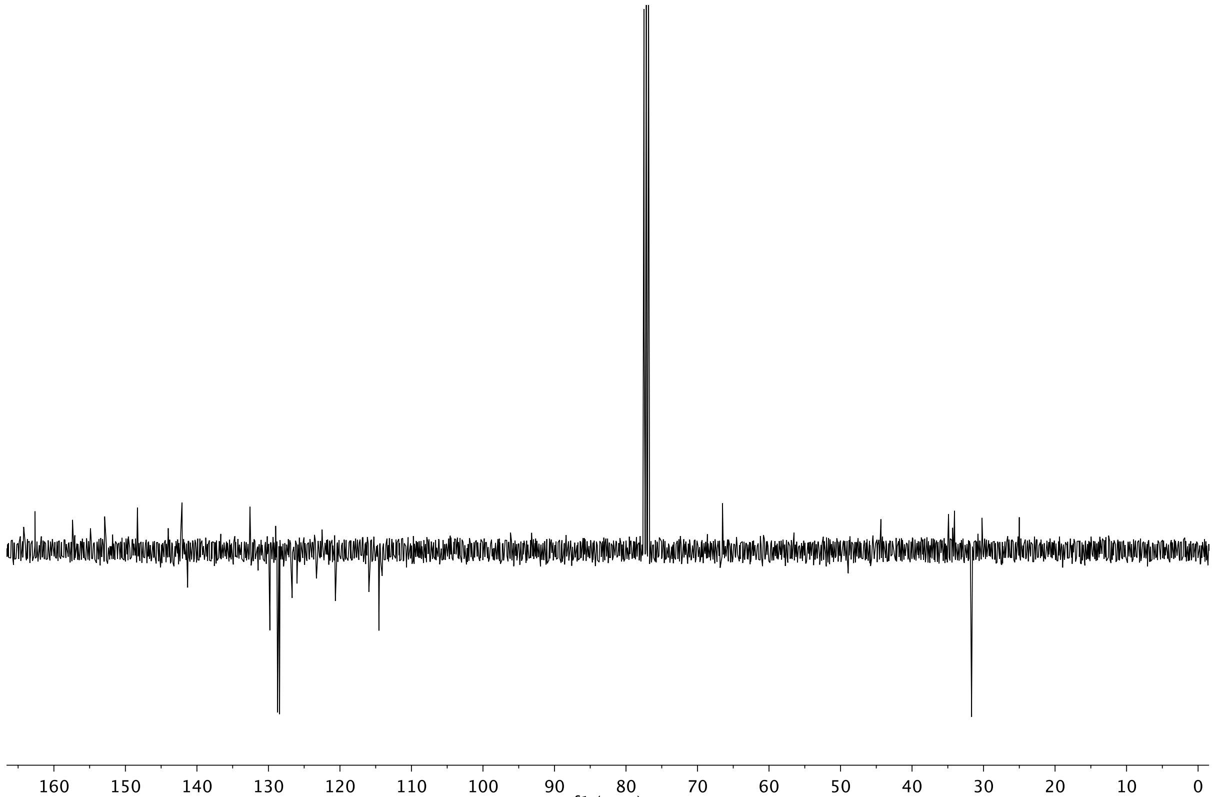

Figure S17 JMOD NMR ($CDCl_3$, 101 MHz, 298 K) of **1**.HBF_4.

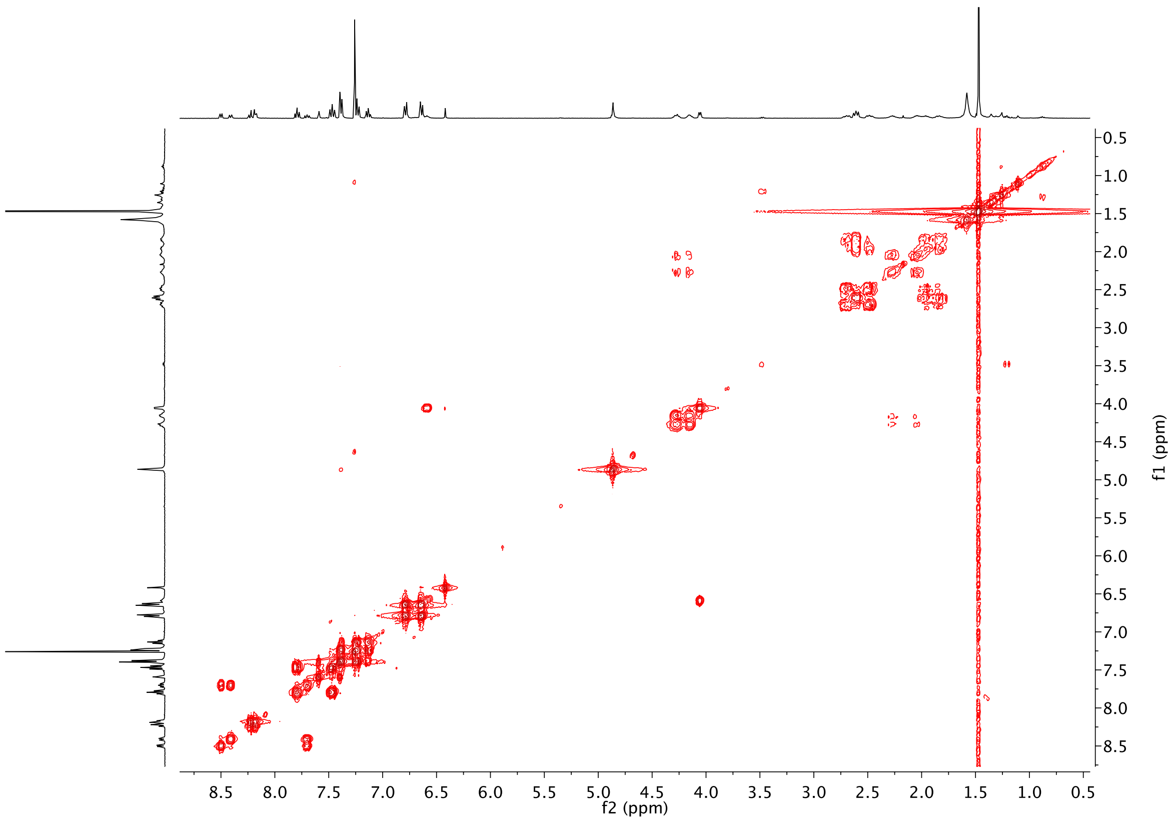

Figure S18 COSY NMR ($CDCl_3$, 400 MHz, 298 K) of **1**.HBF_4.

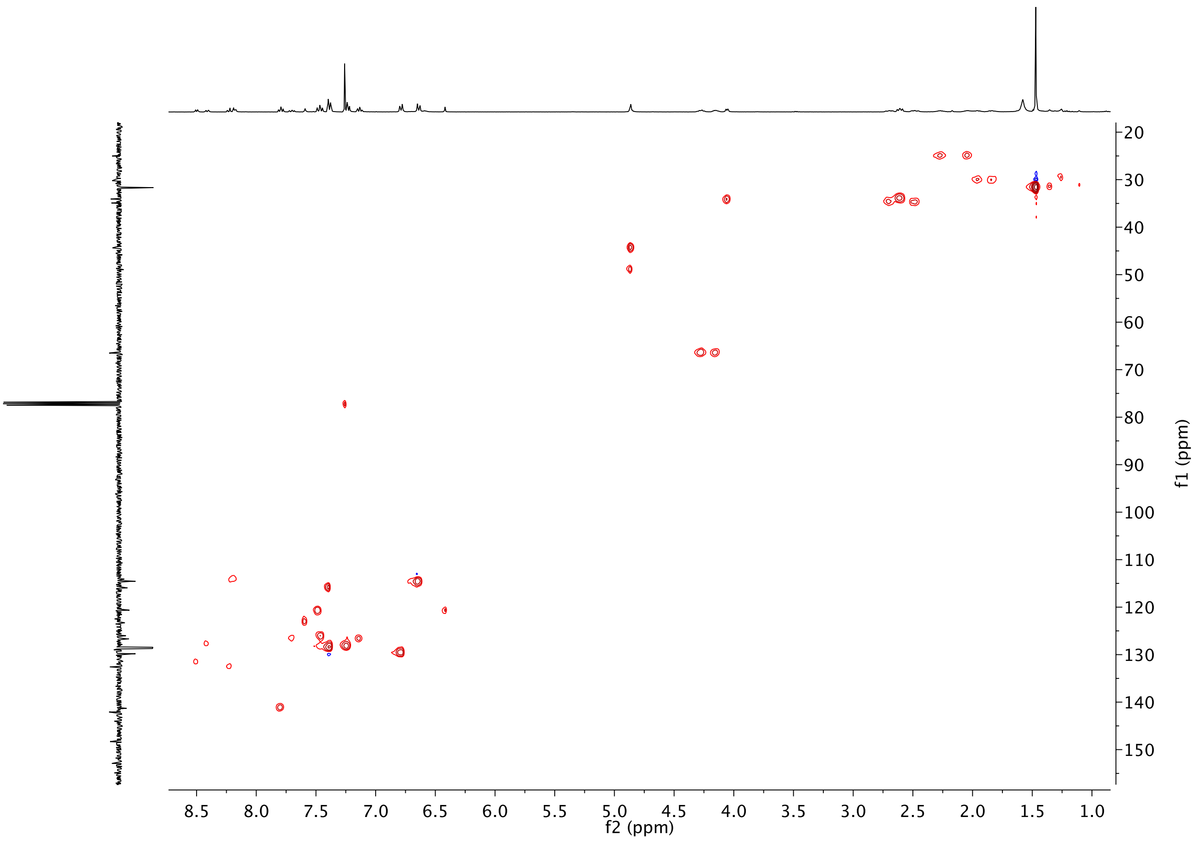

Figure S19 HSQC NMR ($CDCl_3$, 400 MHz, 298 K) of **1**.HBF_4.

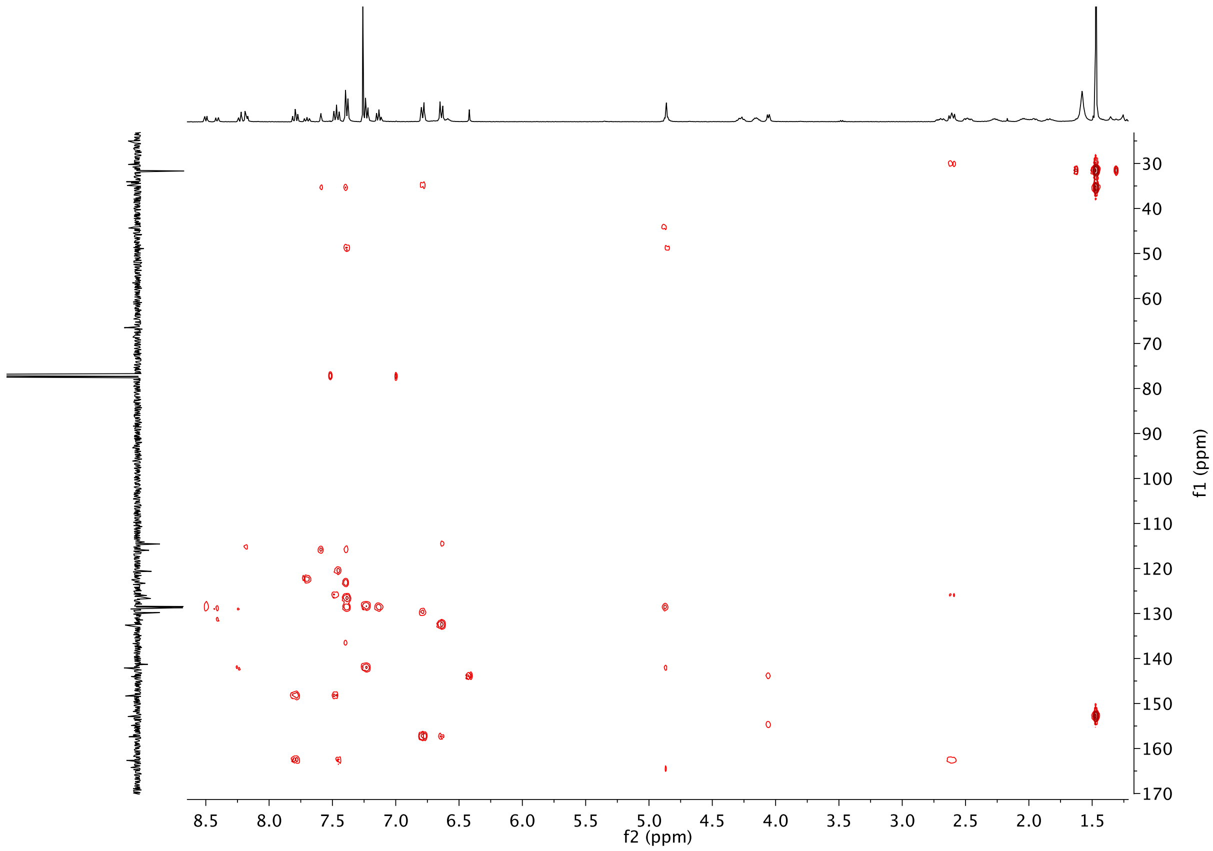

Figure S20 HMBC NMR ($CDCl_3$, 400 MHz, 298 K) of **1**.HBF_4.

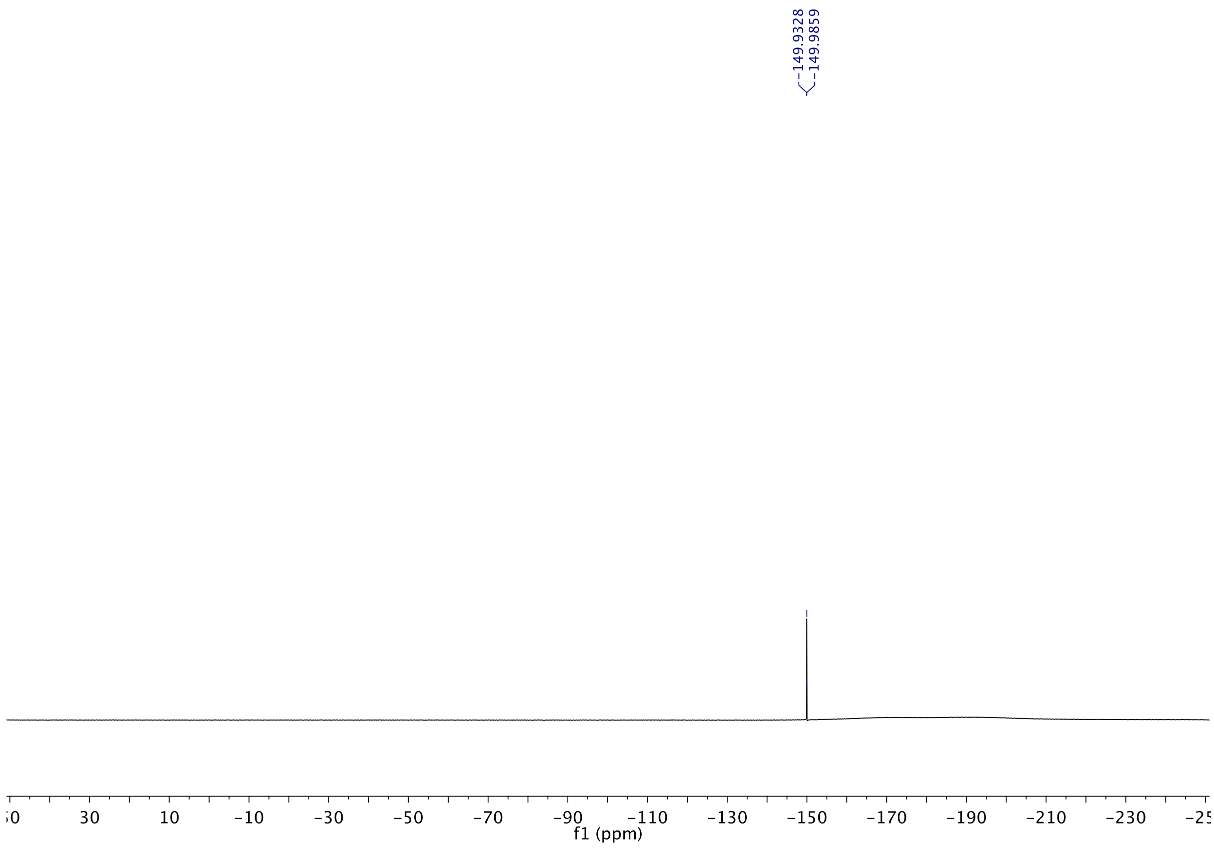

Figure S21 ^{19}F NMR ($CDCl_3$, 376 MHz, 298 K) of **1**.HBF_4.

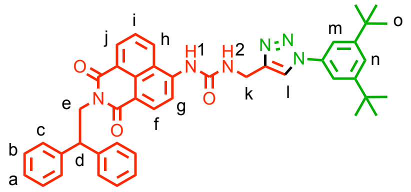

Axle 2

A dry CEM MW vial was charged with azide **S1** (5.8 mg, 0.025 mmol, 1 equiv.), alkyne **S5** (12 mg, 0.025 mmol, 1 equiv.), and $[Cu(MeCN)_4]PF_6$ (8.9 mg, 0.024 mmol, 0.96 equiv.). CH_2Cl_2 (1 mL) was added, followed by DIPEA (4.4 µL, 0.025 mmol, 1 equiv.) and the reaction mixture stirred at 50 ºC for 4 h. EDTA-NH_3 (aq.) (5 mL) was added and the crude extracted with CH_2Cl_2 (3 x 5 mL). The combined organic layers were dried ($MgSO_4$) and concentrated *in vacuo*. The crude was purified *via* flash column chromatography on silica gel using an isocratic elution of CH_2Cl_2/MeOH 95:5, affording product **2** as a pale yellow solid (17 mg, 97%).^{1}H NMR ($CDCl_3$, 400 MHz, $CDCl_3$, 298 K) δ 8.58 (s, 1H, -*NH1*-), 8.44 (d, *J* = 8.3, 1H, H_f), 8.38 (d, *J* = 8.3, 1H, H_g), 8.28 (d, *J* = 7.4, 1H, H_j), 8.24 (d, *J* = 8.5, 1H, H_h), 8.17 (s, 1H, H_l), 7.51 (t, *J* = 1.7, 1H, H_n), 7.39 (d, *J* = 1.7, 2H, H_m), 7.33 (dd, *J* = 8.2, 1.3, 4H, H_c), 7.21 (t app, *J* = 7.5, 4H, H_b), 7.12 (dd, *J* = 7.5, 1.3 Hz, 2H, H_a), 6.98 (t app, *J* = 7.9, 1H, H_i), 4.86 – 4.72 (m, 5H, H_d H_e and H_k), 1.24 (s, 18H, H_o). ^{13}C NMR ($CDCl_3$, 101 MHz, 298 K) δ 164.4, 163.9, 153.4, 141.9, 141.2, 132.8, 130.9, 129.1, 128.6, 128.4, 127.3, 126.7, 125.7, 124.0, 123.3, 122.9, 117.2, 116.5, 116.1, 48.8, 44.5, 35.3, 31.3. HRMS m/z = 705.3556 $[M+H]^+$ (calc. for $C_{44}H_{45}N_6O_3$ 705.3548).

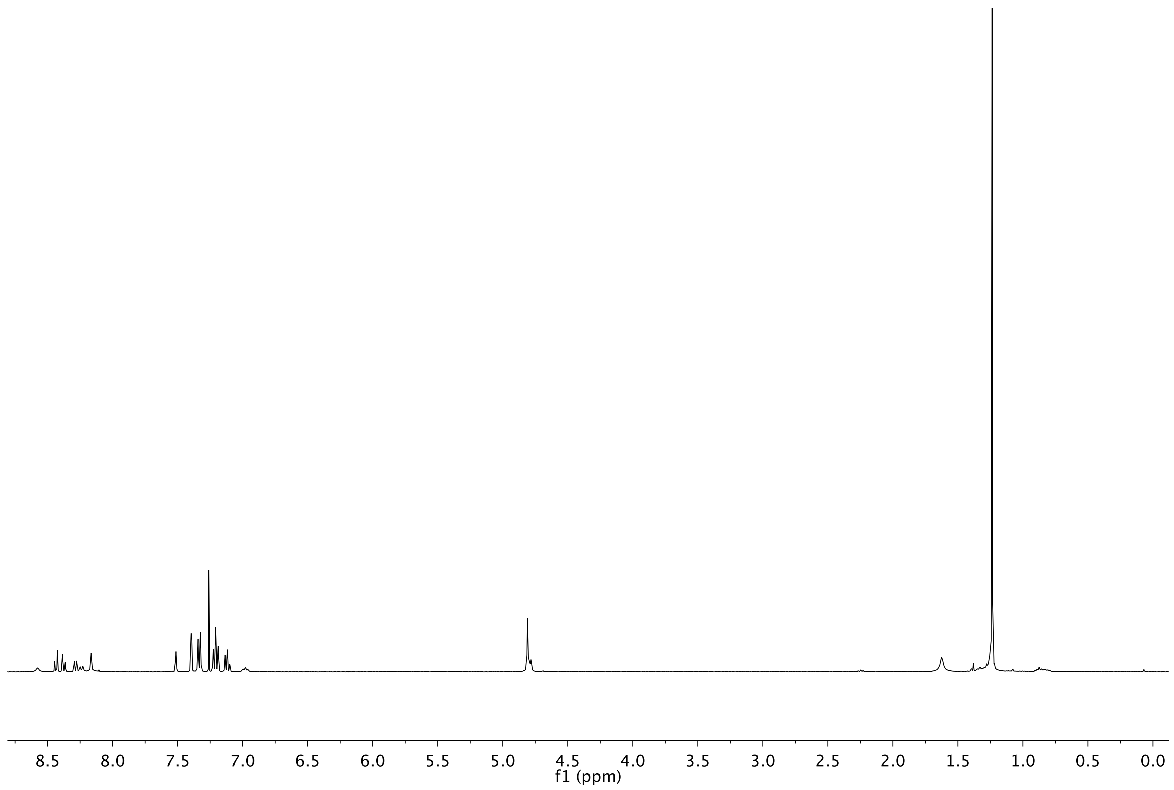

Figure S22 ^{1}H NMR ($CDCl_3$, 400 MHz, 298 K) of **2**.

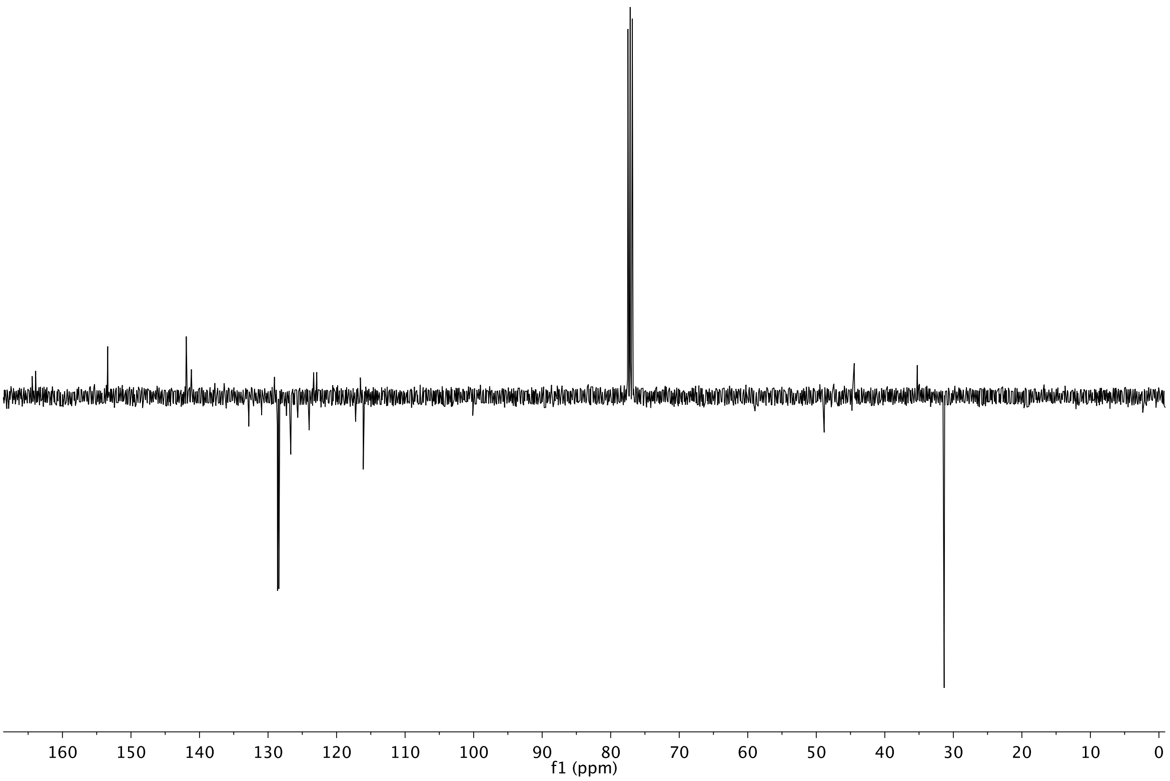

Figure S23 JMOD NMR ($CDCl_3$, 101 MHz, 298 K) of **2**.

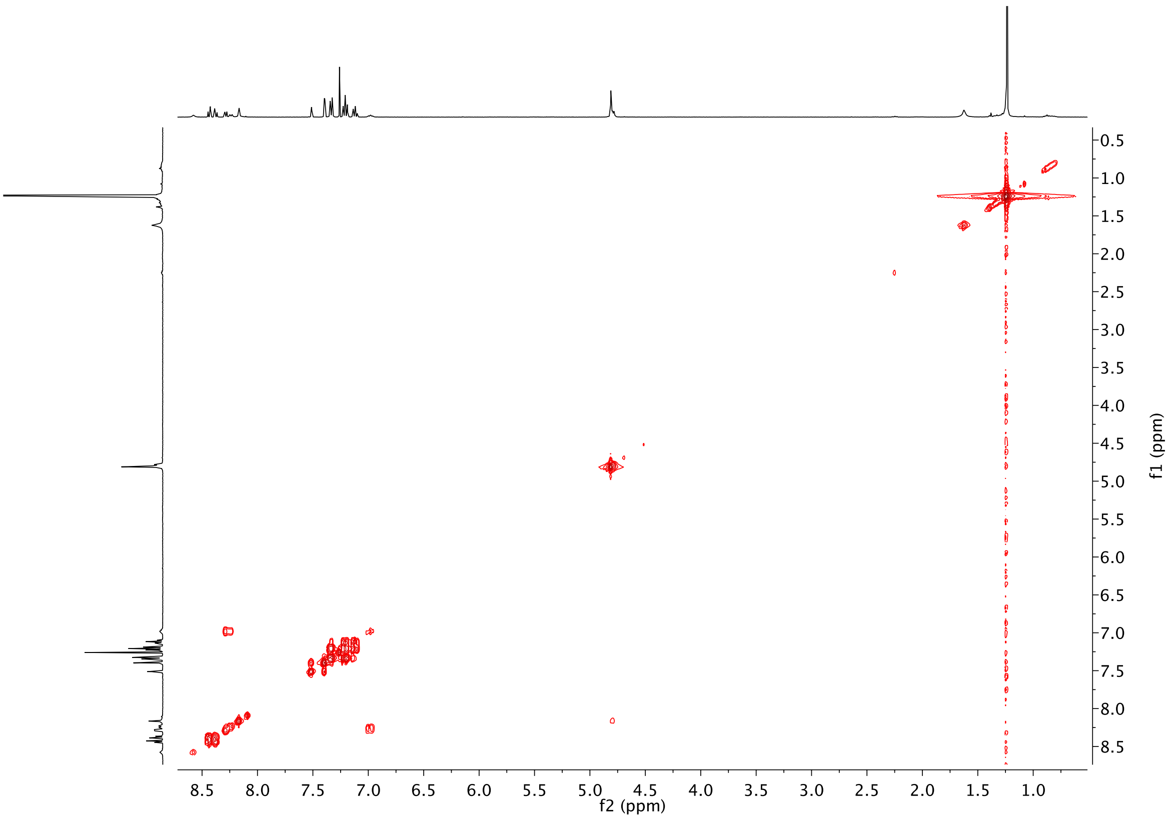

Figure S24 COSY NMR ($CDCl_3$, 400 MHz, 298 K) of **2**.

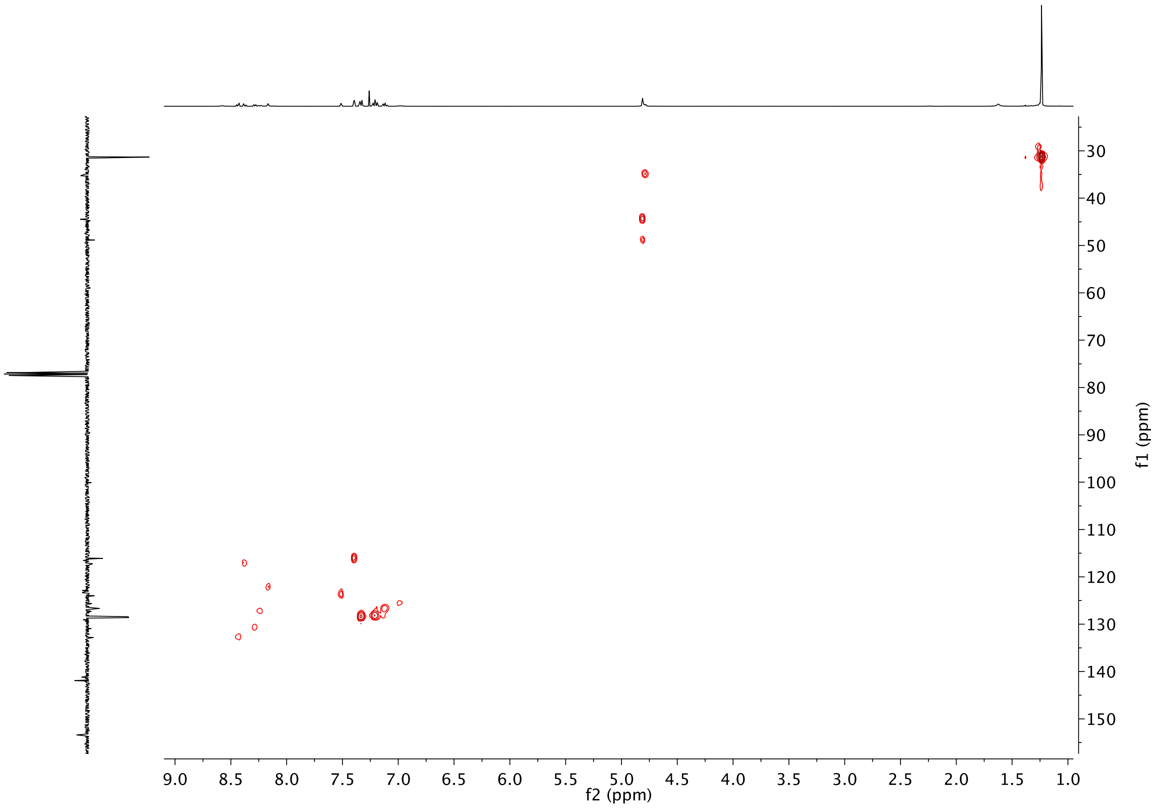

Figure S25 HSQC NMR ($CDCl_3$, 400 MHz, 298 K) of **2**.

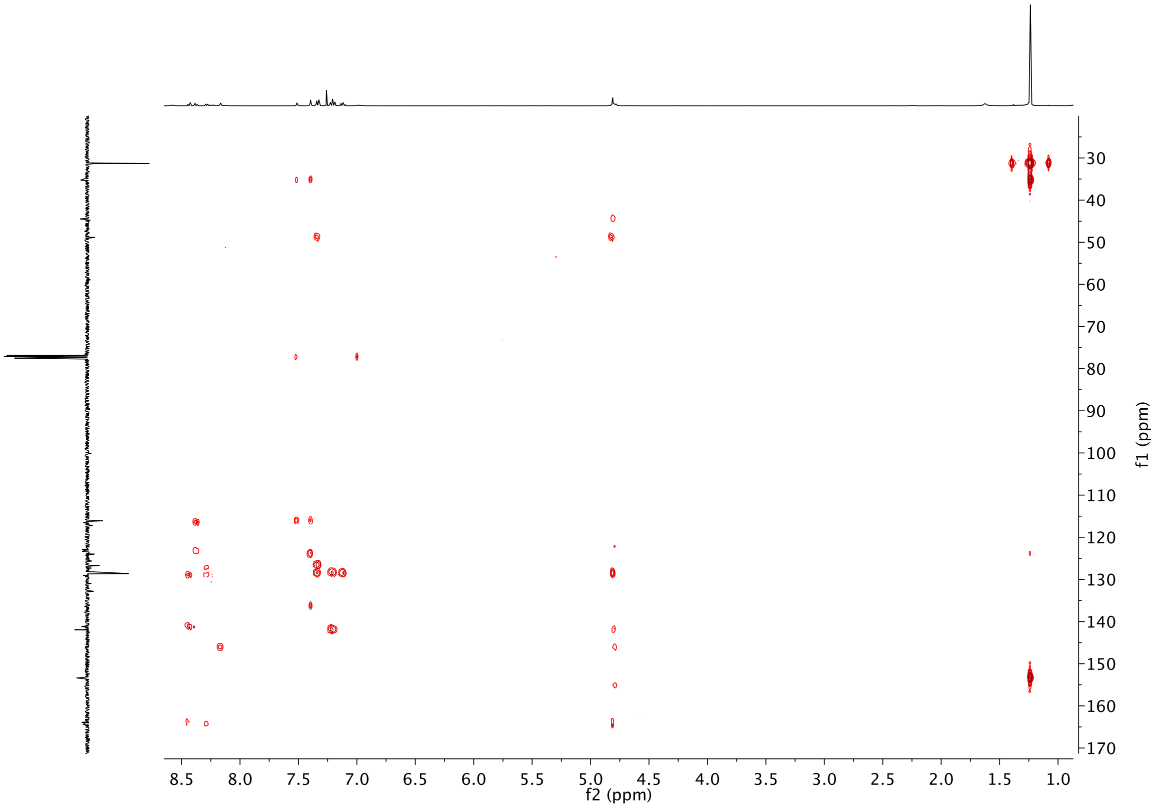

Figure S26 HMBC NMR ($CDCl_3$, 400 MHz, 298 K) of **2**.

NMR and fluorescence titration data

^{1}H NMR Binding Studies Procedure: A 2.5 mM stock solution of the receptor was accurately prepared in the stated deuterated solvents using a volumetric flask. Solutions of anions (as their tetrabutylammonium salts) to be titrated were then prepared in separate vials using the same host solution so that the concentration of the host remained constant throughout given titration experiment. The concentration of anion solutions was made 70 times that of the host (i.e. 160 – 180 mM). In each case, 550 µL of host solution in an NMR tube was titrated with aliquots of anion stock solution, and after each addition, the ^{1}H NMR spectrum was recorded on a Bruker Avance III 400 or Bruker Avance III 500 spectrometer after thorough mixing in 298K. Typically, this was performed in the following order: 10 × 1.5 µL, 2×7.5 µL, 4× 14 µL (total 86 µL). Titrations were performed in triplicate to give K_a values. Typically, a total of at least 12 equiv. of anion was added. Non-linear curve fitting of the experimentally obtained titration isotherms (equivalents of anion versus chemical shift of NH and triazole proton) using the program HypNMR® (Hyperquad®) enabled the calculation of association constants (K_a/M^{-1}) using a 1:1 global fitting model.

Spectroscopic Binding Studies Procedure: A 0.13 mM stock solution of the receptor was accurately prepared in the spectrophotometric solvents using a volumetric flask. Solutions of anions (as their tetrabutylammonium salts) to be titrated were then prepared in separate vials using the same host solution so that the concentration of the host remained constant throughout given titration experiment. The concentration of anion solutions was made 70 times that of the host (i.e. 8 – 10 mM). Typically, a total of at least 12 equiv. of anion was added, and this was performed in the following order: 10 × 1.5 µL, 2×7.5 µL, 4× 14 µL (total 86 µL). After each addition, the resulting solution was stirred for at least 20 seconds, and the absorbance was recorded. Both salt and receptor were dried under high vacuum prior to use. UV-Vis data was recorded using a Varian Cary 4000 UV-Vis Spectrophotometer. Temperature control was provided by a Varian Cary PCB 150 Water Peltier System. The absorbance was recorded from 250 nm to 600 nm. Titrations were performed in triplicate to give K_a values. To determine association constants for the receptor-indicator complexes, global analysis of the absorbance data was carried out using a nonlinear least-squares curve fitting procedure using the online software http://supramolecular.org/ with a 1:1 global fitting model (Nelder-Mead method). Fluorescence titrations were carried out in parallel to the UV/Vis absorption measurements using a Cary Eclipse Fluorescence Spectrometer.

^{1}H NMR titrations of axle 2

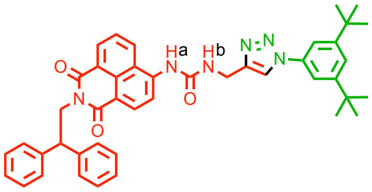

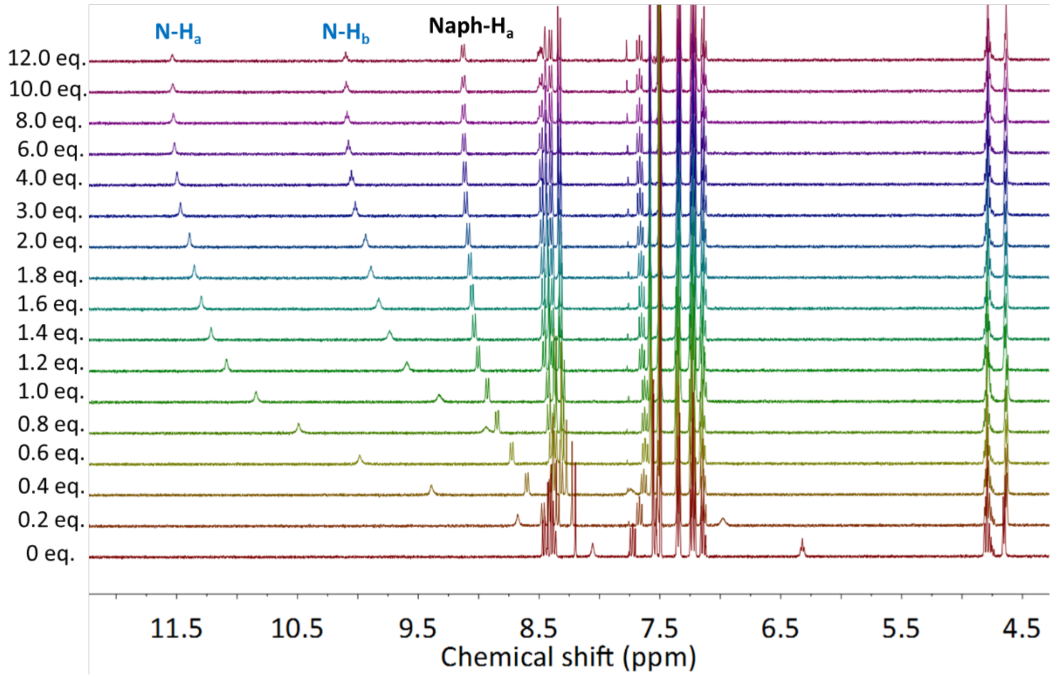

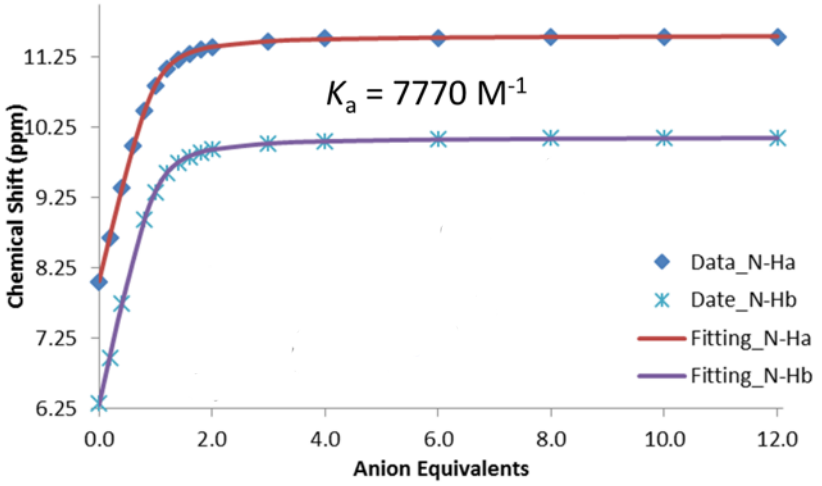

Figure S27 ^{1}H NMR titration of **2** with TBAAcO (0 - 12 equiv.) in $CDCl_3/CD_3CN$ at 298 K.

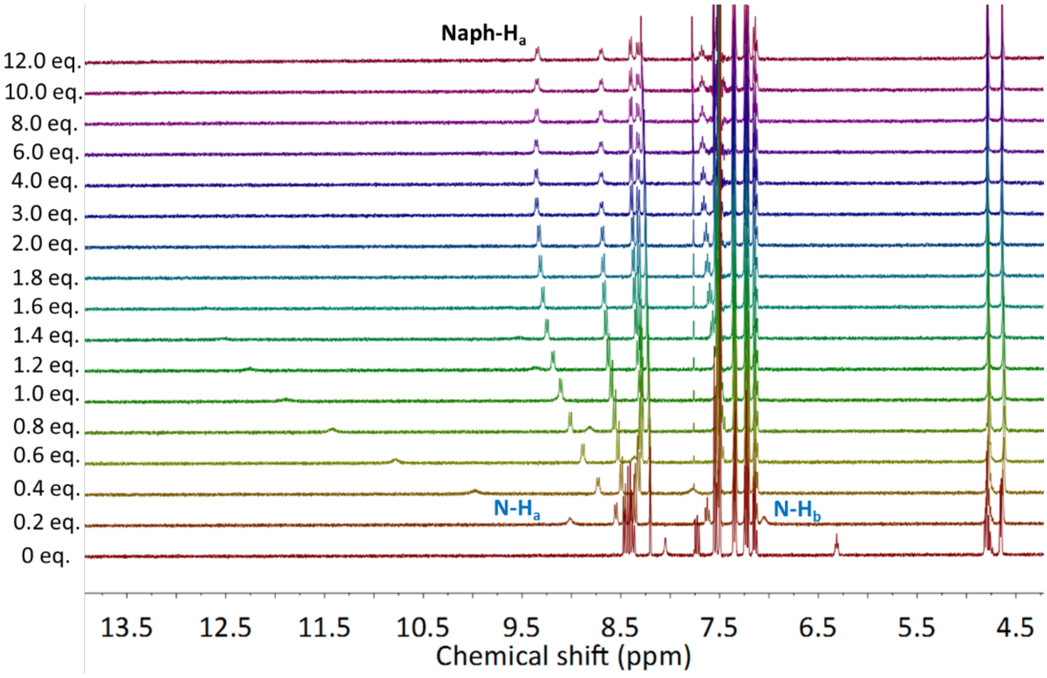

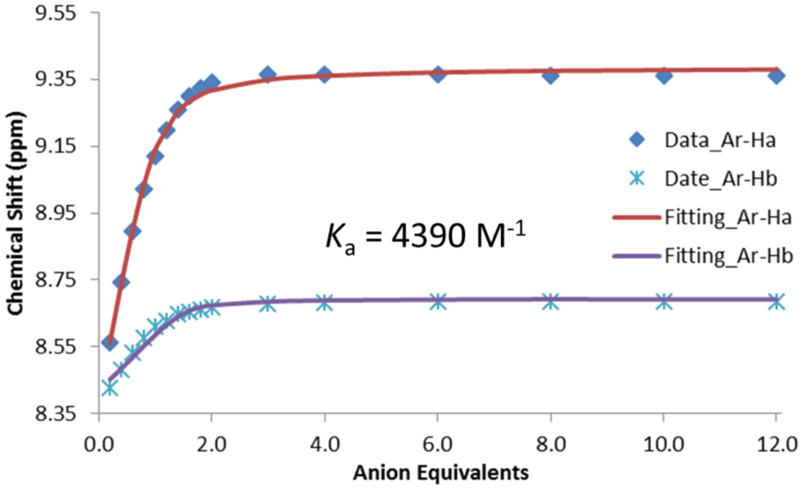

Figure S28 ^{1}H NMR titration of **2** with TBAF (0 - 12 equiv.) in $CDCl_3/CD_3CN$ at 298 K.

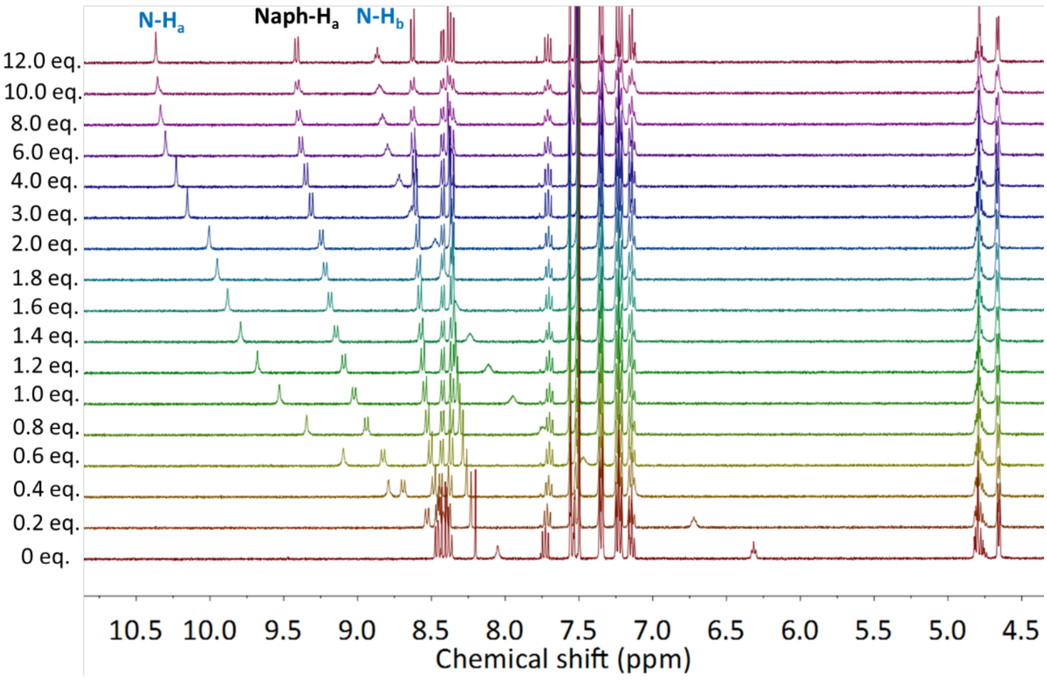

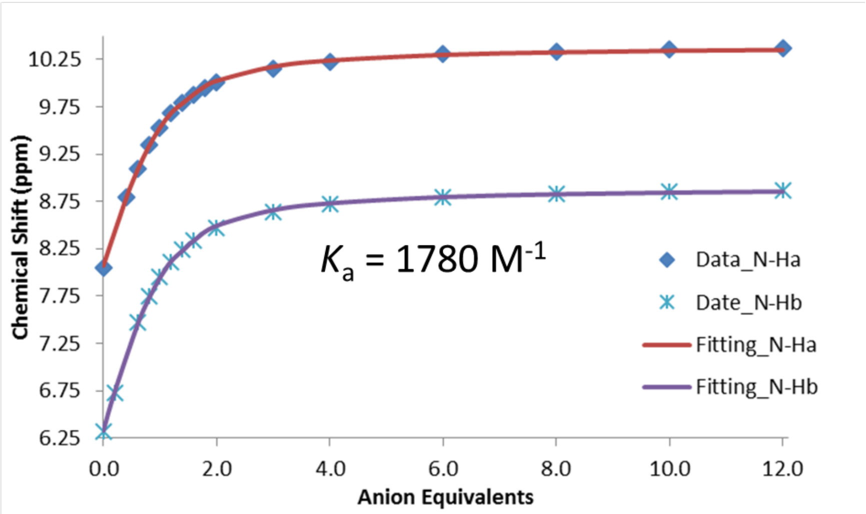

Figure S29 ^{1}H NMR titration of **2** with TBACl (0 - 12 equiv.) in $CDCl_3/CD_3CN$ at 298 K.

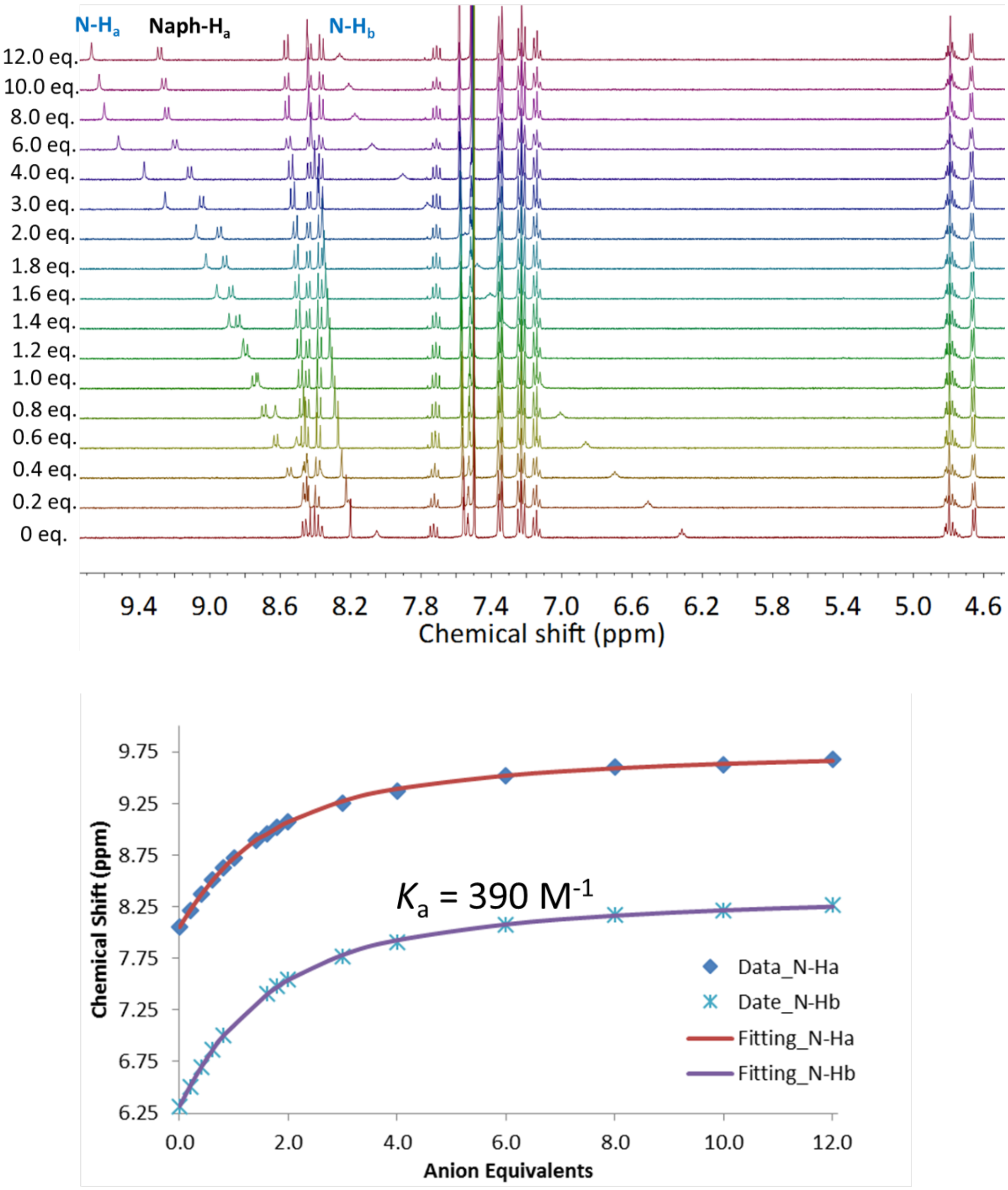

Figure S30 ^{1}H NMR titration of **2** with TBABr (0 - 12 equiv.) in $CDCl_3$/CD_3CN at 298 K.

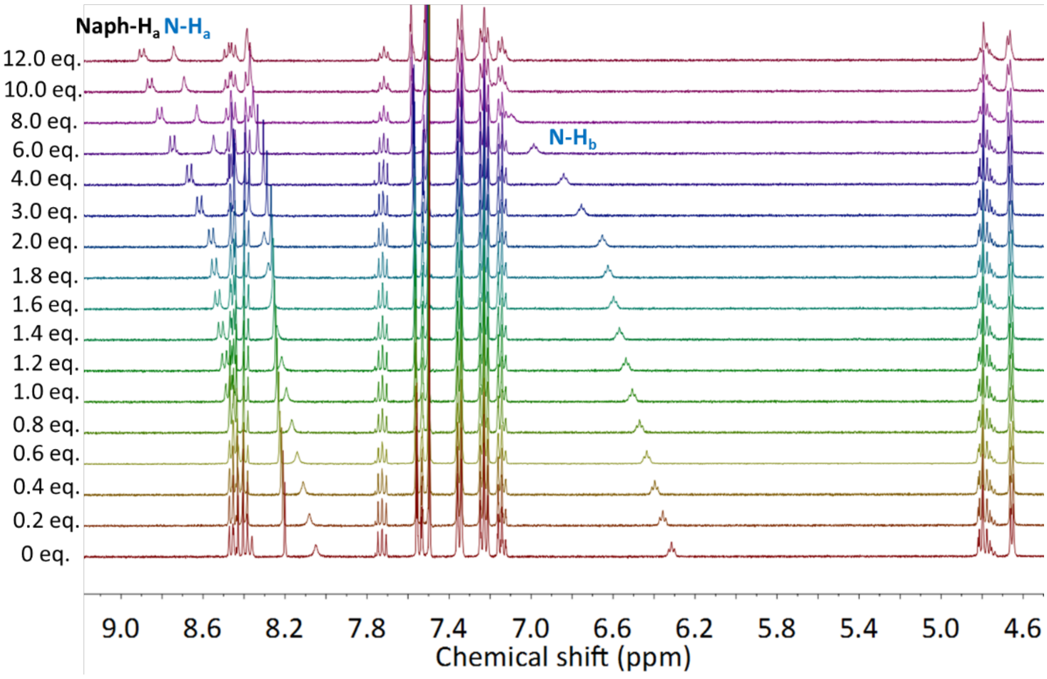

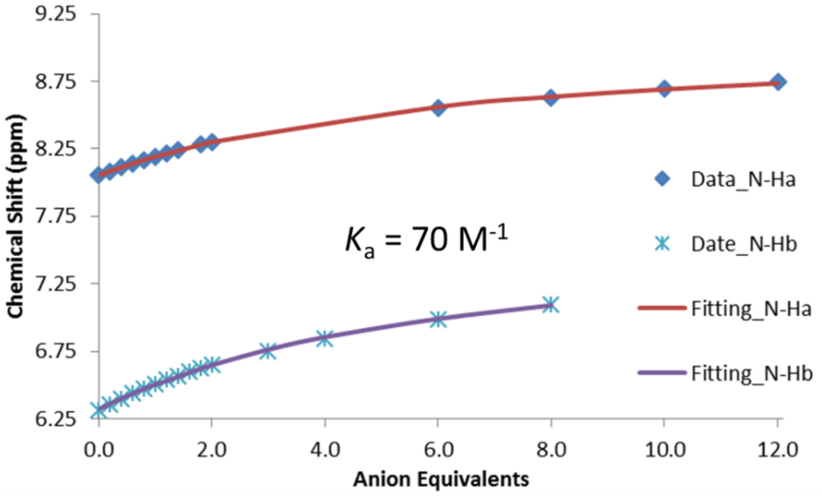

Figure S31 ^{1}H NMR titration of **2** with TBAI (0 - 12 equiv.) in $CDCl_3/CD_3CN$ at 298 K.

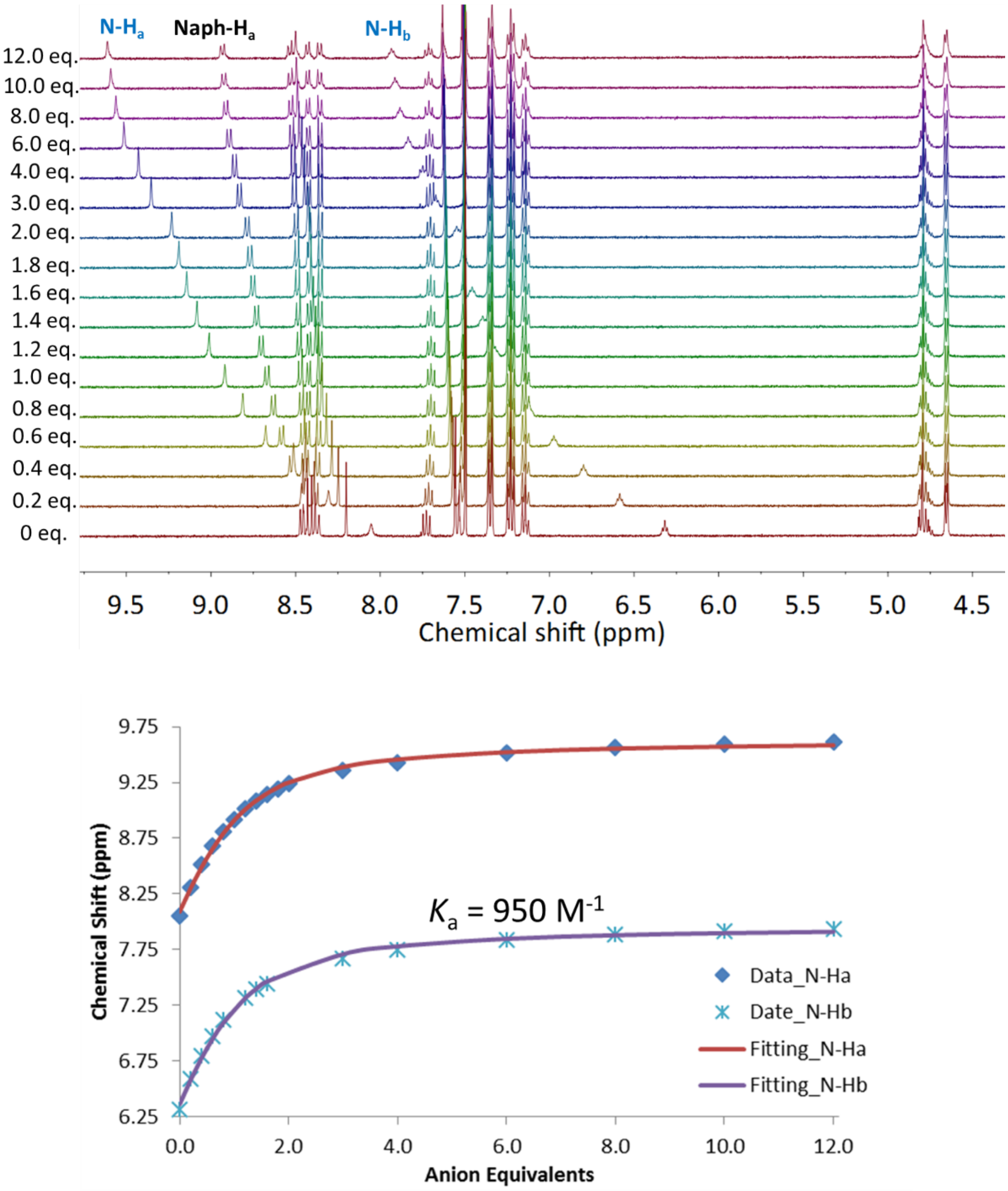

Figure S32 ^{1}H NMR titration of **2** with TBAMsO (0 - 12 equiv.) in $CDCl_3/CD_3CN$ at 298 K.

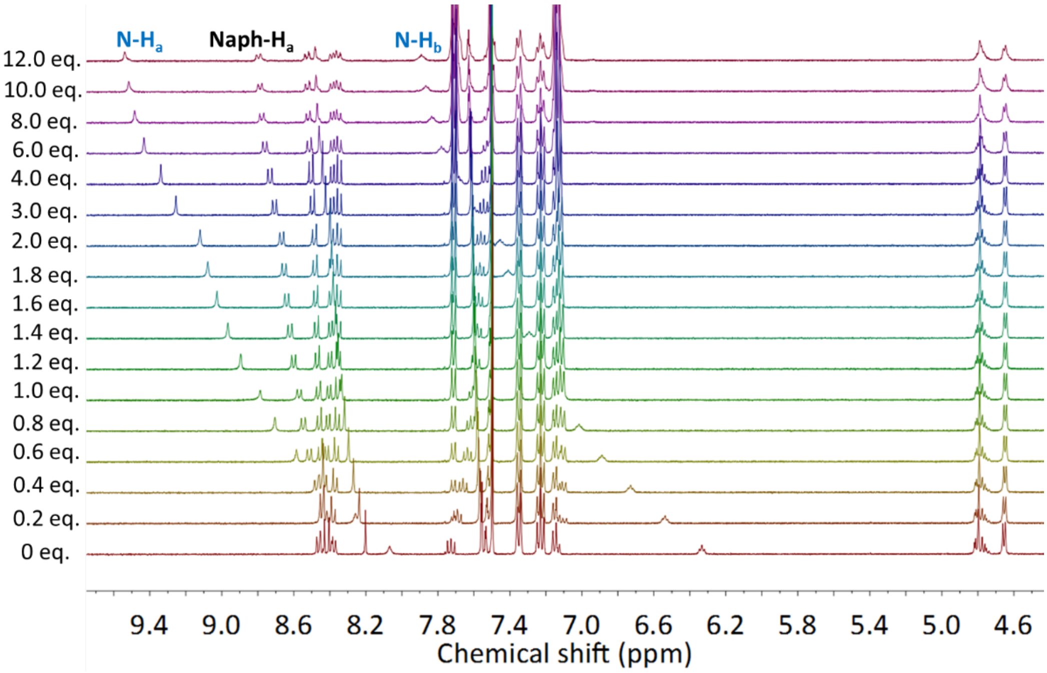

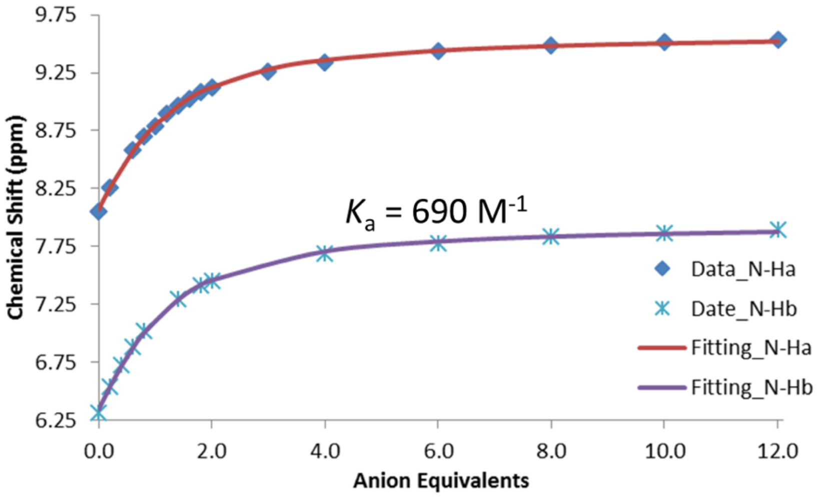

Figure S33 ^{1}H NMR titration of **2** with TBATsO (0 - 12 equiv.) in $CDCl_3/CD_3CN$ at 298 K.

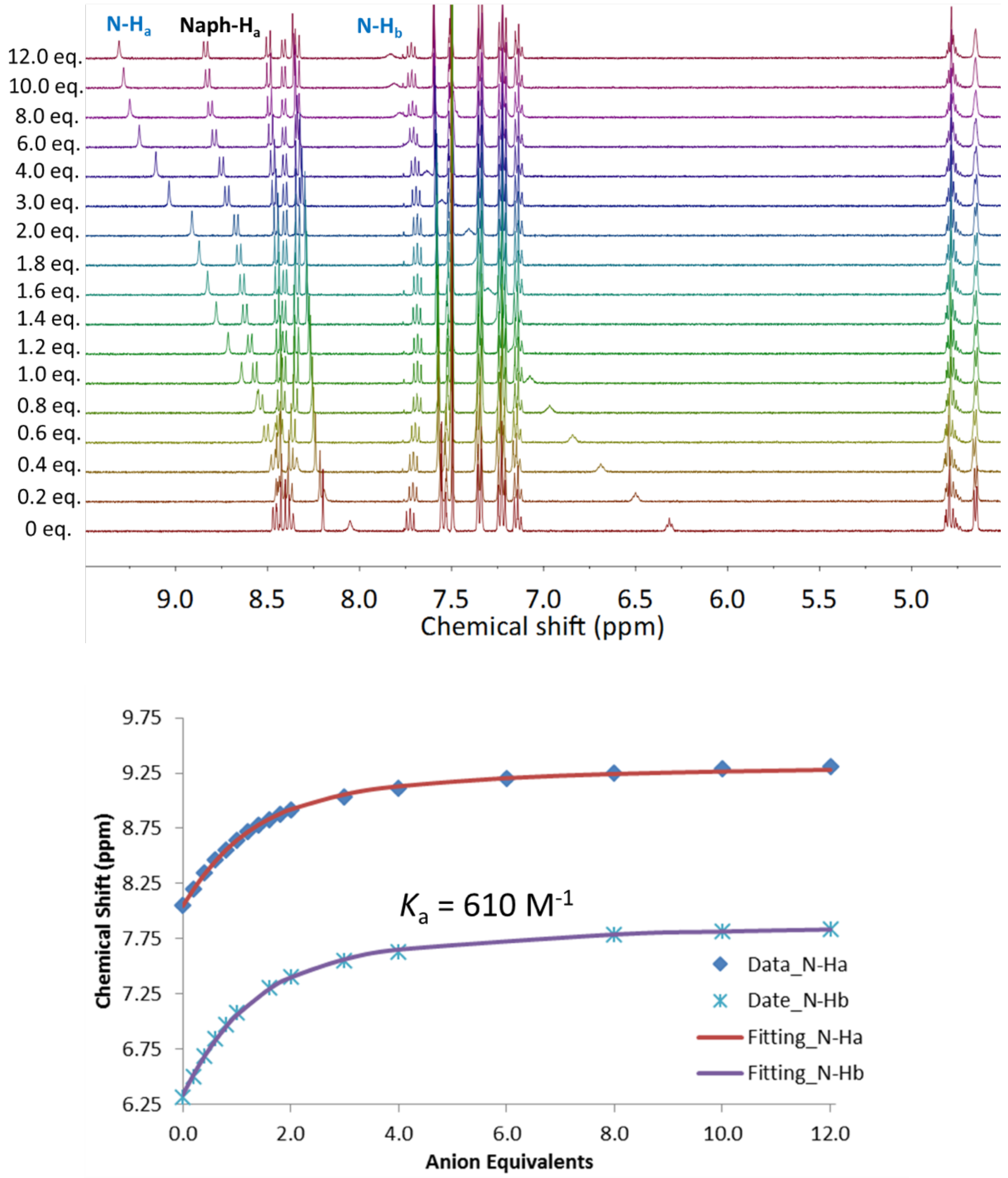

Figure S34 ^{1}H NMR titration of **2** with $TBAHSO_4$ (0 - 12 equiv.) in $CDCl_3/CD_3CN$ at 298 K.

UV-vis titrations of axle 2

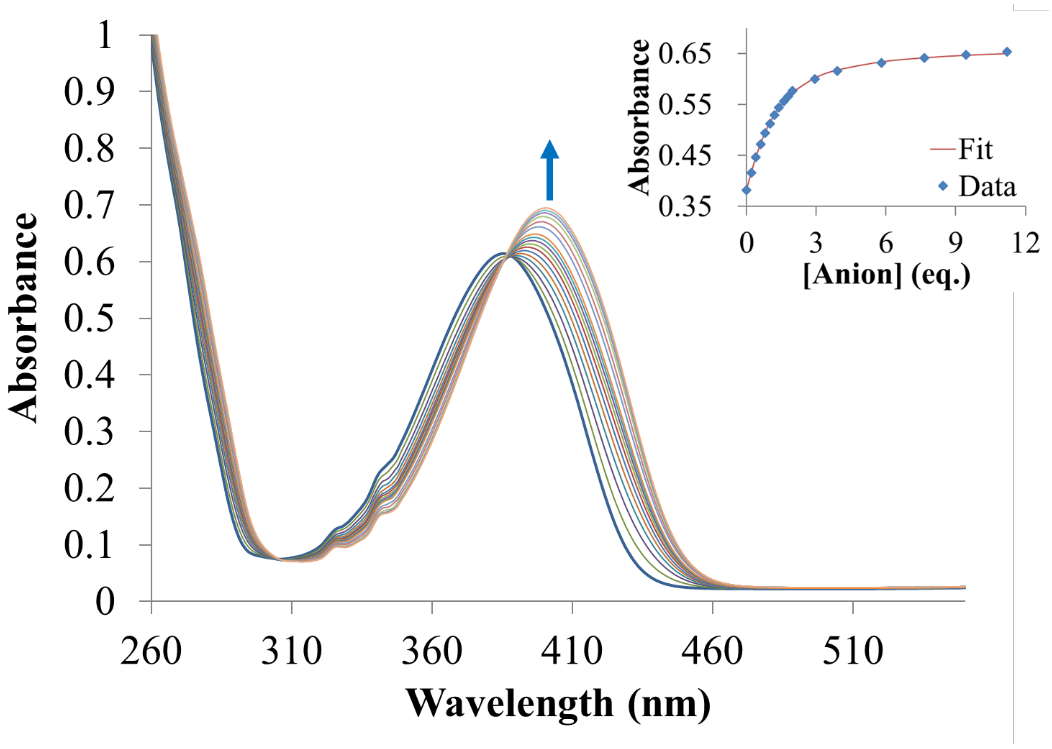

Figure S35 UV-Vis titration of **2** with TBAAcO (0 - 12 equiv.) in $CHCl_3/CH_3CN$ at 298 K.

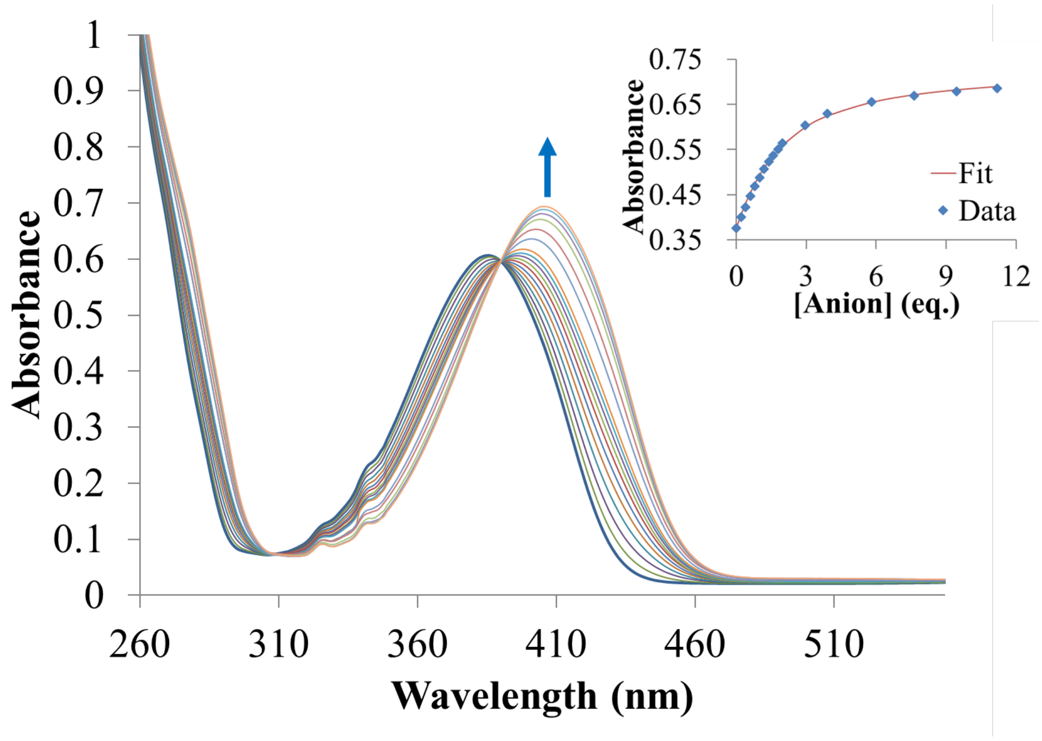

Figure S36 UV-Vis titration of **2** with TBAF (0 - 12 equiv.) in $CHCl_3/CH_3CN$ at 298 K.

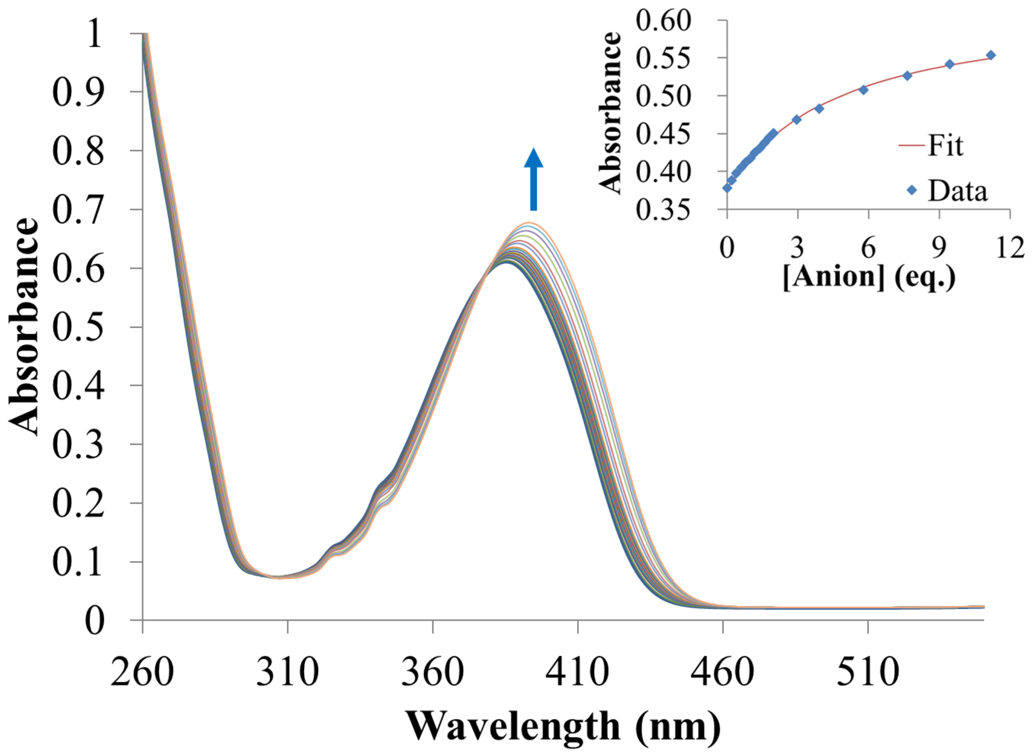

Figure S37 UV-Vis titration of **2** with TBACl (0 - 12 equiv.) in $CHCl_3/CH_3CN$ at 298 K.

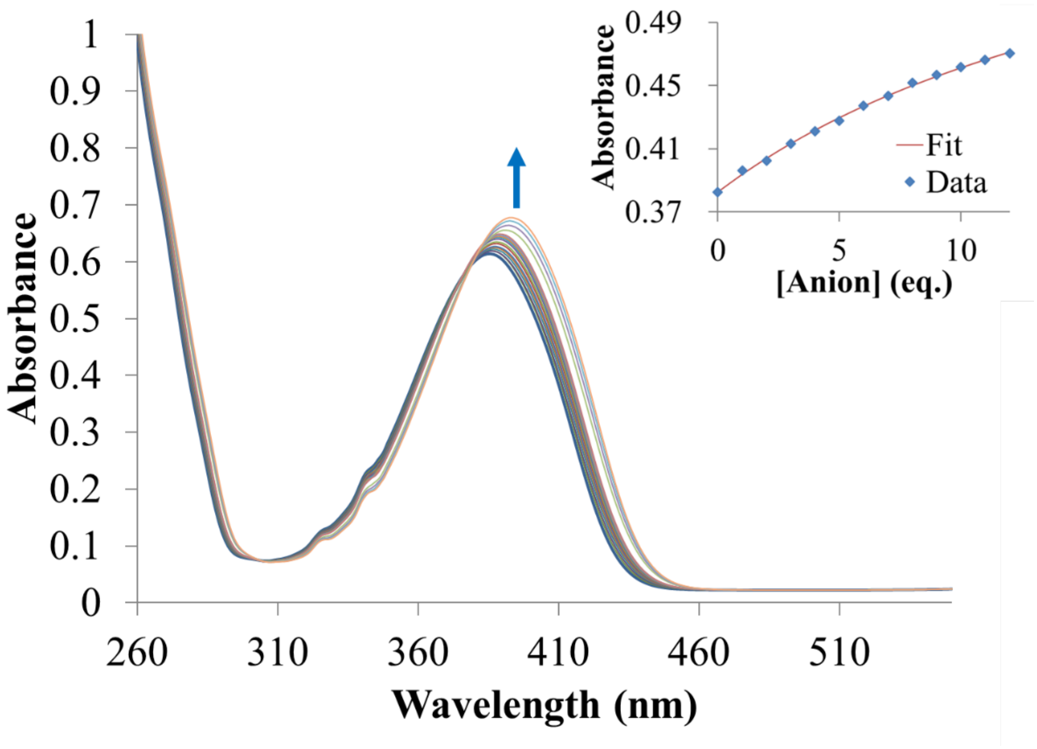

Figure S38 UV-Vis titration of **2** with TBAMsO (0 - 12 equiv.) in $CHCl_3/CH_3CN$ at 298 K.

Fluorescent titrations of axle 2

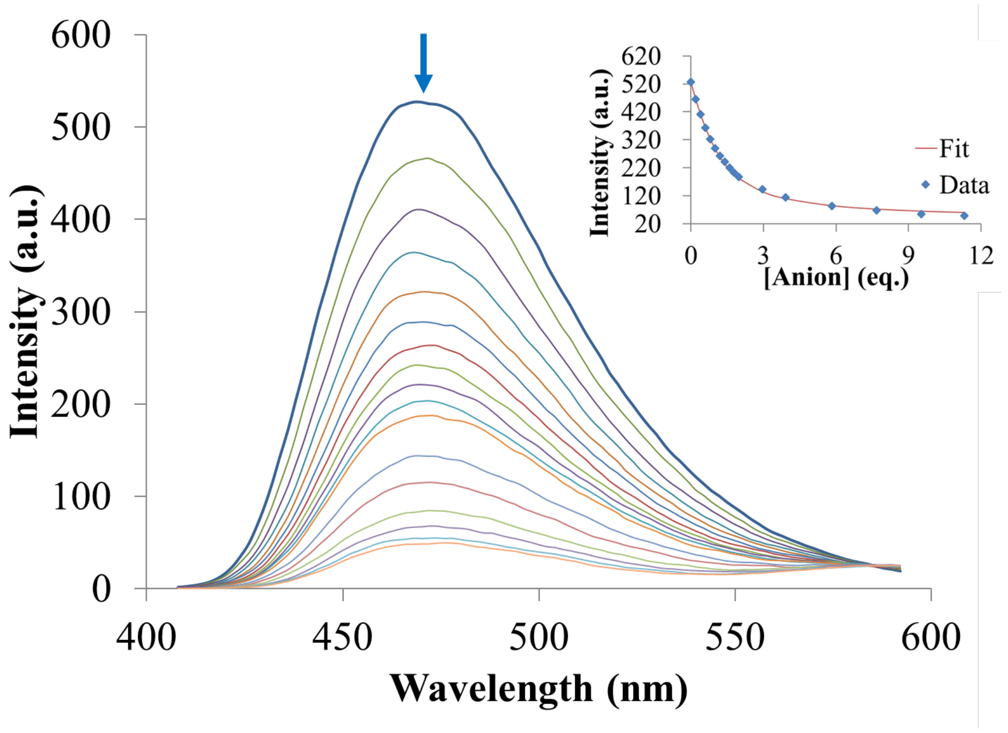

Figure S39 Fluorescence titration of **2** with TBAAcO (0 - 12 equiv.) in $CHCl_3/CH_3CN$ at 298 K.

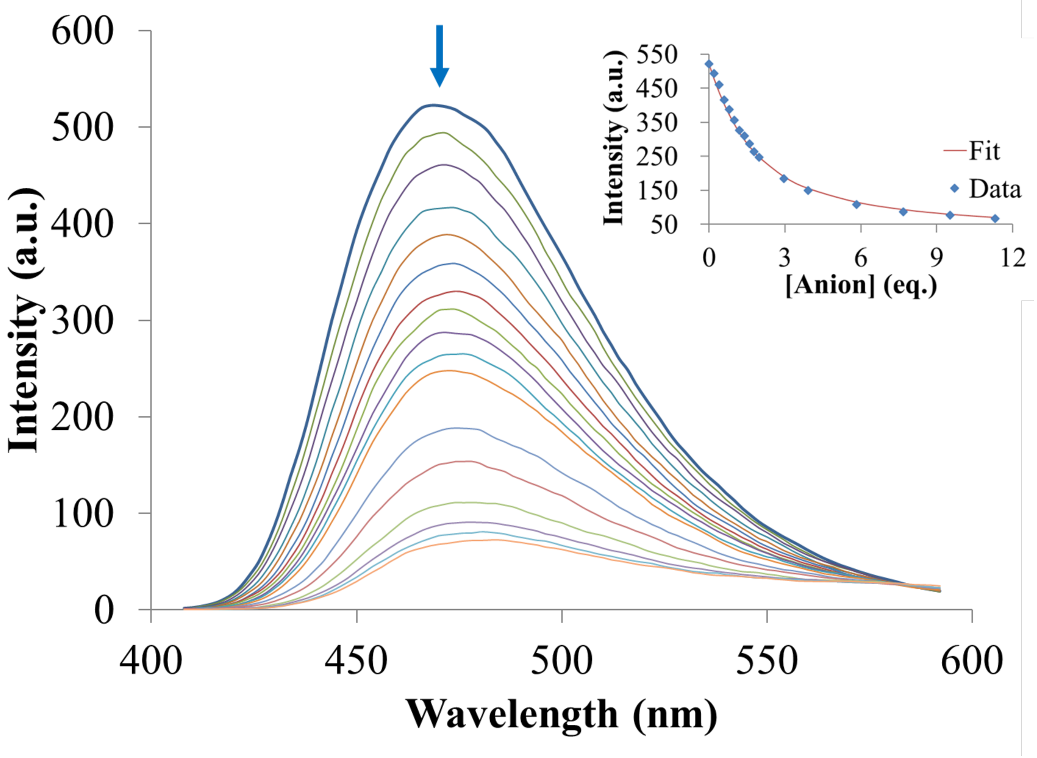

Figure S40 Fluorescence titration of **2** with TBAF (0 - 12 equiv.) in $CHCl_3/CH_3CN$ at 298 K.

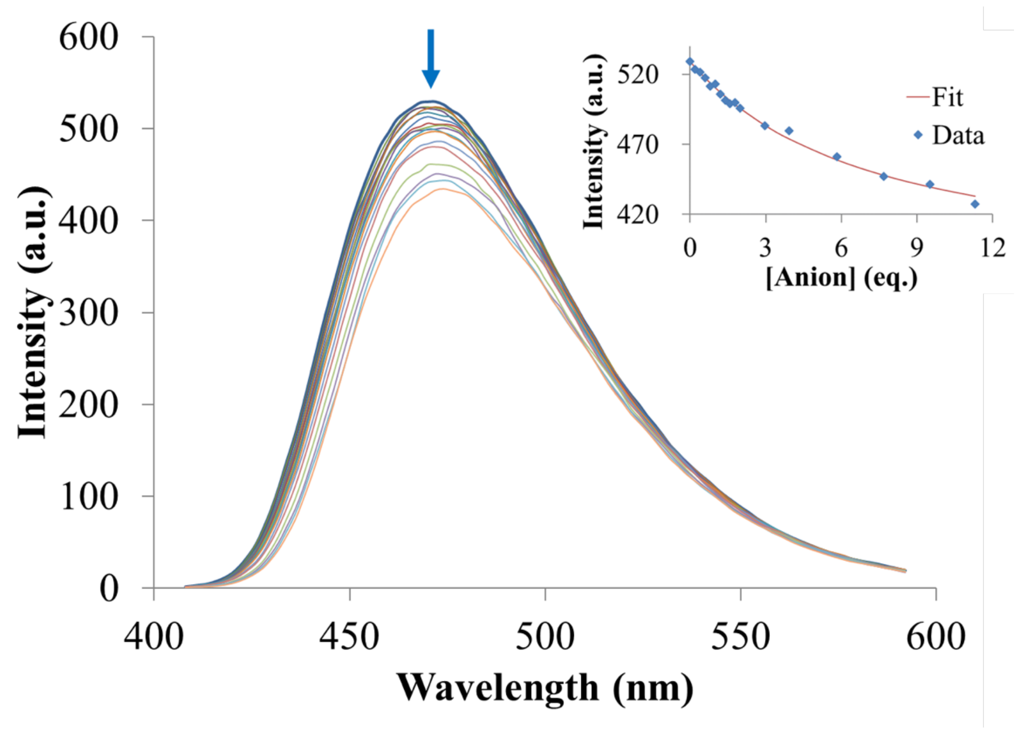

Figure S41 Fluorescence titration of **2** with TBACl (0 - 12 equiv.) in $CHCl_3/CH_3CN$ at 298 K.

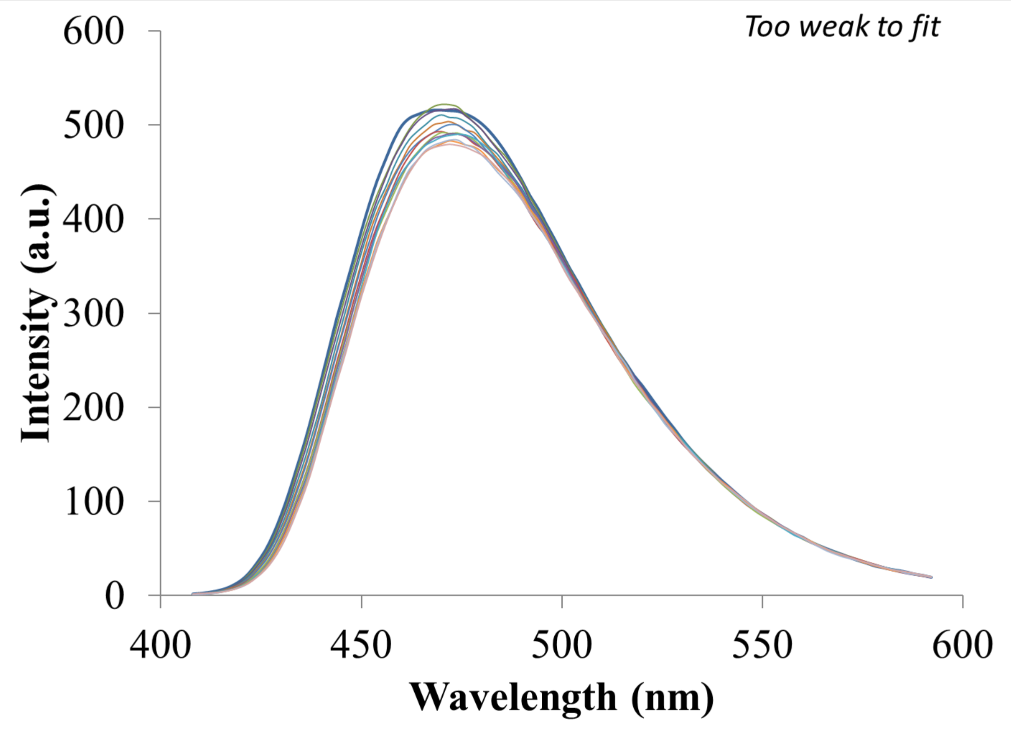

Figure S42 Fluorescence titration of **2** with TBAMsO (0 - 12 equiv.) in $CHCl_3/CH_3CN$ at 298 K.

^{1}H NMR titrations of rotaxane 1

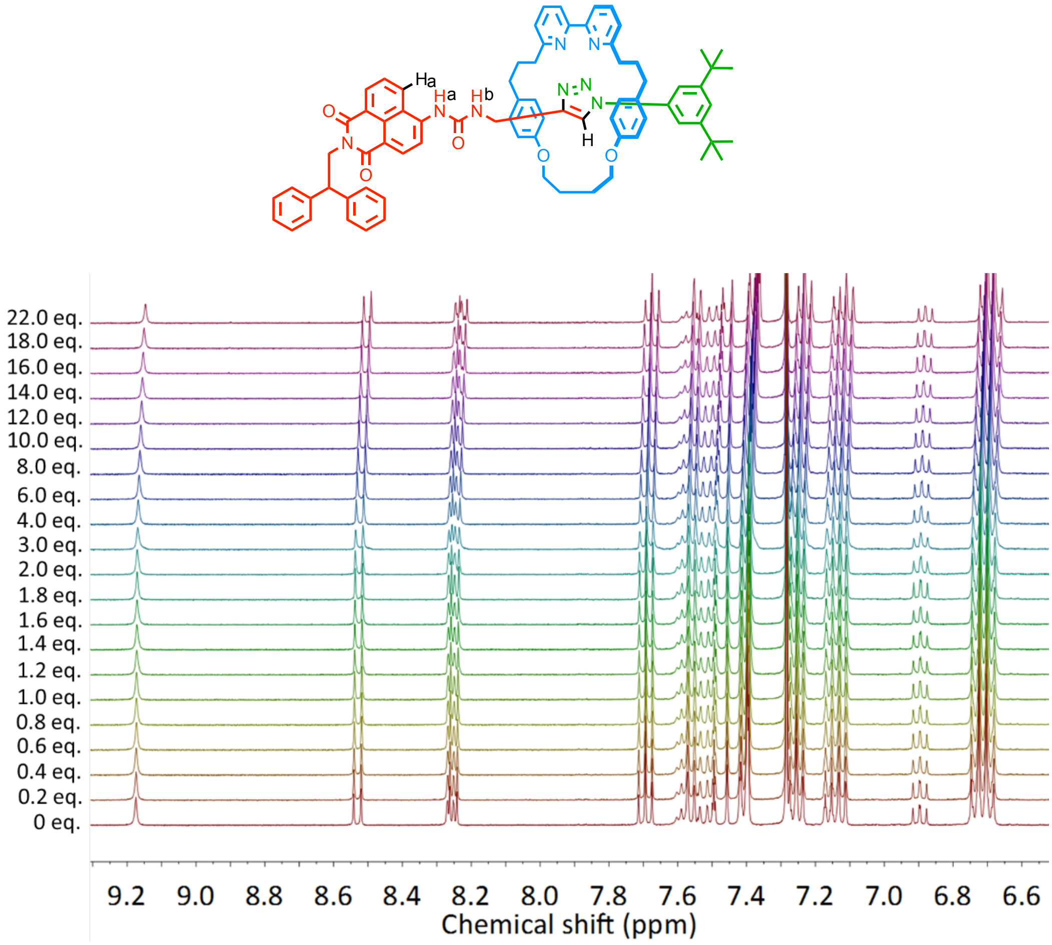

Figure S43 ^{1}H NMR titration of **1** with TBAAcO (0 - 12 equiv.) in $CDCl_3/CD_3CN$ at 298 K.

^{1}H NMR titrations of rotaxane 1.HBF$_4$

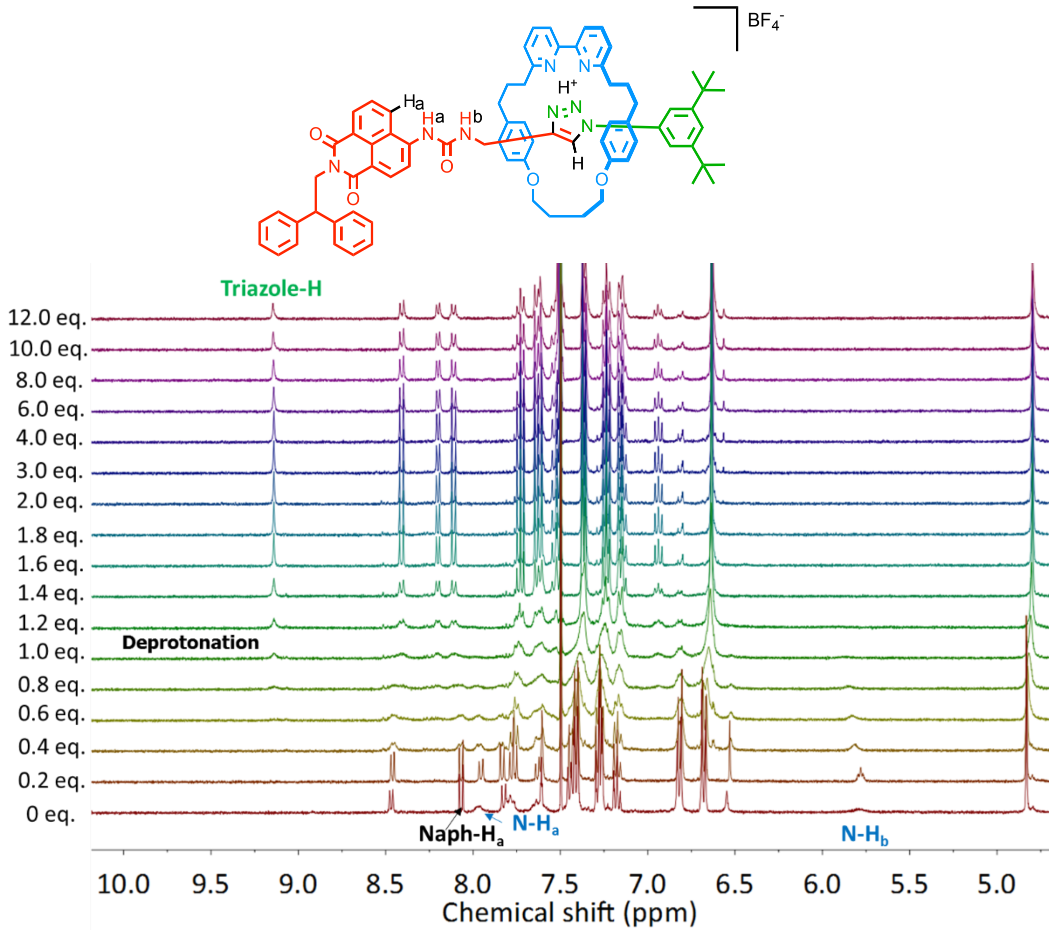

Figure S44 ^{1}H NMR titration of **1**.HBF$_4$ with TBAAcO (0 - 12 equiv.) in $CDCl_3/CD_3CN$ at 298 K (Fully deprotonation after addition of 1.0 equiv. of TBAAcO).

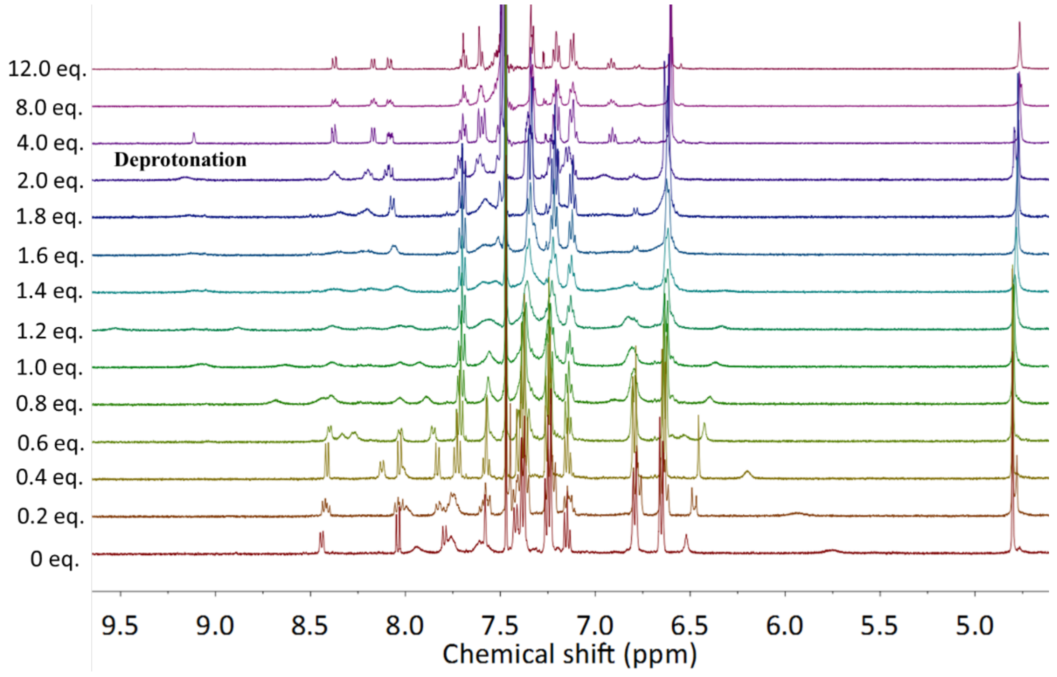

Figure S45 ^{1}H NMR titration of **1**.HBF$_4$ with TBAF (0 - 12 equiv.) in $CDCl_3/CD_3CN$ at 298 K (Fully deprotonation after addition of 2.0 equiv. of TBAF, further addition resulted in broadening of the triazole proton).

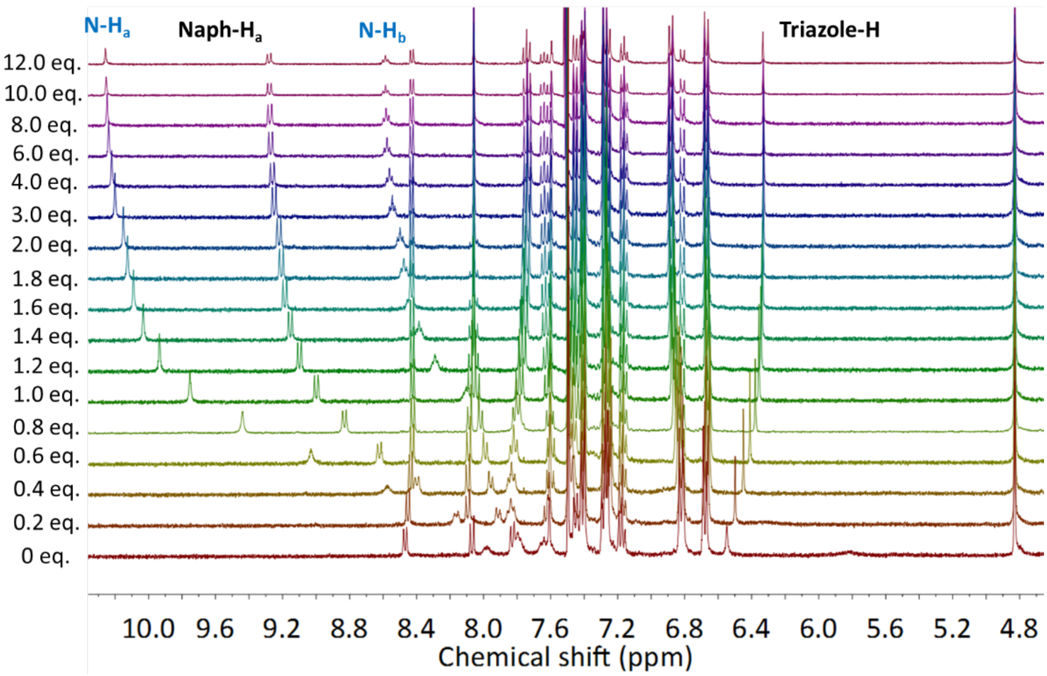

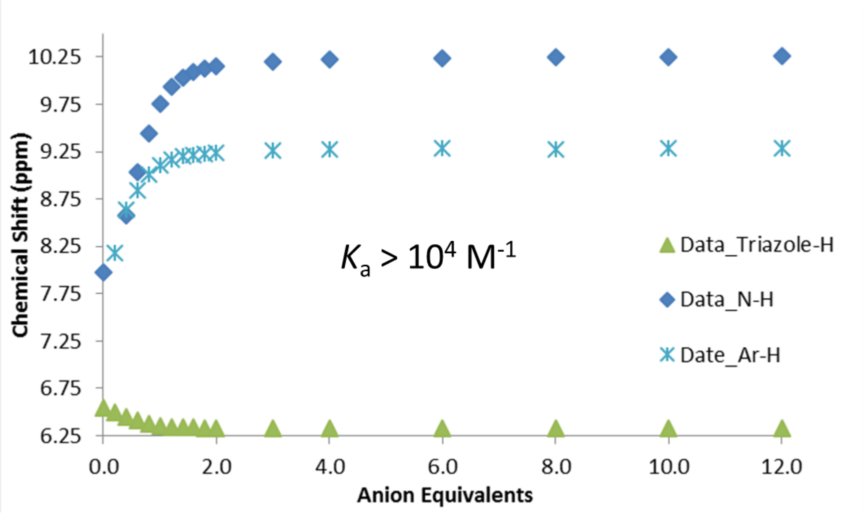

Figure S46 ^{1}H NMR titration of **1**.HBF_4 with TBACl (0 - 12 eq.) in $CDCl_3$/CD_3CN at 298 K.

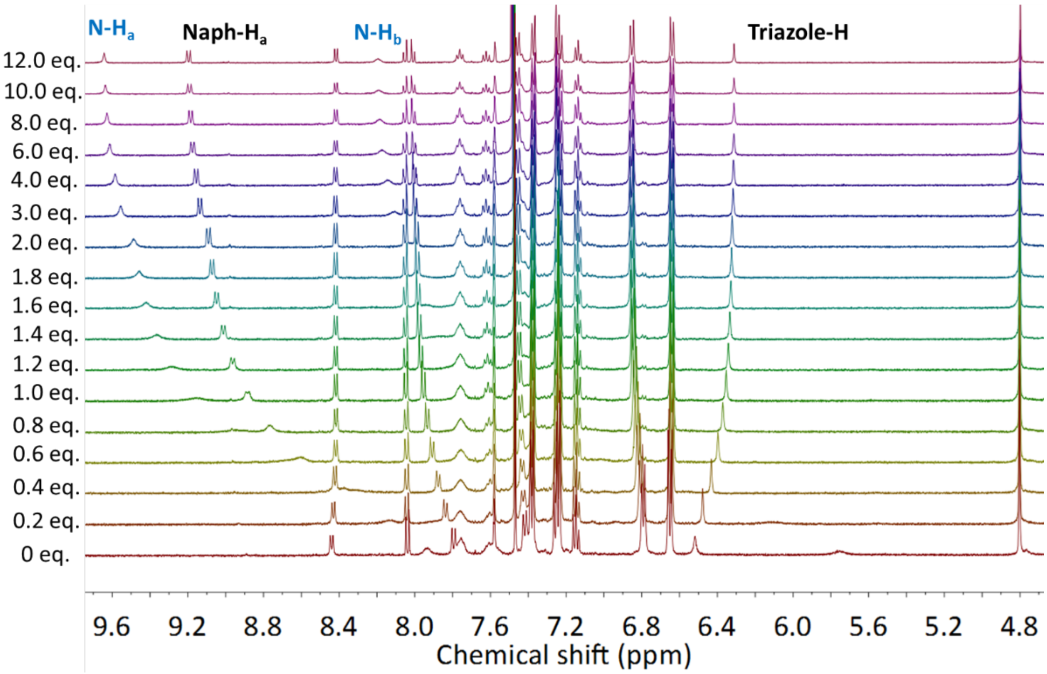

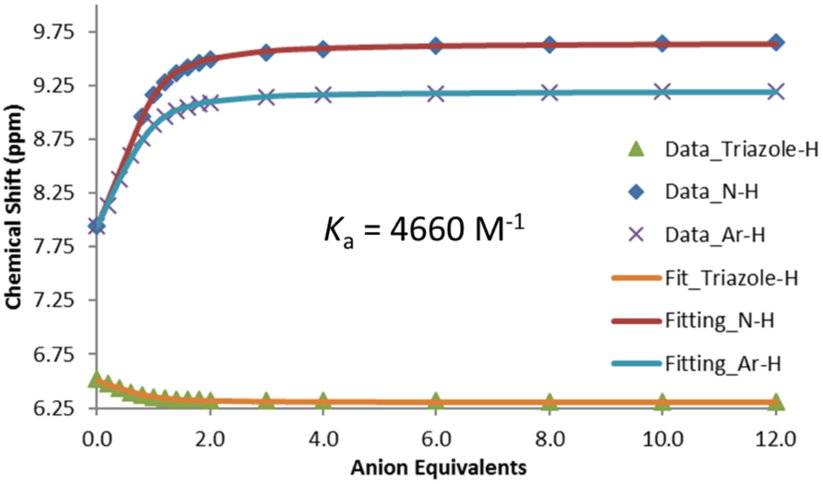

Figure S47 ^{1}H NMR titration of **1**.HBF_4 with TBABr (0 - 12 equiv.) in $CDCl_3/CD_3CN$ at 298 K.

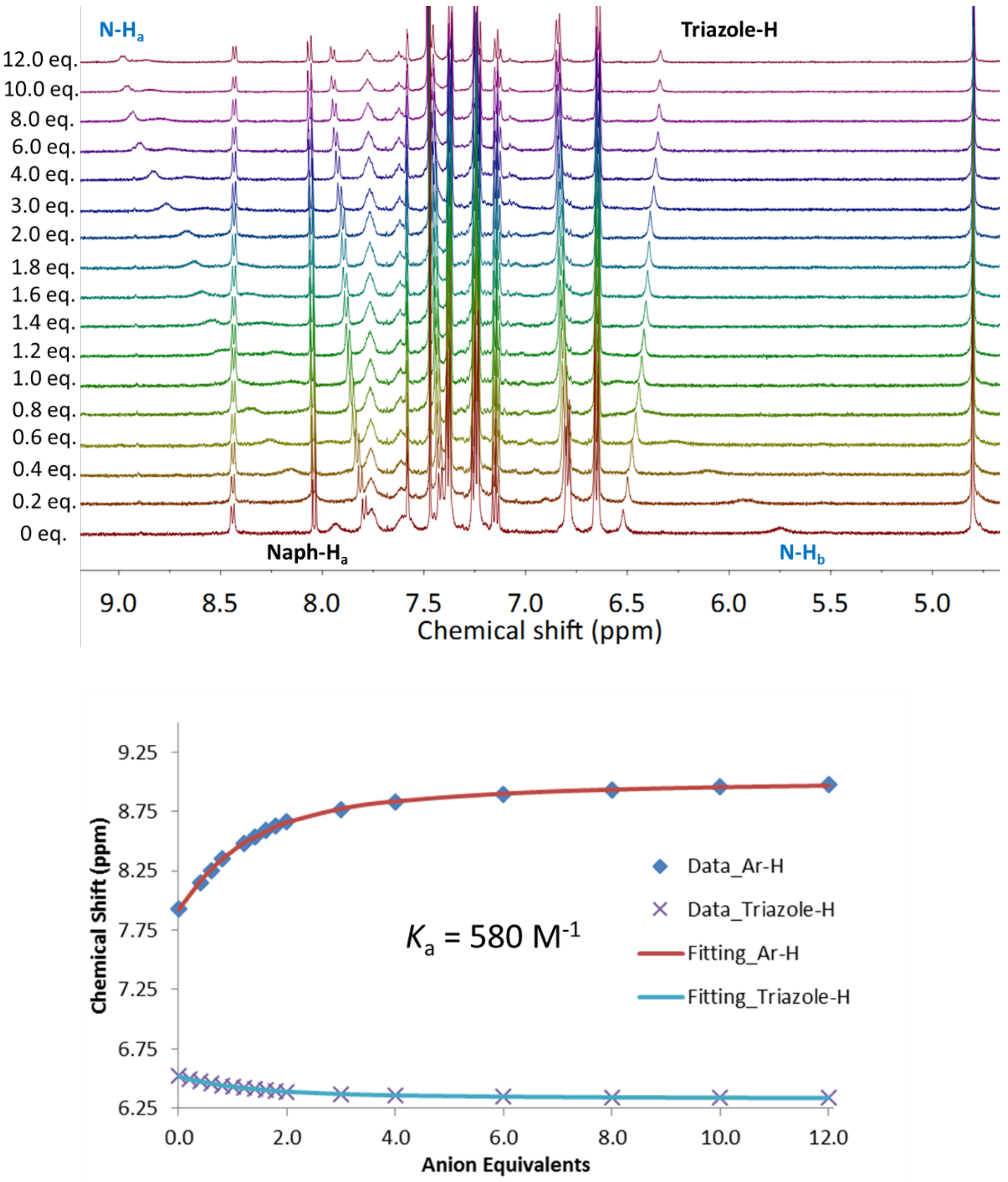

Figure S48 ^{1}H NMR titration of **1**.HBF_4 with TBAI (0 - 12 equiv.) in $CDCl_3/CD_3CN$ at 298 K.

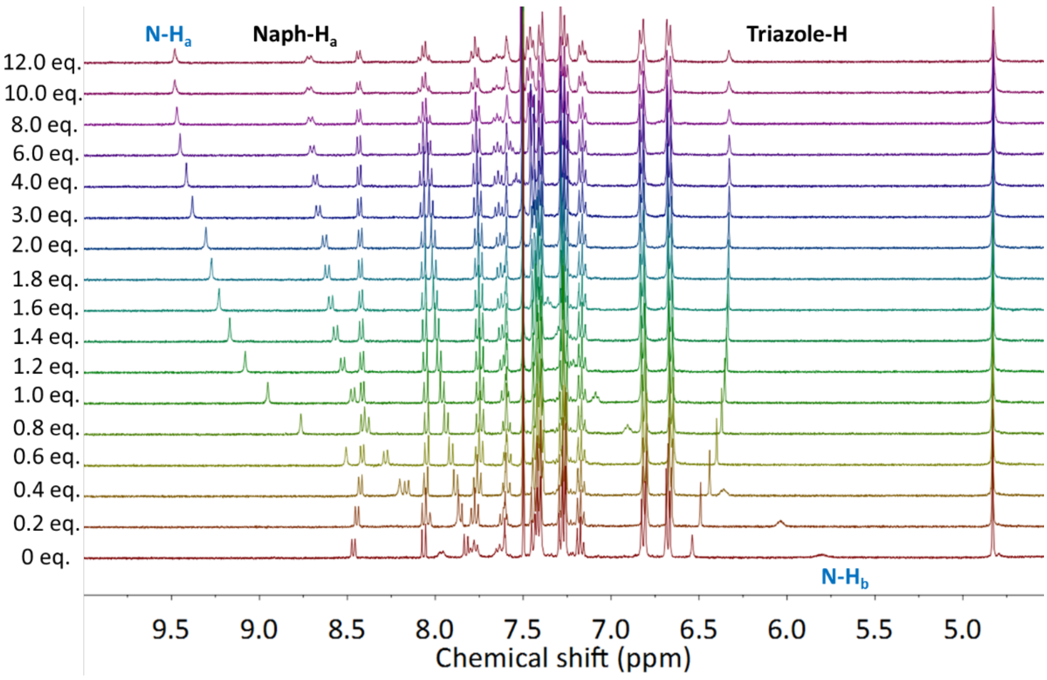

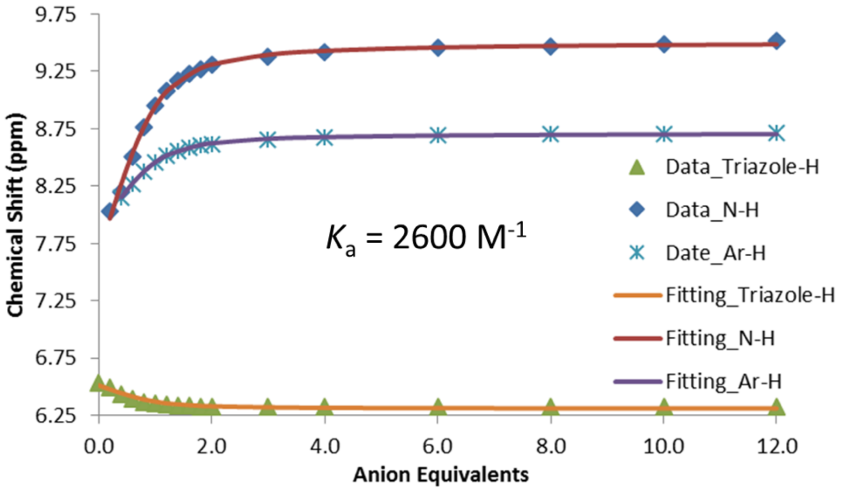

Figure S49 ^{1}H NMR titration of **1**.HBF_4 with TBAMsO (0 - 12 equiv.) in $CDCl_3/CD_3CN$ at 298 K.

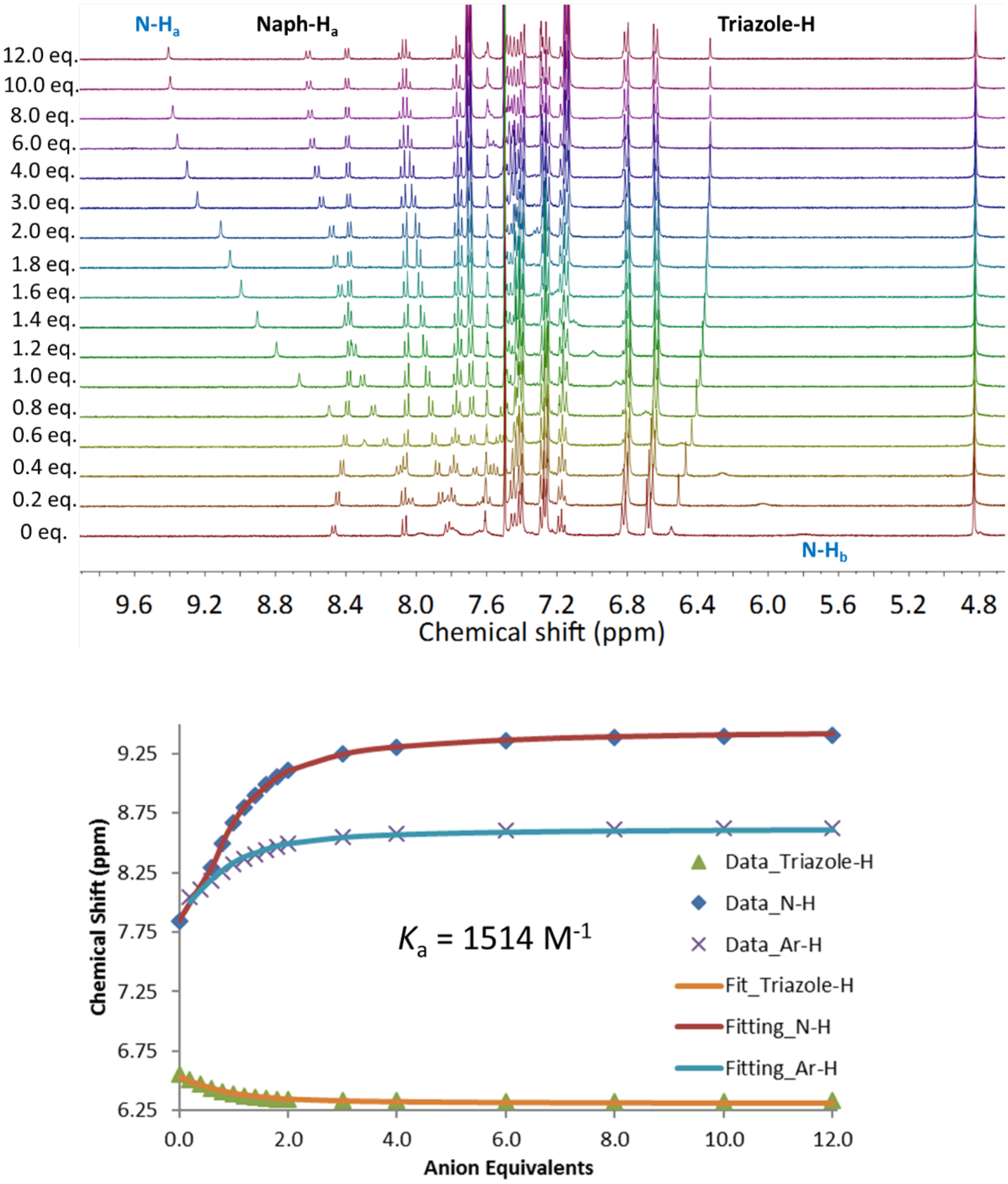

Figure S50 ^{1}H NMR titration of **1**.HBF_4 with TBATsO (0 - 12 equiv.) in $CDCl_3/CD_3CN$ at 298 K.

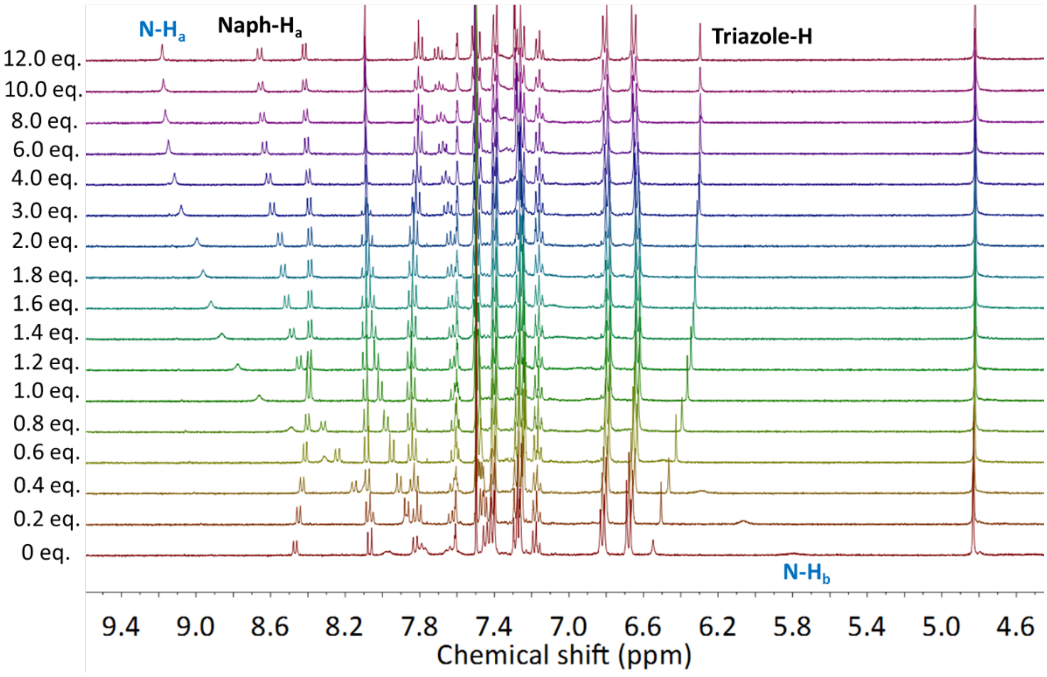

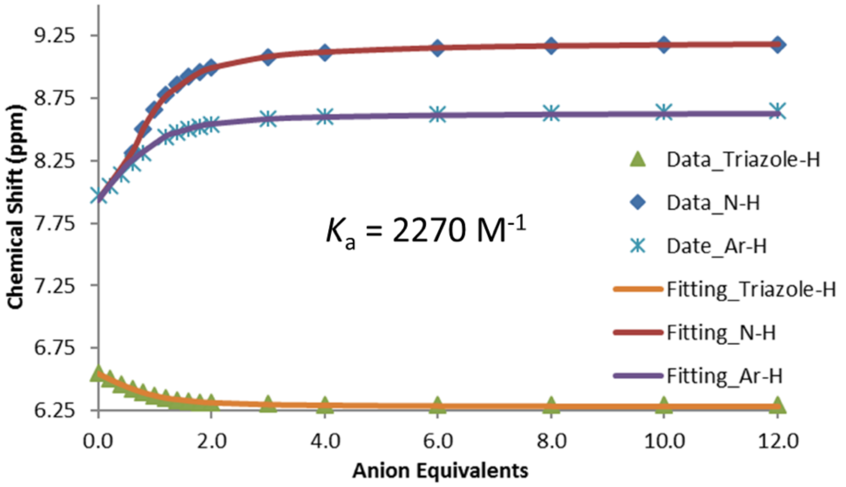

Figure S51 ^{1}H NMR titration of **1**.HBF_4 with $TBAHSO_4$ (0 - 12 equiv.) in $CDCl_3/CD_3CN$ at 298 K.

UV-Vis titrations of rotaxane 1·HBF_4

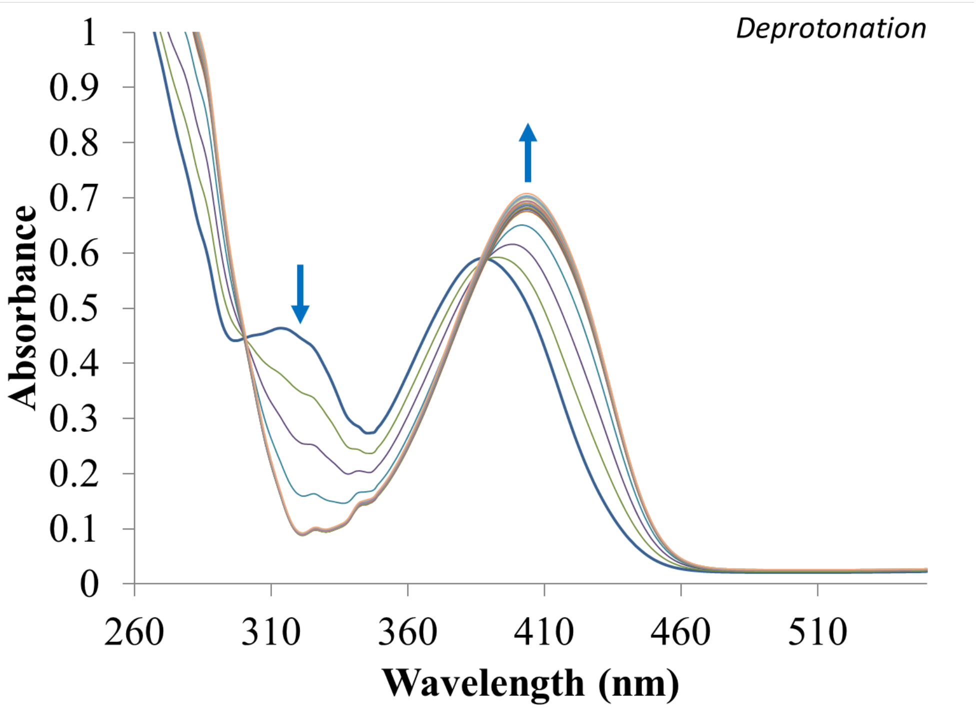

Figure S52 UV-Vis titration of **1**.HBF_4 with TBAAcO (0 - 12 equiv.) in $CHCl_3/CH_3CN$ at 298 K.

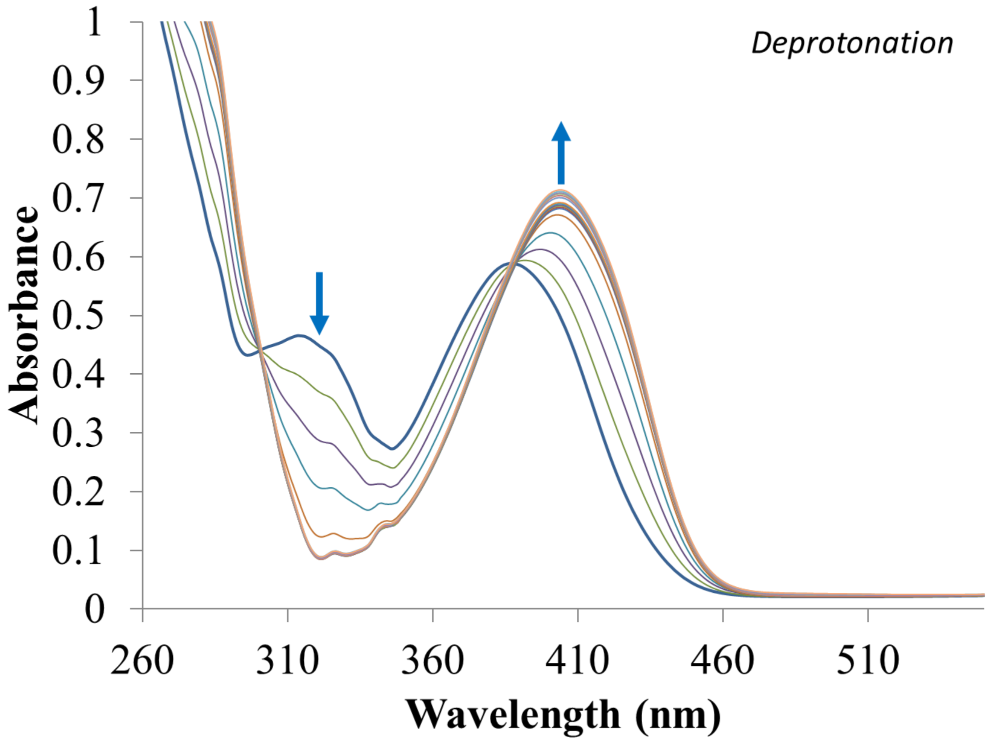

Figure S53 UV-Vis titration of **1**.HBF_4 with TBAF (0 - 12 equiv.) in $CHCl_3/CH_3CN$ at 298 K.

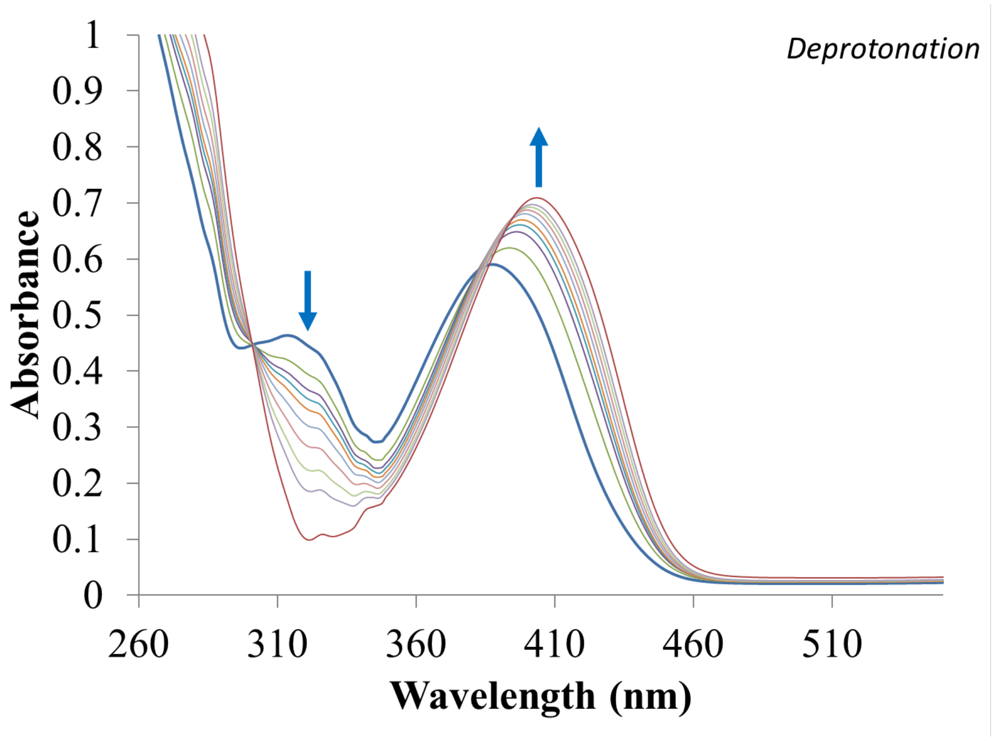

Figure S54 UV-Vis titration of **1**.HBF_4 with TBAOH (0 - 12 equiv.) in $CHCl_3/CH_3CN$ at 298 K.

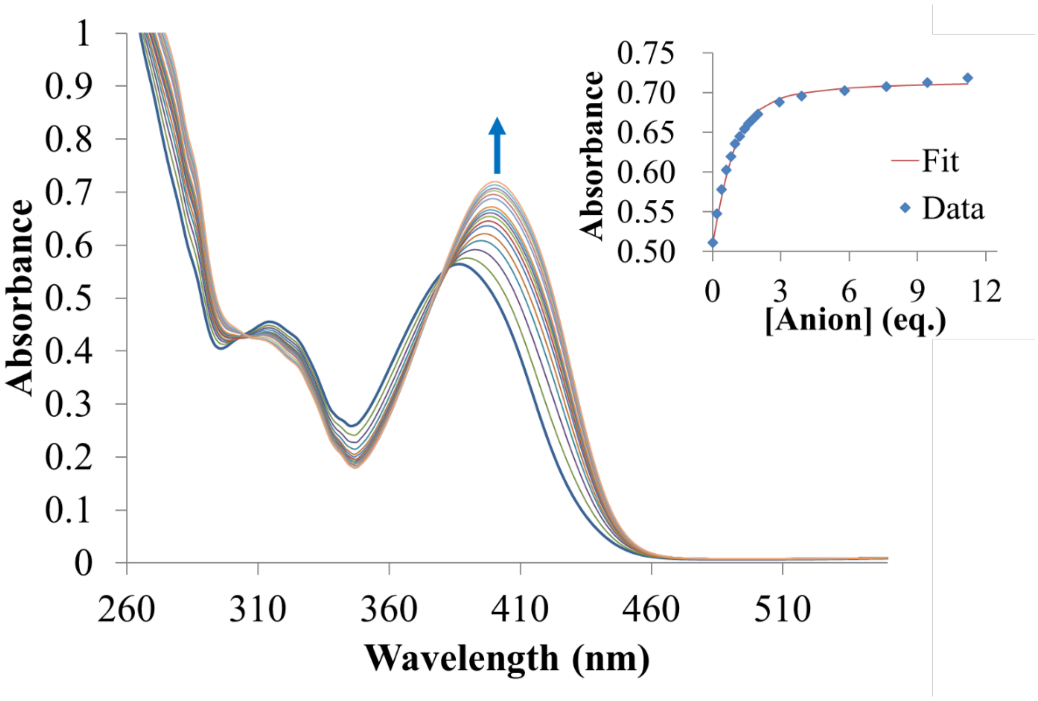

Figure S55 UV-Vis titration of **1**.HBF_4 with TBACl (0 - 12 equiv.) in $CHCl_3/CH_3CN$ at 298 K.

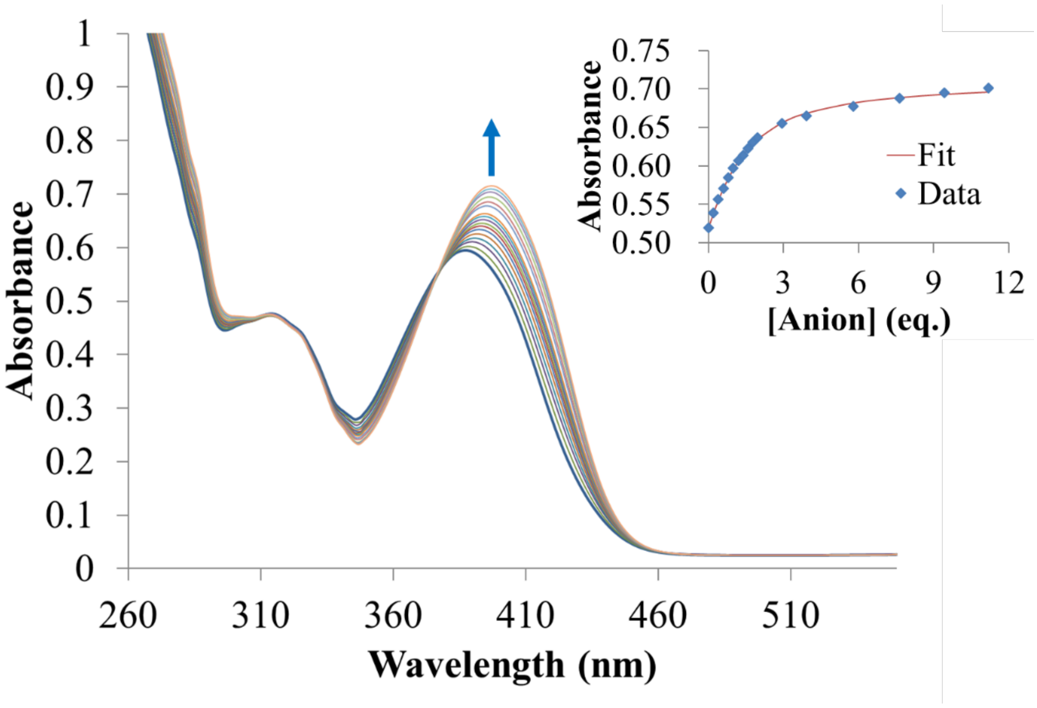

Figure S56 UV-Vis titration of **1**.HBF_4 with TBABr (0 - 12 equiv.) in $CHCl_3/CH_3CN$ at 298 K.

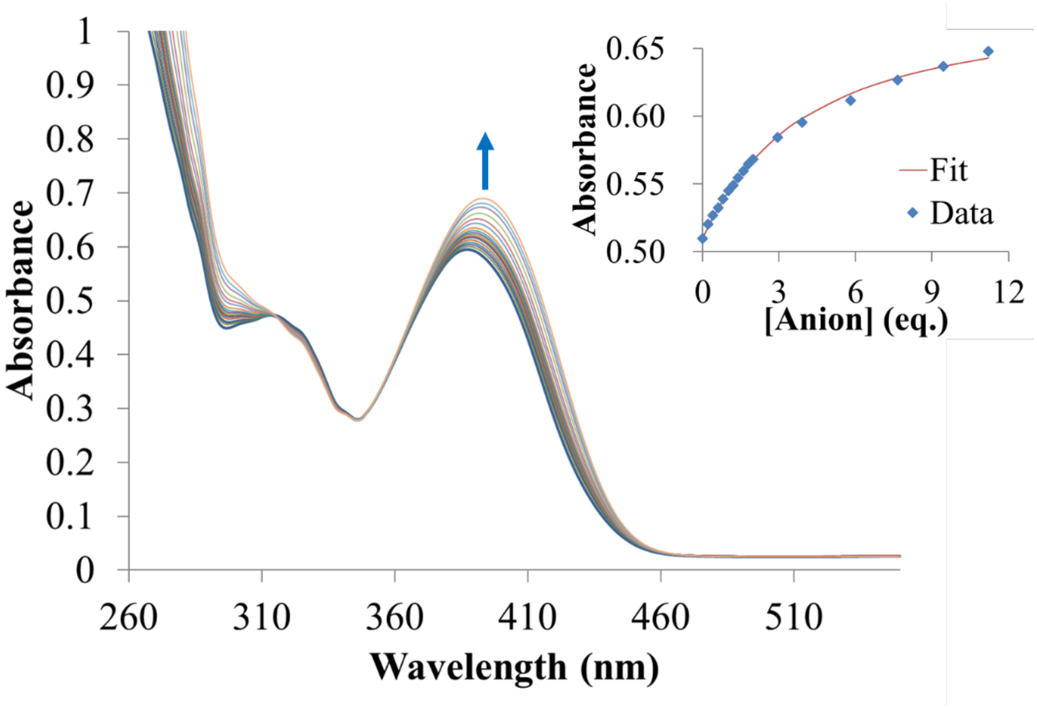

Figure S57 UV-Vis titration of **1**.HBF_4 with TBAI (0 - 12 equiv.) in $CHCl_3/CH_3CN$ at 298 K.

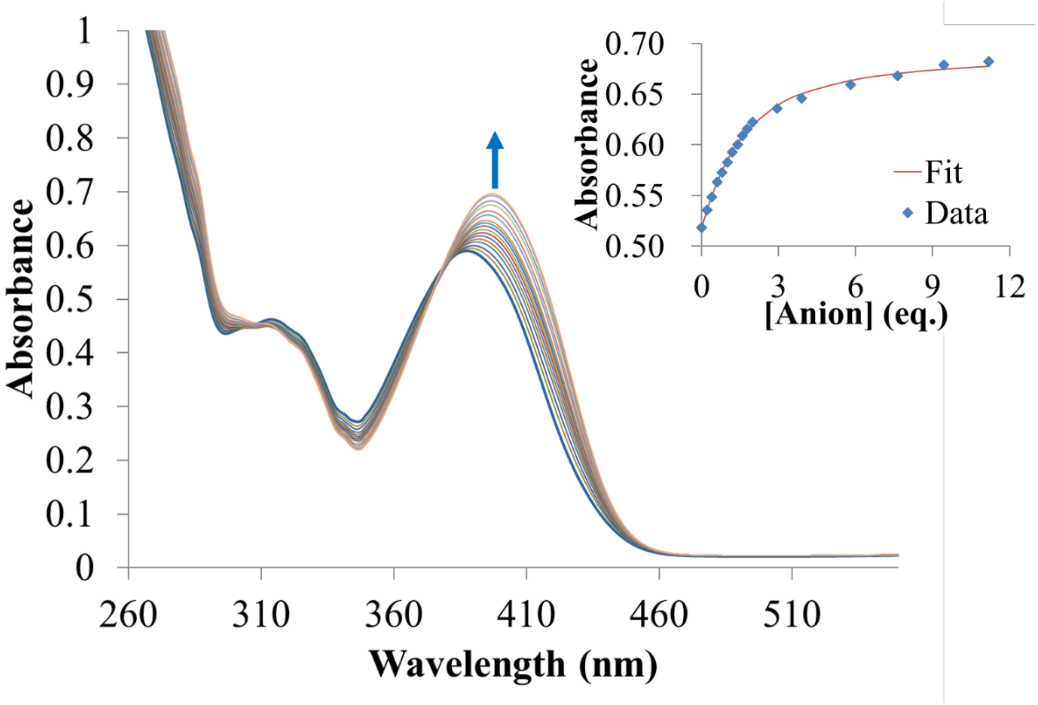

Figure S58 UV-Vis titration of **1**.HBF_4 with TBAMsO (0 - 12 equiv.) in $CHCl_3/CH_3CN$ at 298 K.

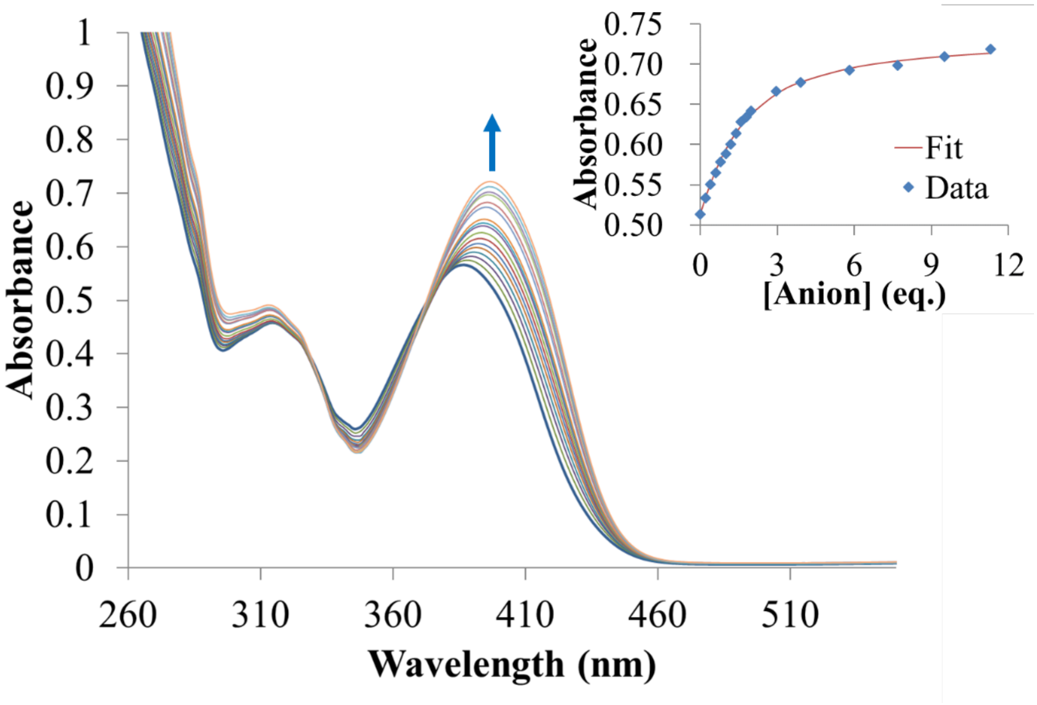

Figure S59 UV-Vis titration of **1**.HBF_4 with TBATsO (0 - 12 equiv.) in $CHCl_3/CH_3CN$ at 298 K.

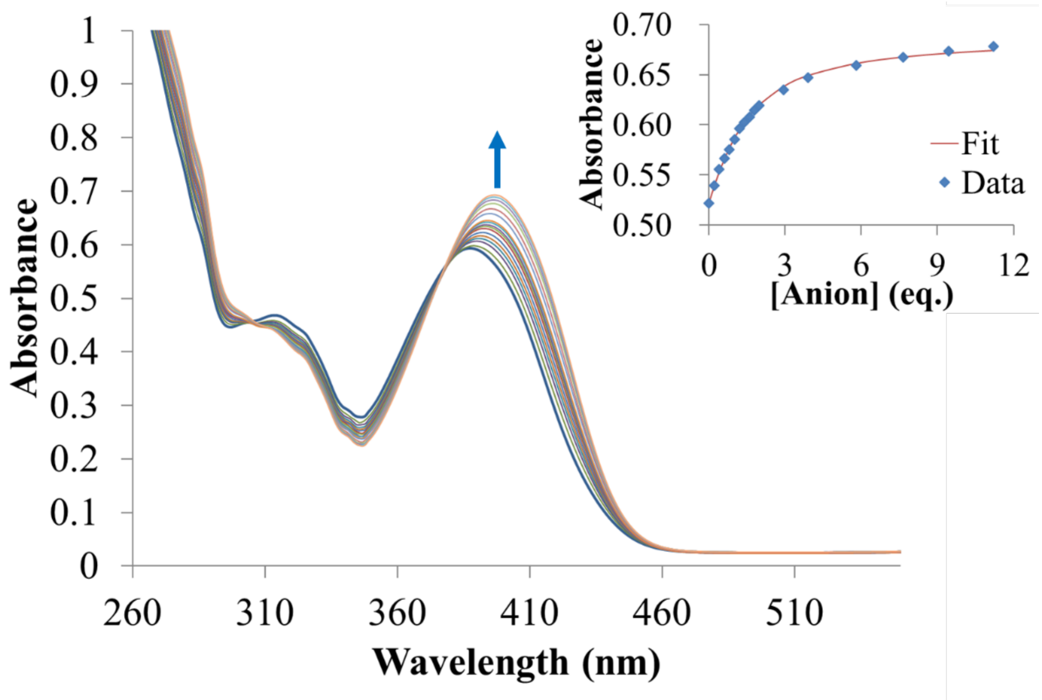

Figure S60 UV-Vis titration of **1**.HBF_4 with $TBAHSO_4$ (0 - 12 equiv.) in $CHCl_3/CH_3CN$ at 298 K.

Fluorescence titrations of rotaxane 1·HBF_4

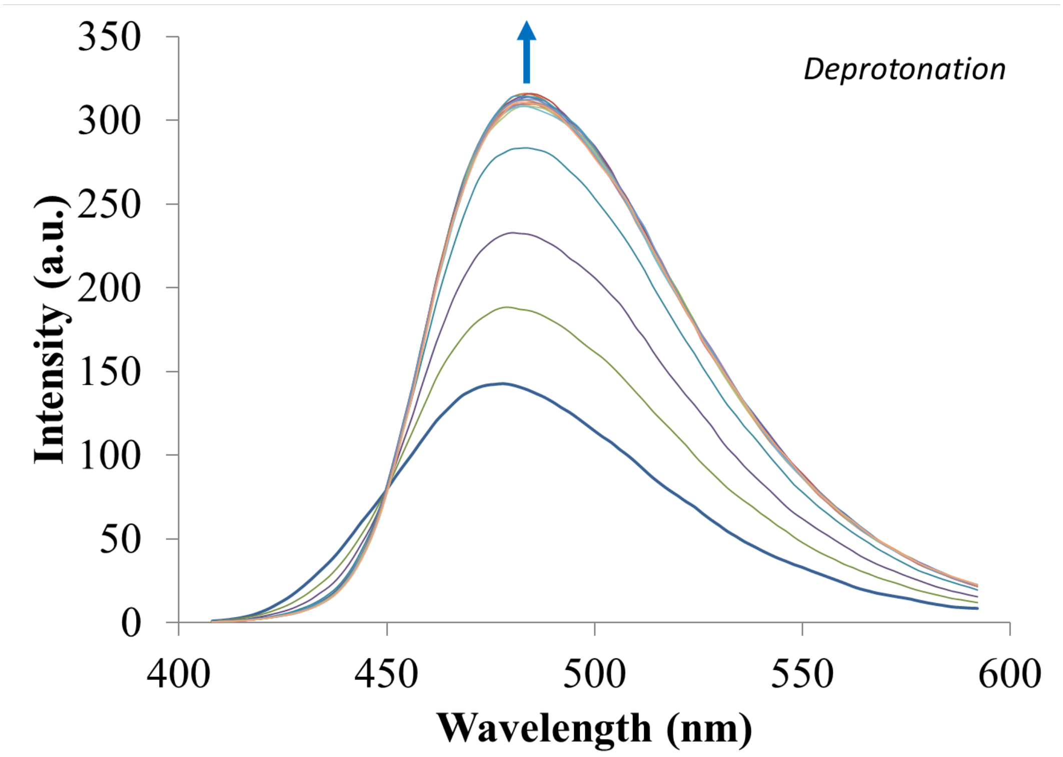

Figure S61 Fluorescence titration of **1**.HBF_4 with TBAAcO (0 - 12 equiv.) in $CHCl_3/CH_3CN$ at 298 K.

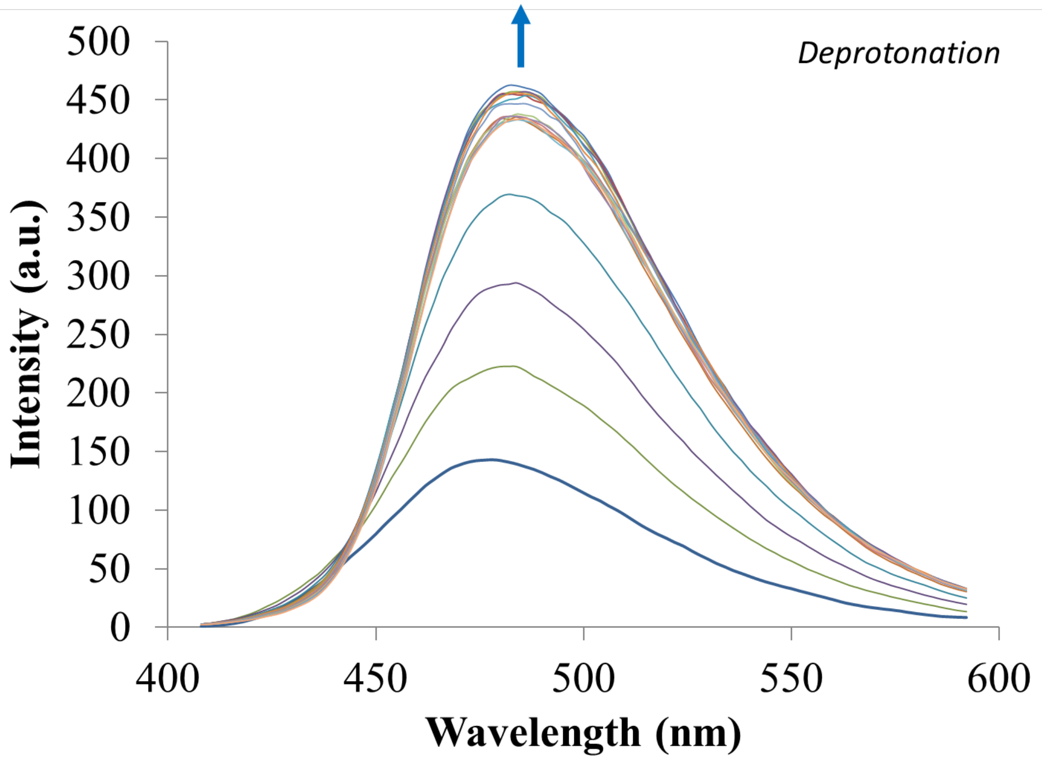

Figure S62 Fluorescence titration of **1**.HBF_4 with TBAF (0 - 12 equiv.) in $CHCl_3/CH_3CN$ at 298 K.

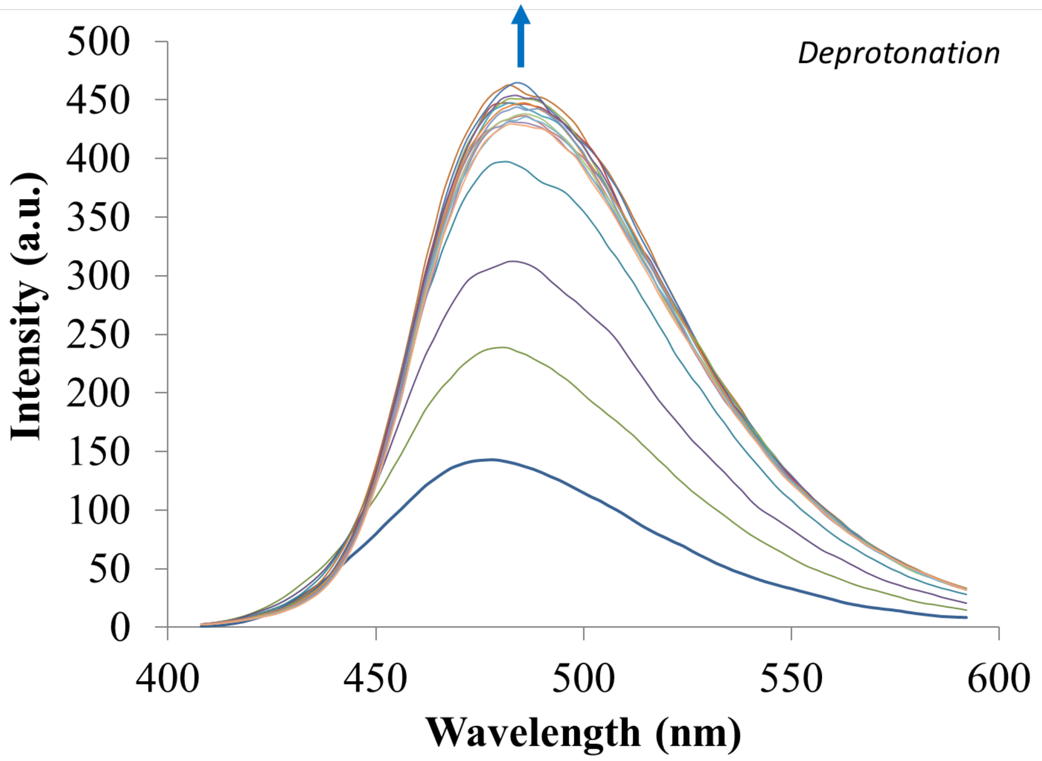

Figure S63 Fluorescence titration of **1**.HBF_4 with TBAOH (0 - 12 equiv.) in $CHCl_3/CH_3CN$ at 298 K.

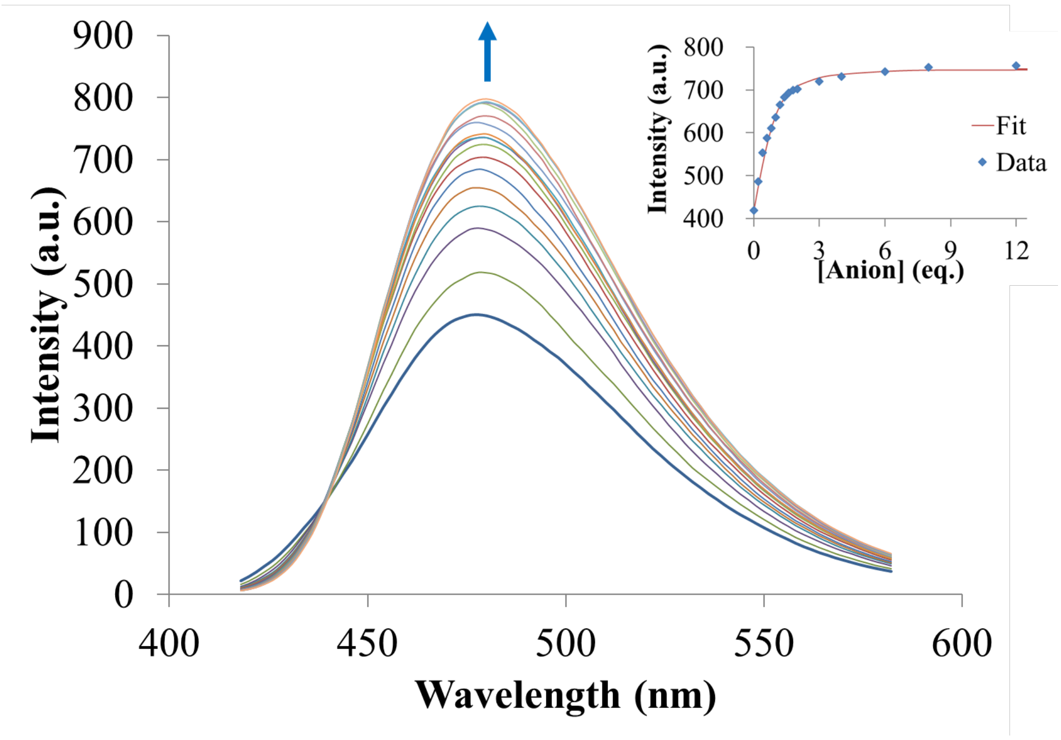

Figure S64 Fluorescence titration of **1**.HBF_4 with TBACl (0 - 12 equiv.) in $CHCl_3/CH_3CN$ at 298 K.

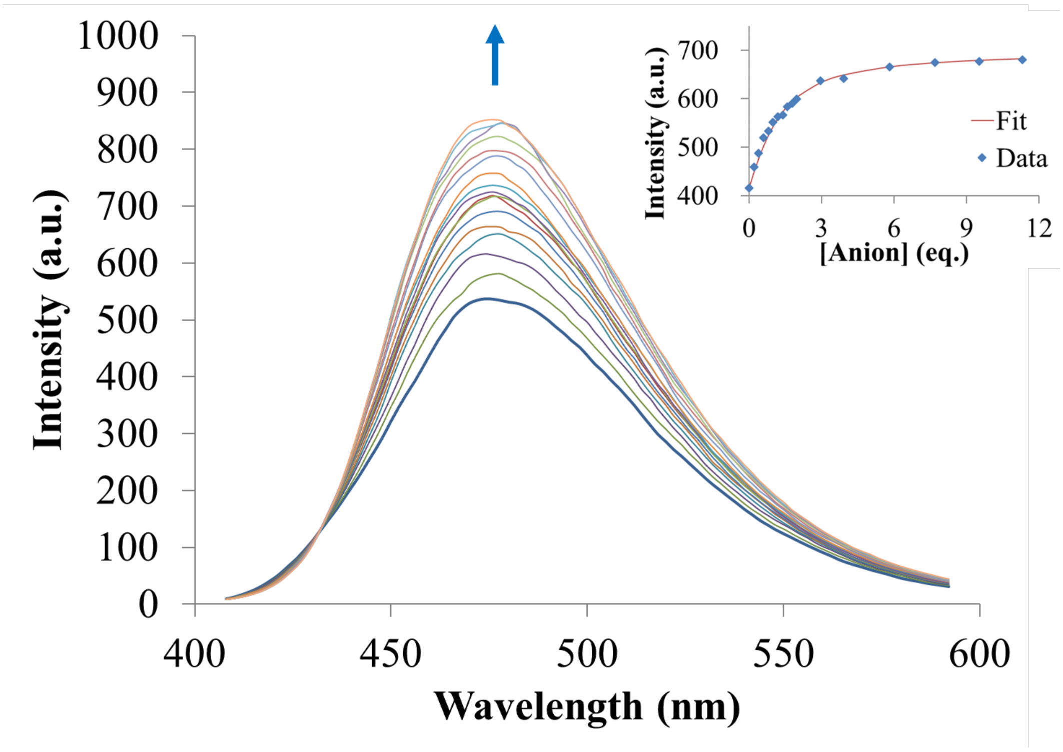

Figure S65 Fluorescence titration of **1**.HBF_4 with TBABr (0 - 12 equiv.) in $CHCl_3/CH_3CN$ at 298 K.

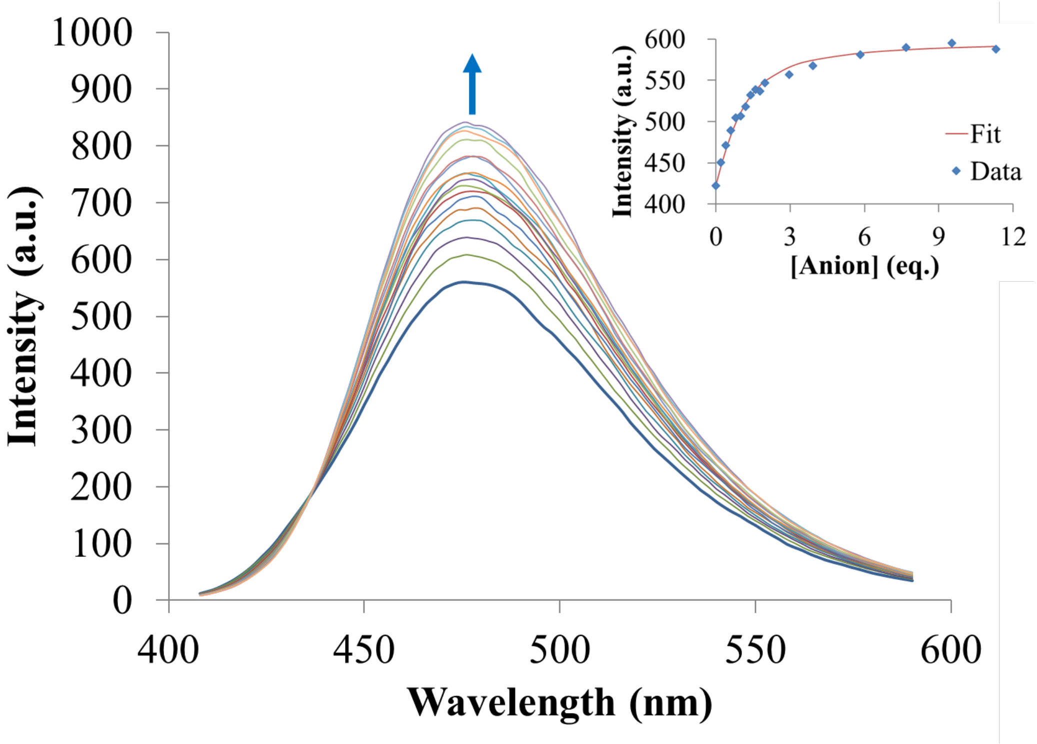

Figure S66 Fluorescence titration of **1**.HBF_4 with TBAMsO (0 - 12 equiv.) in $CHCl_3/CH_3CN$ at 298 K.

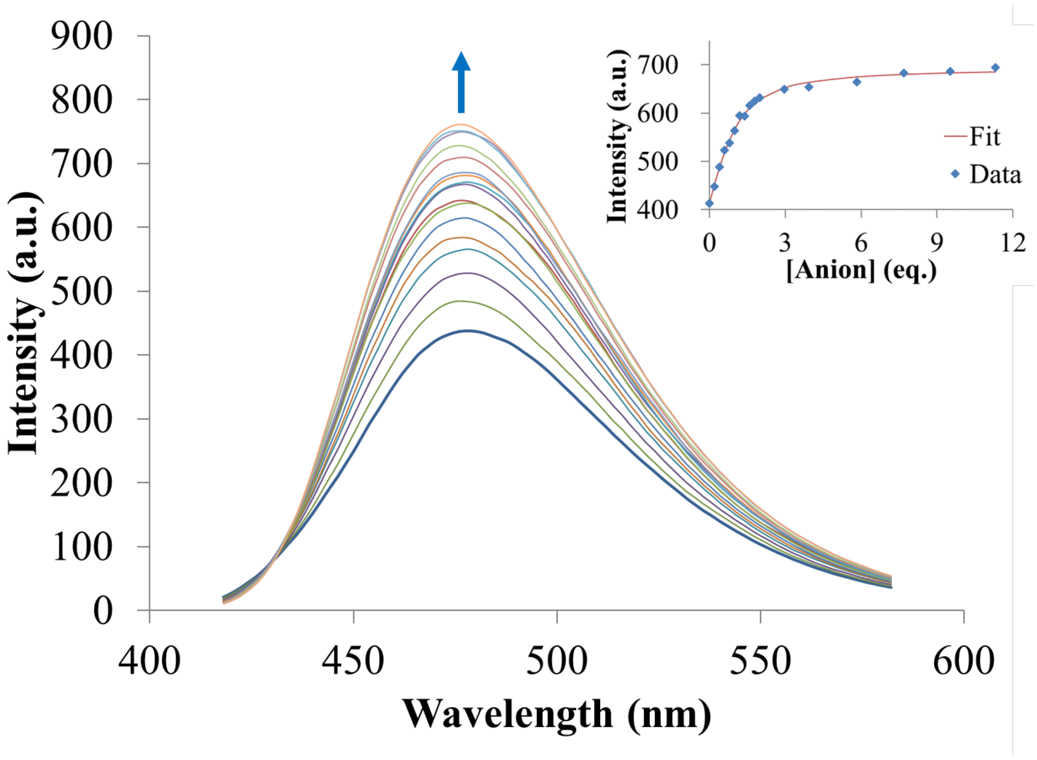

Figure S67 Fluorescence titration of **1**.HBF_4 with TBATsO (0 - 12 equiv.) in $CHCl_3/CH_3CN$ at 298 K.

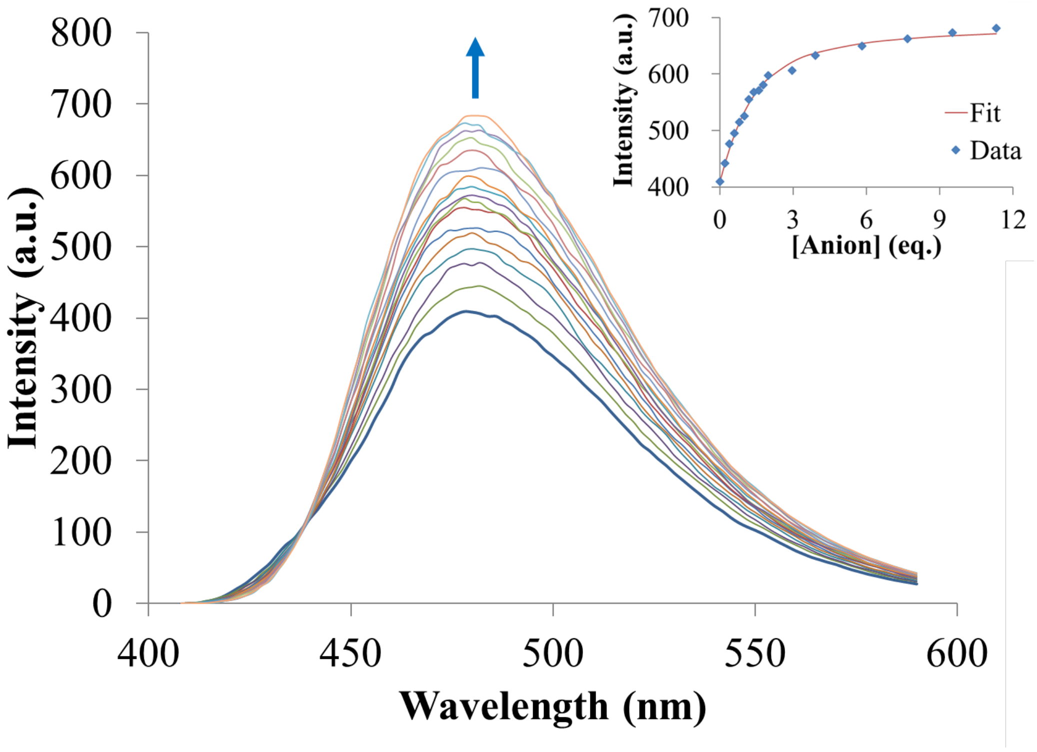

Figure S68 Fluorescence titration of **1**.HBF_4 with $TBAHSO_4$ (0 - 12 equiv.) in $CHCl_3/CH_3CN$ at 298 K.

Single Crystal X-ray Analysis Data

Single Crystal X-ray Diffraction Characterisation of 1, 1.HBF$_4$, 1.HCl and 1.HBr

Crystals of **1** were obtained from slow cooling of a EtOH/H_2O mixture. Crystals of **1**.HBF_4 binding Cl/Br were obtained from slow evaporation of a MeCN/Et_2O mixture of **1**.HBF_4 in the presence of TBACl/Br (10 equiv.).

Single crystal X-ray diffraction data for **1**, **1**.HBF_4 and **1**.HCl were collected at the University of Southampton, while data for 1.HBr were collected at the University of Sydney. Data for **1** were collected at 100K using a monochromated Mo(Kα) radiation generated from a Rigaku FRE+ rotating anode source equipped with an AFC12 kappa goniometer, HF Varimax confocal mirrors and a HG Saturn 724+ CCD detector. Data were collected 100 K with ω scans to 64º 2θ and cell constants were obtained from a least squares refinement against 11,547 reflections located between 3 and 60º 2θ. Data for **1**.HBF_4 and **1**.HCl were collected using a Rigaku MicroMax 007 instrument generating Cu(Kα) radiation from a rotating anode and equipped with an AFC11 quarter-chi goniometer, Varimax focusing mirrors and a Saturn 944 CCD detector. Data for **1**.HBF_4 were collected 100 K with ω scans to 142º 2θ and cell constants were obtained from a least squares refinement against 9,873 reflections located between 8 and 119º 2θ. Data for **1**.HCl were collected with ω scans to 142º 2θ and cell constants were obtained from a least squares refinement against 18,178 reflections located between 7 and 130º 2θ. Data for **1**.HBr were collected using a SuperNova Dual diffractometer equipped with a four-circle kappa goniometer, an Atlas CCD detector and employing mirror monochromated Cu(Kα) radiation generated from a micro-source. Data were collected 150 K with ω

scans to 137º 2θ. Cell constants were obtained from a least squares refinement against 33,984 reflections located between 7 and 152º 2θ.

Data processing was undertaken with CrysalisPro[3] and included the application of a multi-scan absorption correction. Subsequent computations were carried out with the assistance of the WinGX[4,5], ShelXle[6] and OLEX2[7] interfaces. The structures of **1**, **1**.HBF_4 and **1**.HCl were obtained using SUPERFLIP[8], while that for **1**.HBr was obtained using SHELXT. The structures were extended and refined with SHELXL-2017/1.[9] Some of the geometry calculations were undertaken with XTAL.[10]

The **1**.HBF_4, **1**.HCl and **1**.HBr structures are essentially isostructural. The napthalamide residues of the rotaxane's 'axle' was found to be disordered over two orientations in the **1**.HBF_4, **1**.HCl and **1**.HBr structures. Modelling the disorder included the use of a rigid body for the **1**.HCl and **1**.HBr structures, for which coordinates were obtained from the Cambridge Structural Database (CSD reference SINBUN).[11,129] Additionally, one of the t-butyl residues was found to be disordered over at least two orientations in the **1**.HCl and **1**.HBr structures. The disorder is evidently 'imposed' by otherwise impossibly close intermolecular contacts. The disorder is present in the *P*1 non-centrosymmetric structure and there was no evidence of a supercell that might 'resolve' the disorder. Significant residual electron associated with substantial channels in the **1**.HBF_4, **1**.HCL and **1**.HBr (see for example **Figure S69**) and voids in the structure of 1 prompted the use of SQUEEZE.[11]

The numbering scheme is represented in **Figure S70**, which depicts[7] **1**.HBr with displacement ellipsoids at the 50% level is provided in Figure S2 and the numbering schemes for the axle and the macrocycle are shown in **Figure S71** and **Figure S72**. Crystallographic details are provided in Table S1 and selected hydrogen bond/contact details are provided in Tables S2-S4.

Site occupancies were refined and then fixed at the first decimal place. In general non-hydrogen atom sites were modelled with anisotropic displacement parameters. A residual electron density peak near the disordered t-butyl residue sites of the **1**.HBr structure was treated as a partially occupied water site and modelled with an isotropic displacement parameter. A riding atom model with group displacement parameters was used for the hydrogen atoms. No hydrogens were included in the **1**.HBr model for the site treated as that of a partially occupied water site. Notwithstanding the disorder, the protonation site was evident in final difference maps (see **Figure S73**) for the **1**.HBr structure and is located at one of the pyridyl nitrogen sites. Preferential protonation of one pyridyl rather than the second is presumably dictated by the orientation of the triazole.

Table S1. Crystallographic details for **1**, **1**.HBr, **1**.HBF_4 and **1**.HCl

	**1**	**1**.HBr	**1**.HBF_4	**1**.HCl
Refinement model formula	$C_{76}H_{78}N_8O_5$	$C_{76}H_{79}BrN_8O_{5.2}$	$C_{76}H_{79}BF_4N_8O_5$	$C_{76}H_{79}ClN_8O_5$
Model molecular weight	1183.46	1267.58	1271.28	1219.92
Crystal System and space group	triclinic, $P\bar{1}$(#2)	triclinic, $P\bar{1}$(#2)	triclinic, $P\bar{1}$(#2)	triclinic, $P\bar{1}$(#2)
a	13.8653(8) Å	12.5548(3) Å	12.6911(3) Å	12.5002(2) Å
b	15.7188(8) Å	18.1946(5) Å	17.3702(5) Å	17.8989(2) Å
c	18.0898(7) Å	18.7081(5) Å	19.0346(5) Å	18.7000(3) Å
α	104.124(4)º	102.898(2)º	100.235(3)º	102.7550(10)º
β	94.099(4)º	96.066(2)º	96.419(2)º	98.1480(10)º
γ	114.898(5)º	98.010(2)º	97.845(2)º	97.0540(10)º
V (Å^3)	3399.3(3)	4083.39(19)	4050.7(2)	3987.71(10)
Z and D_c	2; 1.156 gcm^{-3}	2; 1.031 gcm^{-3}	2; 1.042 gcm^{-3}	2: 1.016 gcm^{-3}
Crystal Size (mm)	0.040x0.020x0.010	0.334x0.158x0.057	0.05x0.04x0.02	0.1x0.09x0.04
Crystal colour and habit	yellow needle	very pale yellow blade	yellow needle	yellow block
Temperature and λ(Cu Kα)	100(1)K; 0.71073 Å	150(1)K; 1.5418 Å	100(1)K; 1.5418 Å	100(1)K; 1.5418 Å
μ(Cu Kα) and T(multi-scan)$_{min, max}$	0.073 mm^{-1}; 0.876, 1.00	1.060 mm^{-1}; 0.604, 1.00	0.584 mm^{-1}; 0.759, 1.00	0.804; 0.851, 1.00
$2\theta_{max}$ and completeness	64.14º; 91.8%	136.5º; 96.4%	141.58º; 97.0%	141.78º; 97.2%
hkl range	-19 19, -23 22, -26 24	-15 15, -21 21, -22 22	-12 15, -20 20, -22 22	-14 14, -21 21, -22 22
N, N_{ind} and R_{merge}	80,495; 21,801; 0.1326	98,353; 14,428; 0.0511	60,879; 15,147; 0.0628	58, 570; 14,945; 0.0461
N_{obs} (I > 2σ(I)) and N_{var}	7, 624; 808	12,402; 869	6,916; 1084	8172; 949
Residuals[a] $R1$(F>2σ(F)), $wR2$(F^2;all)	0.1140, 0.2446	0.0726, 0.1909	0.1086, 0.2894	0.1688, 0.4265
GoF(all)	1.141	1.210	1.155	1.396
Residual Extrema (e$^-$ Å^{-3})	-0.329, 0.801	-1.278, 1.505	-0.258, 1.051	-0.760, 0.960

[a]Refined against SQUEEZED data

Table S2. Selected hydrogen bond geometry for **1**

Donor	Hydrogen	Acceptor	D-H(Å)	H-A(Å)	D-A(Å)	DHA Angle(º)
N2_1	H2N_1	N1_2	0.88	2.45	3.251(4)	152.1
N2_1	H2N_1	N2_2	0.88	2.59	3.201(4)	127.7
N3_1	H3N_1	N2_2	0.88	2.32	3.142(4)	156.0
N3_1	H3N_1	N1_2	0.88	2.87	3.387(4)	119.0

Table S3. Selected hydrogen bond/contact geometry for **1**.HBF_4

Donor	Hydrogen	Acceptor	D-H(Å)	H-A(Å)	D-A(Å)	DHA Angle(º)
N2A_1	H2NA_1	F1	0.88	1.99	2.83(3)	159.5
N3_1	H3N_1	F1	0.88	2.16	2.969(5)	153.3
C7A_1	H7A_1	F1	0.95	2.16	3.102(13)	172.2
N2B_1	H2NB_1	F1	0.88	2.32	3.12(2)	150.6
C11_2	H11A_2	F1	0.99	2.54	3.378(9)	141.9
C19_2	H19_2	F1	0.95	2.56	3.402(7)	147.3
C7B_1	H7B_1	F1	0.95	2.60	3.543(16)	171.9
N2A_1	H2NA_1	F2	0.88	2.42	3.05(3)	128.9
N2B_1	H2NB_1	F2	0.88	2.55	3.19(3)	129.4
N3_1	H3N_1	F2	0.88	2.65	3.246(5)	126.3
C20_2^{i}	H20A_2^{i}	F2	0.99	2.46	3.174(8)	128.6
C28_1^{i}	H28B_1^{i}	F2	0.99	2.52	3.456(6)	157.9
C23_2^{i}	H21A_2^{i}	F2	0.99	2.64	3.348(7)	128.9
C21_2^{i}	H21A_2^{i}	F2	0.99	2.93	3.437(8)	112.9
C7A_1	H7A_1	F2	0.95	3.18	3.906(16)	134.6
C23_2^{i}	H23A_2^{i}	F2	0.99	3.27	3.348(7)	85.91
C7A_1	H7A_1	F3	0.95	2.92	3.665(14)	136.3
C7B_1	H7B_1	F3	0.95	2.91	3.666(14)	137.5
C3_2^{ii}	H3_2^{ii}	F3	0.95	2.26	3.175(6)	160.6
C25B_1^{iii}	H25B_1^{iii}	F3	0.95	2.41	3.072(4)	126.8
C29_2^{i}	H29_2^{i}	F3	0.95	2.53	3.47(6)	175.4
C23_2^{i}	H23A_2^{i}	F3	0.99	2.89	3.496(7)	120.7
C23_2^{i}	H23B_2^{i}	F3	0.99	3.18	3.496(7)	100.4
C18_2	H18_2	F4	0.95	2.63	3.387(7)	137.0
C19_2	H19_2	F4	0.95	3.09	3.628(8)	101.5
C2_2^{ii}	H2_2^{ii}	F4	0.95	2.26	2.986(7)	133.1
C28_1^{i}	H28B_1^{i}	F4	0.99	2.78	3.220(6)	107.6
C3_2^{ii}	H3_2^{ii}	F4	0.95	2.81	3.278(8)	111.5
C28_1^{i}	H28A_1^{i}	F4	0.99	2.93	3.220(6)	98.16
N3_1	H3N_1	F4	0.88	3.14	3.975(5)	158.1
C14A_1	H14A_1	O1A_1	1.00	2.39	2.984(15)	117.2
C9_2	H9_2	O2A_1^{iii}	0.95	2.81	3.627(14)	144.2
C9_2	H9_2	O2B_1^{iii}	0.95	2.94	3.856(16)	162.4
C4A_1	H4A_1	O3_1	0.95	2.11	2.74(2)	122.7
C4B_1	H4B_1	O3_1	0.95	2.40	3.01(2)	121.6
C32_2	H32B_2	O3_1	0.99	2.92	3.434(7)	113.3
C32_2	H32A_2	O3_1i^{v}	0.99	2.51	3.490(7)	170.7
C36_1	H36_1	O2_2	0.95	2.91	3.833(7)	164.6
C20_2	H20B_2	N2B_1^{i}	0.99	3.08	3.70(4)	121.8
C21_2	H21A_2	N2B_1^{i}	0.99	3.03	3.74(3)	129.6
C11_2	H11B_2	N3_1	0.99	3.18	3.830(9)	124.5
N1_2	H1N_2	N4_1	0.88	2.05	2.895(6)	161.4
C11_2	H11B_2	N4_1	0.99	2.83	3.705(9)	148.0
C32_2	H32B_2	N4_1	0.99	2.55	3.259(7)	128.1
N1_2	H1N_2	N5_1	0.88	2.85	3.629(6)	149.1
C11_2	H11B_2	N5_1	0.99	3.03	3.832(10)	138.5
C11_2	H11B_2	N6_1	0.99	3.15	4.013(9)	146.3

^{i}-x+2, -y, -z+1; ^{ii}x+1, y, z; ^{iii}-x+2, -y+1, -z+1;^{iv}-x+1, -y, -z+1

Table S4. Selected hydrogen bond/contact geometry for 1.HCl

Donor	Hydrogen	Acceptor	D-H(Å)	H-A(Å)	D-A(Å)	DHA Angle(º)
N2A_1	H2NA_1	Cl1	0.88	2.24	3.096(9)	163.3
N3_1	HN3_1	Cl1	0.88	2.42	3.235(7)	154.7
C7A_1	H7A_1	Cl1	0.95	2.59	3.504(5)	161.7
C7B_1	H7B_1	Cl1	0.95	2.76	3.664(6)	159.7
C3_2[i]	H3_2[i]	Cl1	0.95	2.76	3.616(13)	149.8
N2B_1	H2NB_1	Cl1	0.88	2.81	3.488(10)	134.6
C28_1[ii]	H28A_1[ii]	Cl1	0.99	2.82	3.635(9)	140.0
C19_2	H19_2	Cl1	0.95	2.92	3.723(7)	142.9
C23_2[ii]	H23B_2[ii]	Cl1	0.99	2.95	3.72(2)	135
C25B_1[iii]	H25B_1[iii]	Cl1	0.95	3.12	3.79(5)	129
C29_2[ii]	H29_2[ii]	Cl1	0.95	3.13	4.041(12)	160.6
N1_2	H1N_2	N4_1	0.88	2.07	2.900(13)	158.0
N1_2	H1N_2	N5_1	0.88	2.80	3.576(11)	148.6
C20_2[ii]	H20B_2[ii]	N2A_1	0.99	2.48	3.16(3)	125.4
C21_2[ii]	H21B_2[ii]	N2A_1	0.99	2.75	3.32(3)	116.9
C20_2[ii]	H20B_2[ii]	N2B_1	0.99	2.80	3.51(3)	129.3
C21_2[ii]	H21B_2[ii]	N2B_1	0.99	2.96	3.56(3)	119.6
C22_2	H22B_2	O1_2	0.99	2.32	2.83(3)	110.7

[i]-x+2, -y, -z+1; [ii]x+1, y, z; [iii]-x+2, -y+1, -z+1

Table S5. Selected hydrogen bond/contact geometry for 1.HBr

Donor	Hydrogen	Acceptor	D-H(Å)	H-A(Å)	D-A(Å)	DHA Angle(º)
N2A_1	H2NA_1	Br1	0.88	2.52	3.368(4)	162.5
N3_1	H3N_1	Br1	0.88	2.54	3.373(3)	158.7
N2B_1	H2NB_1	Br1	0.88	2.79	3.532(4)	143.36
C7B_1	H7B_1	Br1	0.95	2.79	3.699(2)	161.35
C3_2[i]	H3_2[i]	Br1	0.95	2.87	3.597(4)	133.98
C7A_1	H7A_1	Br1	0.95	2.90	3.821(2)	161.45
C19_2	H19_2	Br1	0.95	3.03	3.818(5)	141.04
C28_1[ii]	H28B_1[ii]	Br1	0.99	3.05	3.907(3)	145.01
C2_2[i]	H2_2[i]	Br1	0.95	3.15	3.729(4)	121.26
C23_2[ii]	H23B_2[ii]	Br1	0.99	3.22	3.737(4)	114.15
C20_2[ii]	H20A_2[ii]	Br1	0.99	3.26	3.936(4)	126.78
C23_2[ii]	H23A_2[ii]	Br1	0.99	3.34	3.737(4)	106.01
C14A_1	H14A_1	O1A_1	1.00	2.52	3.061(11)	113.9
C9_2	H9_2	O2A_1[iii]	0.95	3.13	3.936(7)	143.7
C11_2	H11A_2	O2A_1[iii]	0.99	3.31	4.137(7)	142.5
C9_2	H9_2	O2B_1[iii]	0.95	2.70	3.627(8)	166.9
C11_2	H11A_2	O2B_1[iii]	0.99	3.52	4.002(8)	112.6
C32_2	H32B_2	O3_1	0.99	2.89	3.461(4)	117.2
C20_2	H20B_2	O3_1	0.99	3.46	4.402(5)	160.7
C30_1	H30_1	O1_2	0.95	3.33	4.259(4)	166.4
C36_1	H36_1	O2_2	0.95	2.99	3.915(5)	164.0
C20_2	H20B_2	N2A_1[ii]	0.99	2.83	3.446(12)	120.7
C21_2	H21A_2	N2A_1[ii]	0.99	2.85	3.512(13)	125.3
C21_2	H21A_2	N2B_1[ii]	0.99	2.97	3.644(13)	126.6
C20_2	H20B_2	N2B_1[ii]	0.99	3.00	3.622(12)	122.1
C20_2	H20B_2	N3_1	0.99	3.44	4.098(5)	125.6
N1_2	H1N_2	N4_1	0.88	2.11	2.953(4)	160.3
C32_2	H32B_2	N4_1	0.99	2.50	3.240(5)	131.2
C11_2	H11B_2	N4_1	0.99	3.08	3.824(5)	132.6
N1_2	H1N_2	N5_1	0.88	2.87	3.645(4)	147.6
C32_2	H32B_2	N5_1	0.99	3.41	3.963(5)	117.2
C11_2	H11B_2	N5_1	0.99	3.42	3.983(6)	117.9
C11_2	H11B_2	N6_1	0.99	3.57	4.204(6)	124.0

[i]x+1, y, z; [ii]-x+2, -y, -z+1;[iii]-x+2, -y+1, -z+1

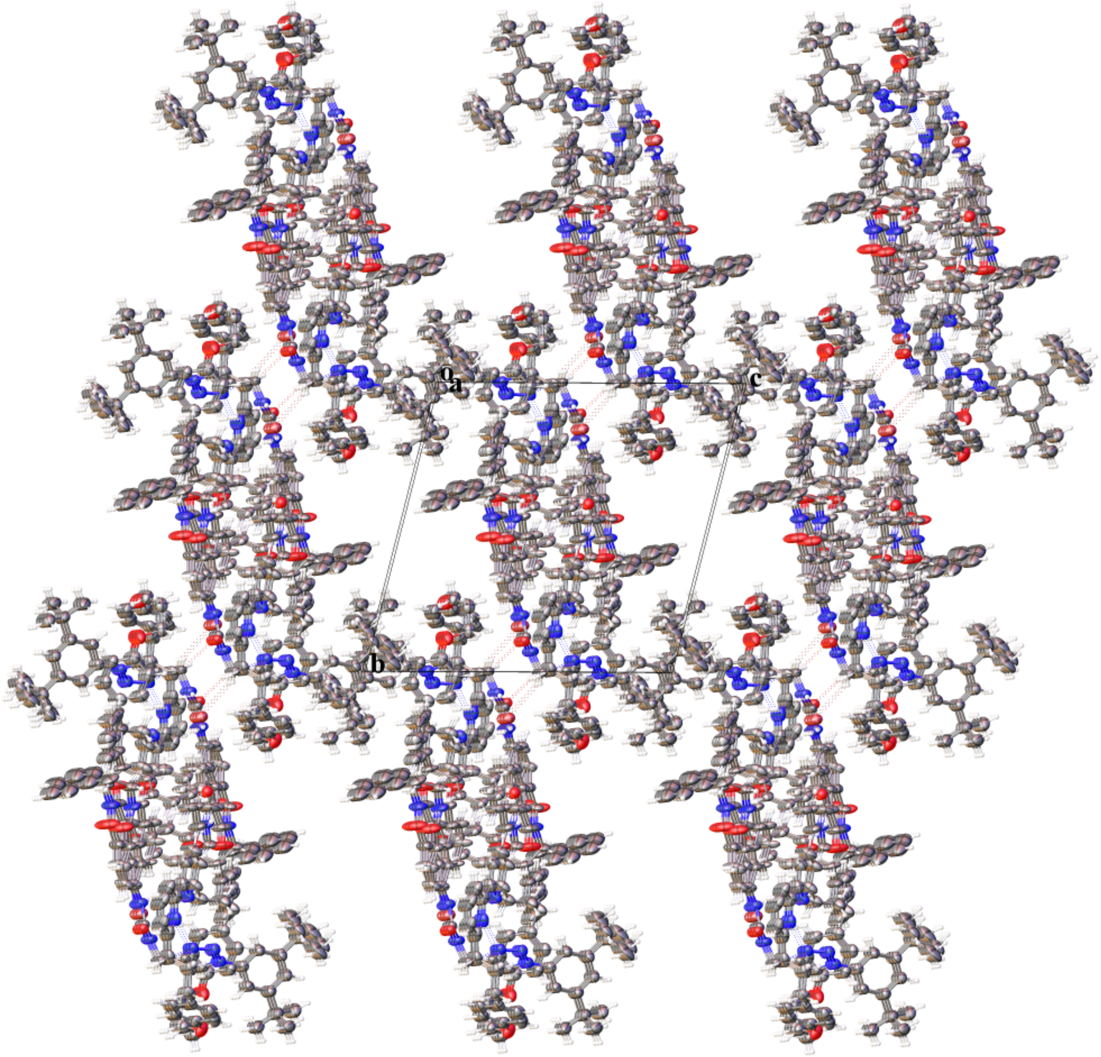

Figure S69. Depiction[5] of the crystal structure packing of rotaxane **1**.HBr viewed along the *a* axis and with displacement ellipsoids shown at the 50% level.

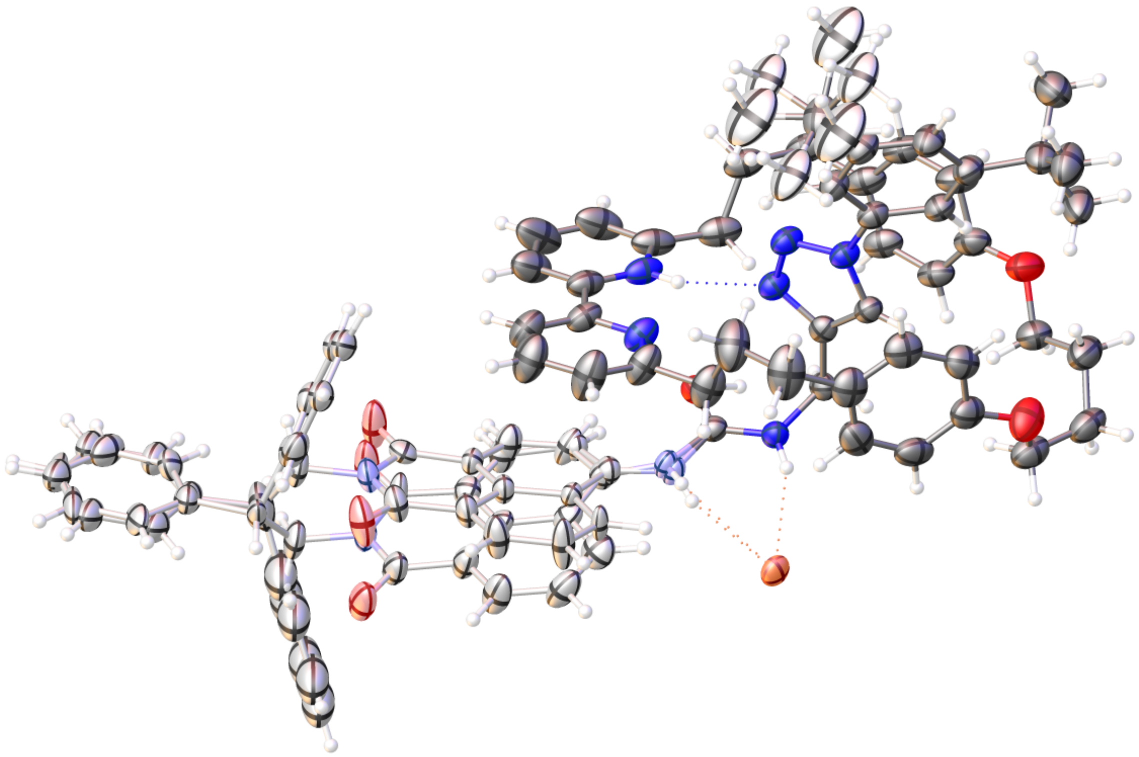

Figure S70. Depiction[5] of the rotaxane 1.HBr with displacement ellipsoids shown at the 50% level. Partially occupied sites are highlighted with 'faded' colours.

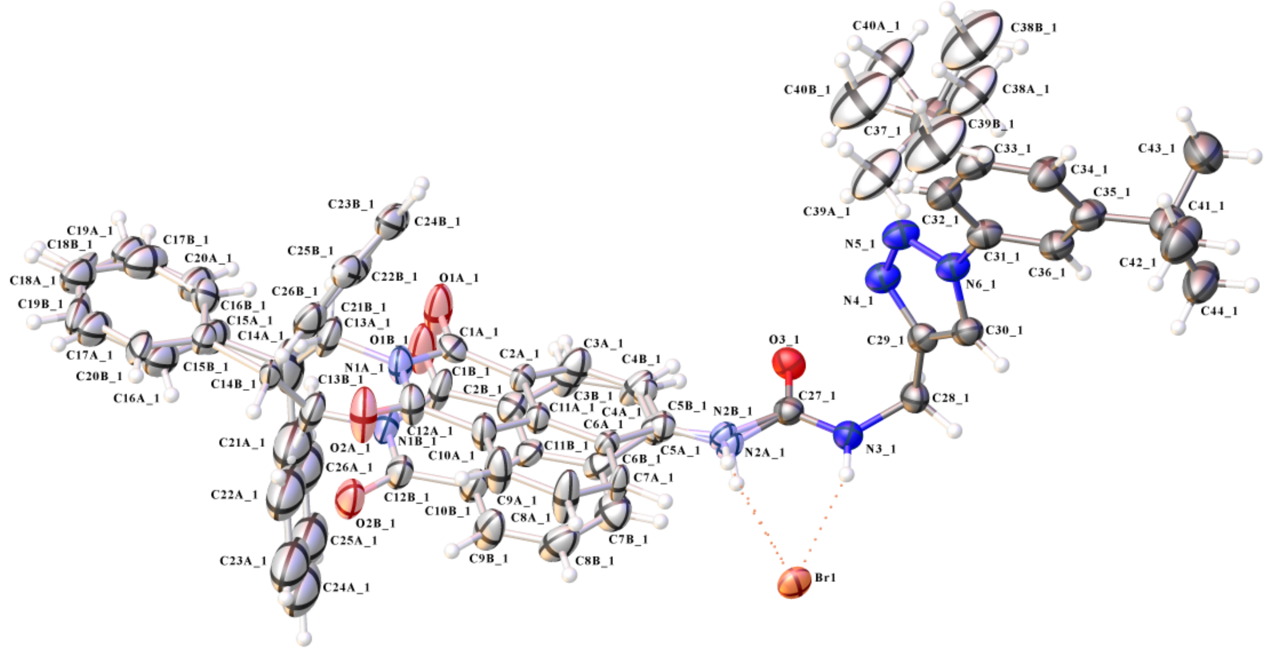

Figure S71. Depiction[5] of the 'axle' molecule of rotaxane **1**.HBr showing the numbering scheme and with displacement ellipsoids shown at the 50% level. Partially occupied sites are highlighted with 'faded' colours.

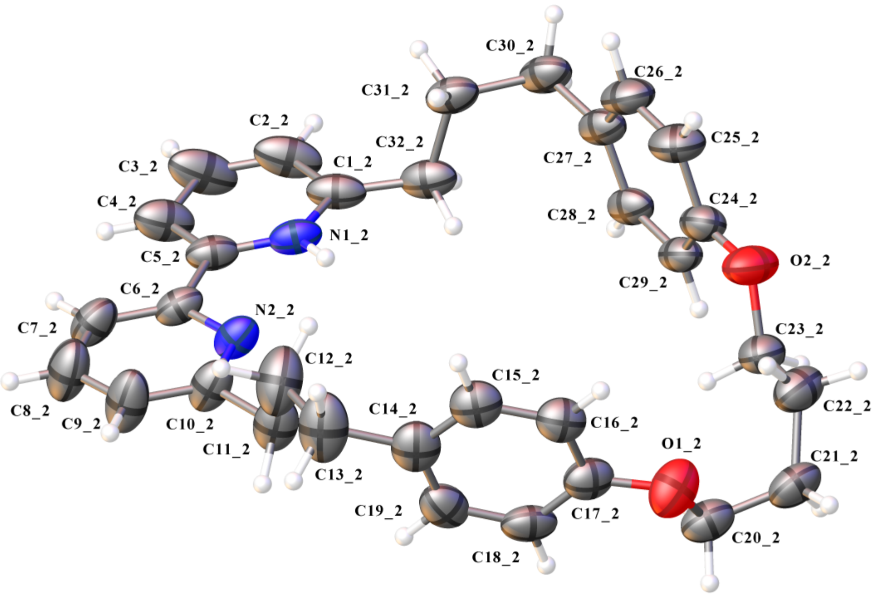

Figure S72. Depiction[5] of the macrocycle molecule of rotaxane **1**.HBr showing the numbering scheme and with displacement ellipsoids shown at the 50% level.

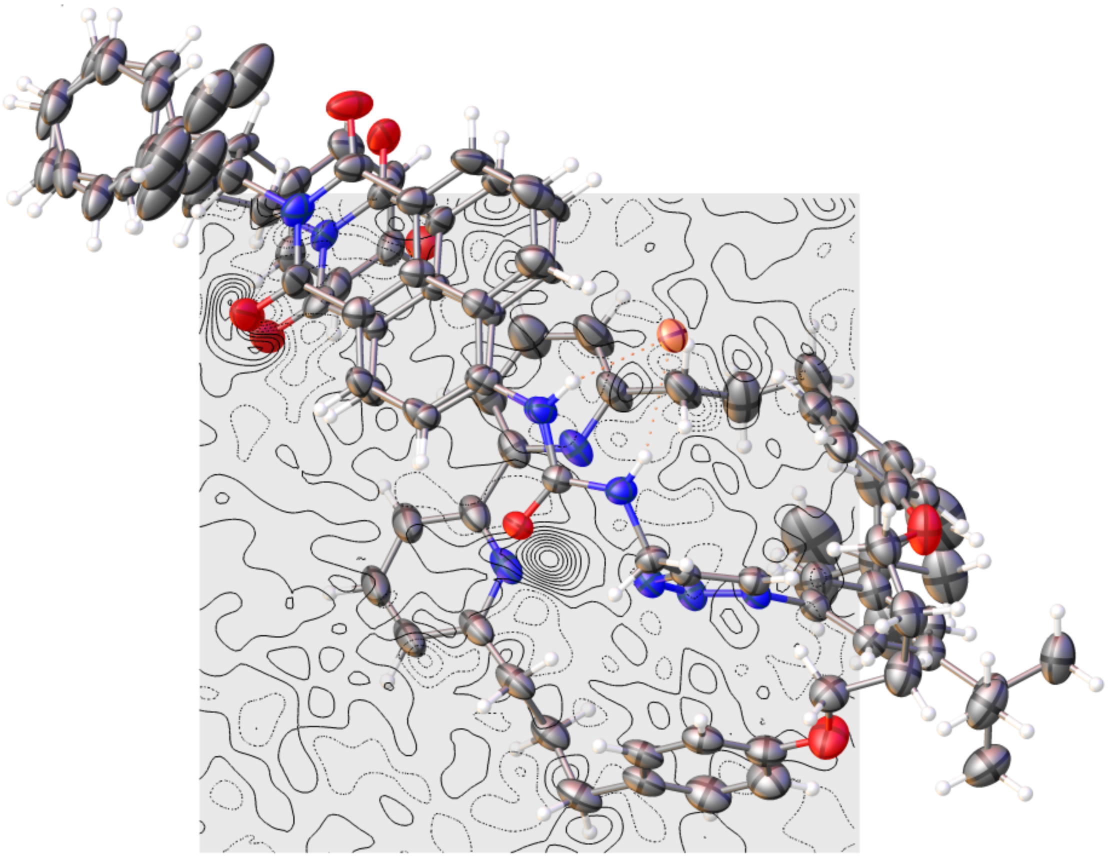

Figure S73. Depiction[5] of the electron density difference map contours highlighting the pyridyl protonation site for rotaxane **1**.HBr

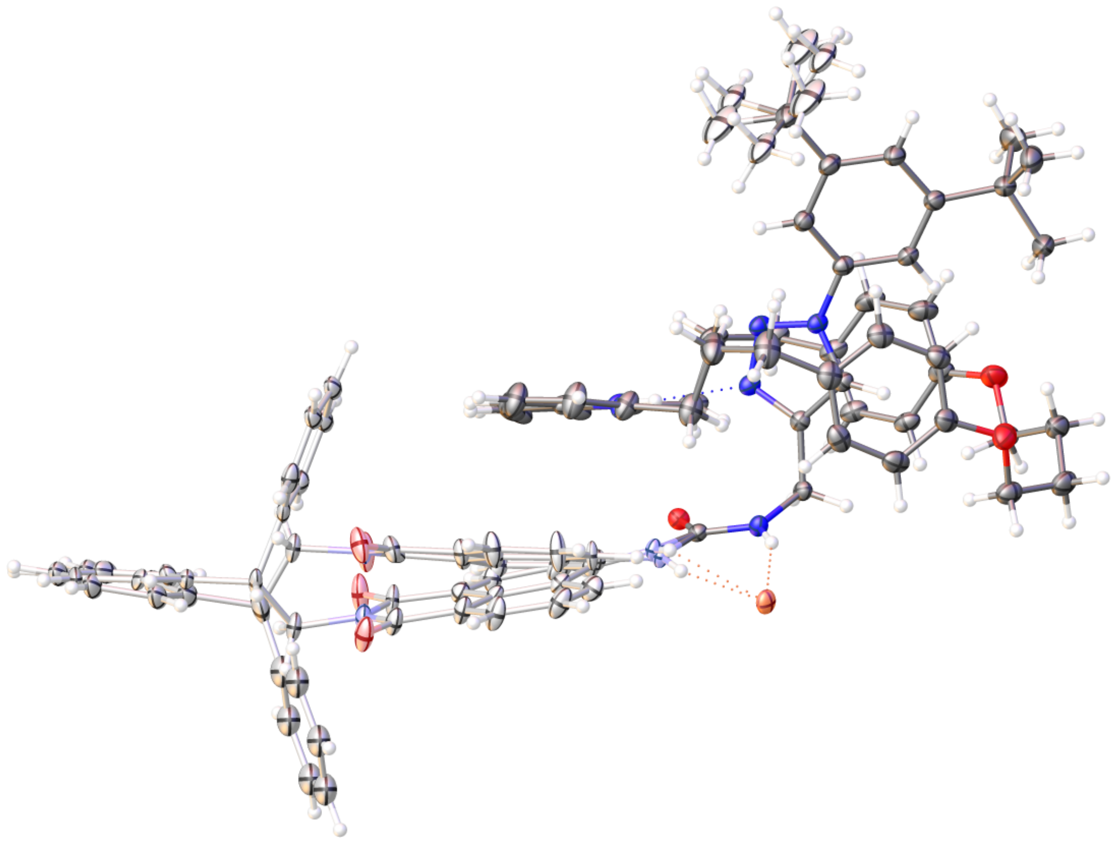

Figure S74. Depiction[5] rotaxane **1**.HBr along the bipyridyl axis, with displacement ellipsoids shown at the 25% level.

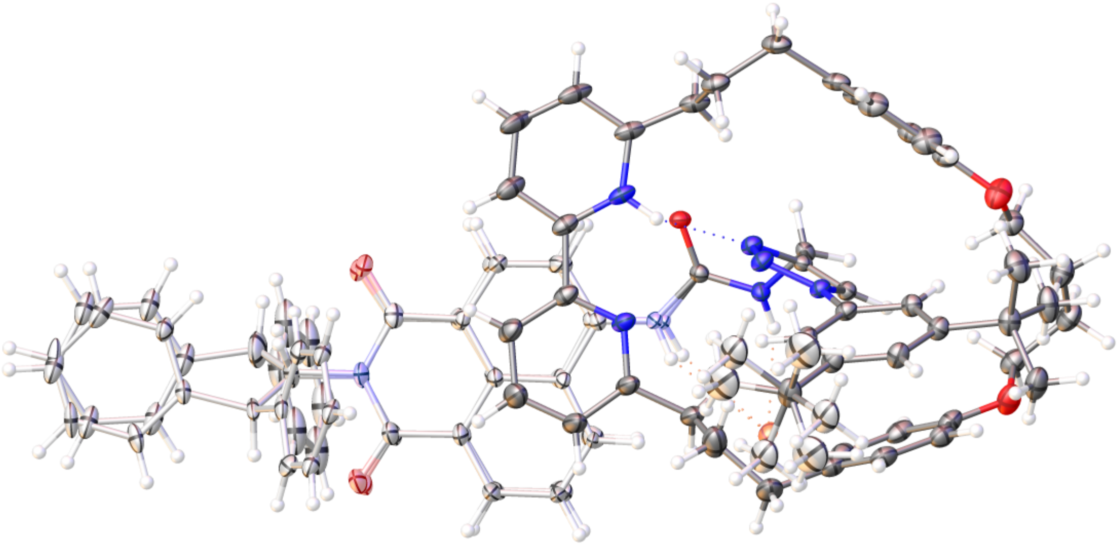

Figure S75. Depiction[5] of rotaxane **1**.HBr perpendicular to the bipyridyl of the macrocycle, with displacement ellipsoids shown at the 25% level.

Single Crystal X-ray Diffraction Characterisation of 2

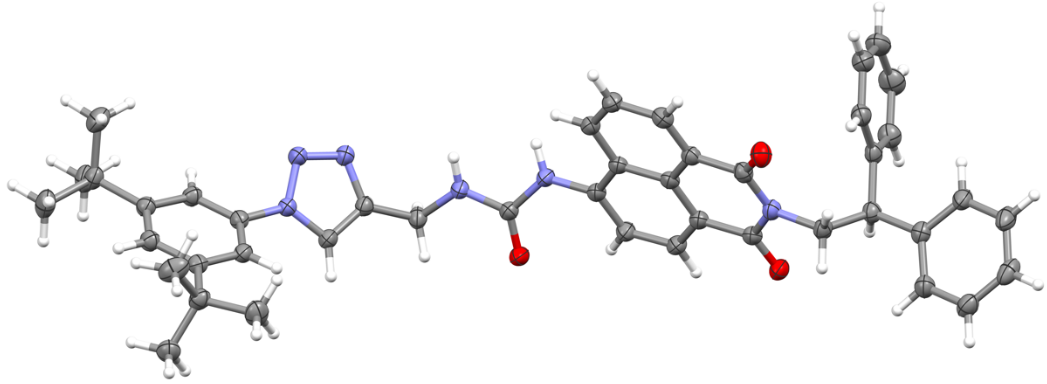

Figure S76. Depiction[5] of axle **2** with displacement ellipsoids shown at the 50% level.

Table S6 Crystal data and structure refinement for 2017_md_naphtha urea axle**.**

Identification code	2017_md_naphtha urea axle
Empirical formula	$C_{44}H_{44}N_6O_3$
Formula weight	704.85
Temperature/K	100(2)
Crystal system	triclinic
Space group	P-1
a/Å	9.8217(2)
b/Å	12.8166(3)
c/Å	15.7429(2)
α/°	72.122(2)
β/°	87.505(2)
γ/°	77.552(2)
Volume/Å^3	1841.15(7)
Z	2
ρ_{calc}g/cm^3	1.271
μ/mm^{-1}	0.645
F(000)	748.0
Crystal size/mm^3	0.08 × 0.08 × 0.01
Radiation	CuKα (λ = 1.54184)
2Θ range for data collection/°	7.946 to 140.962
Index ranges	-10 ≤ h ≤ 11, -15 ≤ k ≤ 15, -19 ≤ l ≤ 19
Reflections collected	23509
Independent reflections	6873 [R_{int} = 0.0373, R_{sigma} = 0.0344]
Data/restraints/parameters	6873/0/484
Goodness-of-fit on F^2	1.061
Final R indexes [I>=2σ (I)]	R_1 = 0.0395, wR_2 = 0.1035
Final R indexes [all data]	R_1 = 0.0450, wR_2 = 0.1074
Largest diff. peak/hole / e Å^{-3}	0.17/-0.19

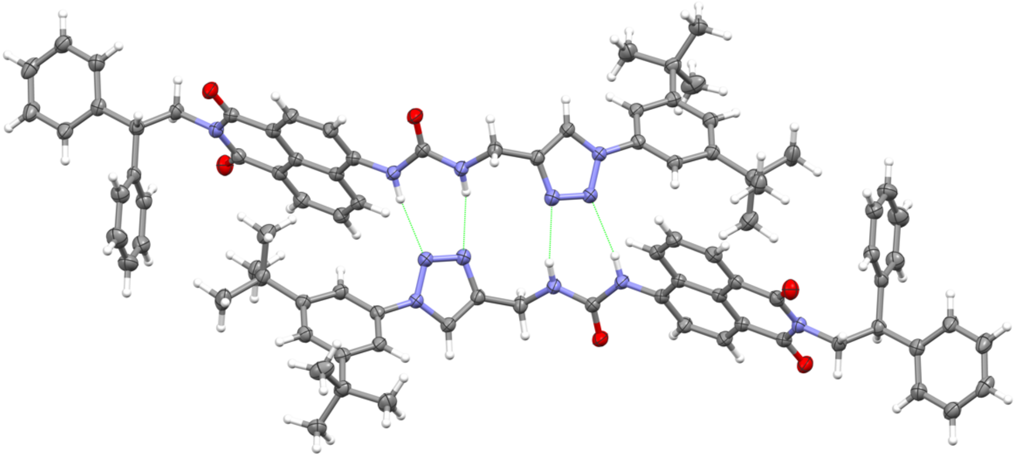

Figure S77. Depiction[5] of axle **2** showing the intramolecular interactions between two axle molecules in the solid state. Displacement ellipsoids shown at the 50% level.

References

[1] R. S. Stoll, M. V. Peters, A. Kuhn, S. Heiles, R. Goddard, M. Bühl, C. M. Thiele, S. Hecht, *J. Am. Chem. Soc.*, 2009, **131**, 357-367.

[2] J. E. M. Lewis, R. J. Bordoli, M. Denis, C. J. Fletcher, M. Galli, E. A. Neal, E. M. Rochette, S. M. Goldup, *Chem. Sci.*, 2016, **7**, 3154–3161.

[3] *CrysAlis Pro*, Rigaku Oxford Diffraction, Yarnton, Oxfordshire, England, **2015**

[4] L. J. Farrugia, *J. Appl. Crystallogr.* **2012**, *45*, 849–854.

[5] L. J. Farrugia, *J. Appl. Crystallogr.* **1999**, *32*, 837–838

[6] C. B. Hübschle, G. M. Sheldrick, B. Dittrich, *J. Appl. Crystallogr.* **2011**, *44*, 1281–1284.

[7] O. V. Dolomanov, L. J. Bourhis, R. J. Gildea, J. A. K. Howard, H. Puschmann, *J. Appl. Crystallogr.* **2009**, *42*, 339–341.

[8] L. Palatinus, G. Chapuis, *J. Appl. Crystallogr.* **2007**, *40*, 786–790

[9] G. M. Sheldrick, *Acta Crystallogr. Sect. C Struct. Chem.* **2015**, *71*, 3–8

[10] S. R. Hall, D. J. du Boulay, R. Olthof-Hazekamp, *Xtal3.7*, University Of Western Australia, **2000**

[11] C. R. Groom, I. J. Bruno, M. P. Lightfoot, S. C. Ward, *Acta Crystallogr. Sect. B Struct. Sci. Cryst. Eng. Mater.* **2016**, *72*, 171–179.

[12] G. M. Sheldrick, *Acta Crystallogr. Sect. Found. Adv.* **2015**, *71*, 3–8
